# Structural basis of Notch O-glucosylation and O–xylosylation by mammalian protein–O-glucosyltransferase 1 (POGLUT1)

**DOI:** 10.1038/s41467-017-00255-7

**Published:** 2017-08-04

**Authors:** Zhijie Li, Michael Fischer, Malathy Satkunarajah, Dongxia Zhou, Stephen G. Withers, James M. Rini

**Affiliations:** 1 Department of Biochemistry, University of Toronto, Toronto, ON Canada M5S 1A8; 2 Department of Molecular Genetics, University of Toronto, Toronto, ON Canada M5S 1A8; 3 Department of Chemistry, University of British Columbia, Vancouver, BC Canada V6T 1Z1

## Abstract

Protein *O*-glucosyltransferase 1/Rumi-mediated glucosylation of Notch epidermal growth factor-like (EGF-like) domains plays an important role in Notch signaling. Protein *O*-glucosyltransferase 1 shows specificity for folded EGF-like domains, it can only glycosylate serine residues in the C^1^XSXPC^2^ motif, and it possesses an uncommon dual donor substrate specificity. Using several EGF-like domains and donor substrate analogs, we have determined the structures of human Protein *O*-glucosyltransferase 1 substrate/product complexes that provide mechanistic insight into the basis for these properties. Notably, we show that Protein *O*-glucosyltransferase 1’s requirement for folded EGF-like domains also leads to its serine specificity and that two distinct local conformational states are likely responsible for its ability to transfer both glucose and xylose. We also show that Protein *O*-glucosyltransferase 1 possesses the potential to xylosylate a much broader range of EGF-like domain substrates than was previously thought. Finally, we show that Protein *O*-glucosyltransferase 1 has co-evolved with EGF-like domains of the type found in Notch.

## Abstract (web summary)

POGLUT1 is a protein-O-glucosyltransferase that transfers glucose and xylose to the EGF-like domains of Notch and other signaling receptors. Here the authors report the structure of human POGLUT1 in complexes with 3 different EGF-like domains and donor substrates and shed light on the enzyme’s substrate specificity and catalytic mechanism

## Introduction

**Fig. 1:**
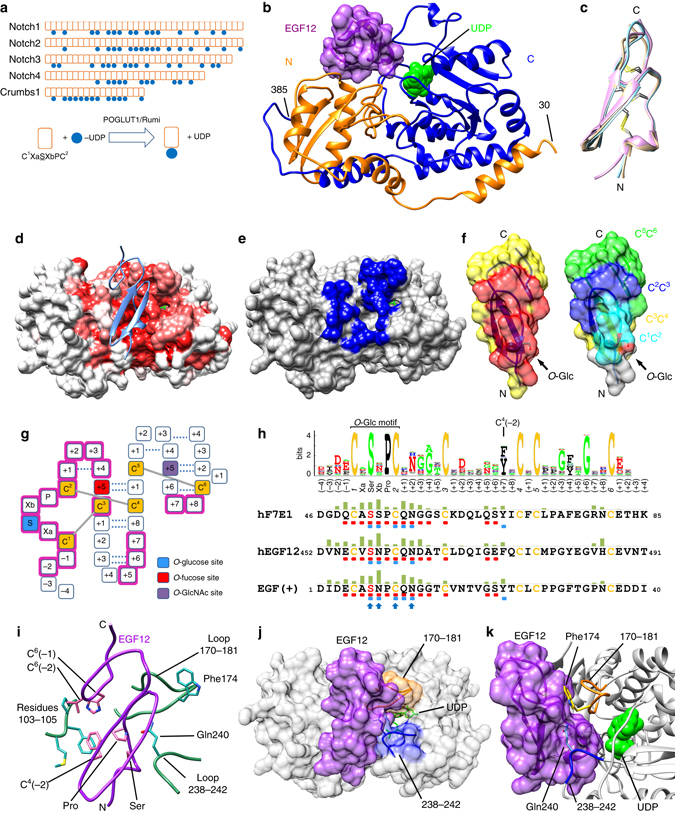
The overall structure of the POGLUT1-EGF complexes. **a** POGLUT1 catalyzes the transfer of a glucose moiety (*blue circle*) to EGF-like domains (*red box*) containing the *O*-glucosylation motif C^1^XaSXbPC^2^. Human proteins with 10 or more *O*-glucosylation sites are shown. **b** The POGLUT1/hEGF12/UDP complex with POGLUT1 shown in ribbon representation. *Orange*, residues 30–169, *blue*, residues 170–385. The N-terminal and C-terminal segments each insert into the opposite domain. **c** The three co-crystallized EGF-like domains structurally aligned on POGLUT1: *pink*, EGF(+); *cyan*, hF7EGF1; *ivory*, hEGF12. **d** Conserved surface of POGLUT1. *Deeper redness* indicates higher conservation. **e** EGF-like domain-contacting surface (*blue*) of POGLUT1. **f** Comparison of POGLUT1-contacting surface (*red surface*, *left*) and location of the cysteine-delimited segments (*right*) of EGF(+). **g** EGF-like domain residue numbering. *Gray lines* show the three conserved disulfide bonds. Residues in contact with POGLUT1 are shaded *magenta*. **h** A scheme summarizing the interactions found in the three EGF-like domain complexes. Buried surface areas upon complex formation are plotted above each residue as heights of the green bars. *Red dots* below residues indicate backbone interactions; *blue dots* indicate hydrogen bonds. The four *arrow heads* indicate residues making conserved hydrogen bonds (C^2^ makes two of them). The Sequence logo at the top was generated from ~40,600 *O*-glucosylation motif-containing EGF-like domains found in 339 animal species. **i** Interactions of the EGF-like domain C^4^(−2)/C^6^(−2)/C^6^(−1) patch with POGLUT1 residues 103–105. The 170–181 and 238–242 loops and two residues making significant interactions with the EGF-like domains are also shown. **j** Locations of the 170–181 and 238–242 loops. **k** The burial of UDP (*green*) by the 170–181 loop (*orange*) and the EGF-like domain

Protein *O*-glucosyltransferase 1 (POGLUT1) glucosylates the epidermal growth factor-like (EGF-like) domains found in diverse substrates including Notch and its ligands^1–3^ (Fig. 1a). Including work done on its *Drosophila* homolog, Rumi^2^, it has been well established that *O*-glucosylation by this enzyme is critical for development and Notch signaling^2, 4^. More recently, the action of POGLUT1/Rumi on the Eyes shut protein was shown to promote rhabdomere separation in *Drosophila* ^5^ and its action on the mammalian Crumbs2 protein was shown to promote mouse gastrulation^6^. In humans, defects in POGLUT1 have been found to cause Dowling–Degos disease^7^ and a form of limb-girdle muscular dystrophy associated with decreased Notch signaling^8^. Like the EGF-like domain *O*-fucosylation enzyme POFUT1^9^, POGLUT1 only acts on folded EGF-like domains^2, 10^, a property thought to reflect a role in protein folding and/or quality control^11^. Indeed, glucosylation by Rumi has varying effects on the folding, thermal stability, and trafficking/secretion of Notch, Crumbs, and Eyes shut^5, 12, 13^. Unlike most other glycosyltransferases, POGLUT1 displays dual donor substrate specificity as it can utilize both UDP-glucose and UDP-xylose, a property leading to the incorporation of both xylose and glucose into Notch proteins^14^. Moreover, unlike other protein-*O*-glycosyltransferases that modify both serine and threonine, POGLUT1 can only utilize serine^3, 15^. Recently, structures of *Drosophila* Rumi (dRumi) in complex with human Factor IX EGF-like domain 1 (hF9EGF1) and UDP have provided insight into EGF-like domain recognition and the possible effects of disease causing mutations^16^.

Here we report the structure of human POGLUT1 in complexes with three different EGF-like domains and either UDP, a donor substrate analog or a slow substrate. The structures reveal the basis for POGLUT1’s ability to recognize diverse EGF-like domain substrates and for the requirement that they be folded. We also show that POGLUT1’s serine specificity stems from the backbone conformation of the glycosylation motif and, as such, its requirement for a folded EGF-like domain substrate. POGLUT1 was found to access two local conformational states and we provide evidence to support the suggestion that they are individually responsible for the transfer of UDP-glucose and UDP-xylose. We also show that POGLUT1 does not require a diserine-containing glycosylation motif to efficiently xylosylate its substrates, an observation suggesting that it can xylosylate a much wider range of EGF-like domains than previously thought. Finally, we show that the POGLUT1 glycosylation motif is found predominantly among EGF-like domains of the type found in Notch and that POGLUT1 has co-evolved with these domains through animal evolution.

## Results

### Overall structure of the hPOGLUT1—EGF-like domain complexes

The X-ray crystal structure of hPOGLUT1 was determined in complex with human factor VII EGF-like domain 1 (hF7EGF1) and human Notch1 EGF-like domain 12 (hEGF12), as well as a synthetic POGLUT1 substrate, EGF(+), the consensus sequence for human EGF-like domains that are POFUT1 substrates^17^ (Supplementary Table 1, Supplementary Fig. 4). POGLUT1 is an inverting GT-B fold (CAZy GT Family 90^18^) glycosyltransferase and its EGF-like domain substrates sit in a cleft formed between the canonical N-terminal and C-terminal domains (Fig. 1b). The three EGF-like domains studied are positioned almost identically in their respective complexes and 25–30% of their surface (740–830 Å^2^) is buried by POGLUT1 (Fig. 1c, Supplementary Fig. 5). The interface between the EGF-like domains and POGLUT1 is highly complementary, with Sc scores^19^ ranging from 0.75 to 0.80. Multiple points of contact and extensive buried surface clearly establish the basis for POGLUT1’s requirement for a folded EGF-like domain (Fig. 1d–f).

Three regions of the EGF-like domain are primarily responsible for the interaction with POGLUT1: (i) the C^1^C^2^ loop that corresponds to the *O*-glucosylation motif (C^1^XaSXbPC^2^), (ii) the C^2^C^3^ loop that is the site of the POFUT1 *O*-fucosylation motif (C^2^XXXXS/TC^3^), and (iii) a patch formed by residues C^4^(−2), C^6^(−1), and C^6^(−2) (Fig. 1f, g). The C^1^C^2^ and C^2^C^3^ loop interactions constitute ~38% and ~30%, respectively, of the EGF-like domain surface buried by POGLUT1 and are described in detail below. The C^4^(−2) residue is highly conserved among all animal EGF-like domains (Fig. 1h) and it is closely associated with the conserved proline residue of the glucosylation motif. Together these two residues make apolar interactions with POGLUT1 residues Met103, Phe104, and Pro105 (Fig. 1i) as is also observed for the equivalent residues in the dRumi-hF9EGF1 structure^16^. The C^6^(−1) and C^6^(−2) residues, variable among EGF-like domains, also interact with Phe104 and Pro105. Among the complexes reported here, the interactions involving the C^4^(−2), C^6^(−1), and C^6^(−2) residues constitute 10–16% of the EGF-like domain surface buried on complex formation and POGLUT1 residues 103–105 constitute 18–20% of its buried surface.

Two POGLUT1 loops (Loop 1, 238–242; Loop 2, 170–181) constitute over 50% of the POGLUT1 surface area buried by the bound EGF-like domain. Loop 1 folds over the glucosylation motif (C^1^C^2^ loop) of the EGF-like domain and Loop 2 interacts with the bound UDP/UDP-glucose and the C^2^C^3^ loop. As such, Loop 2 is sandwiched between the donor and the acceptor substrates (Fig. 1i–k).

### POGLUT1 interactions with the *O*-glucosylation motif

**Fig. 2:**
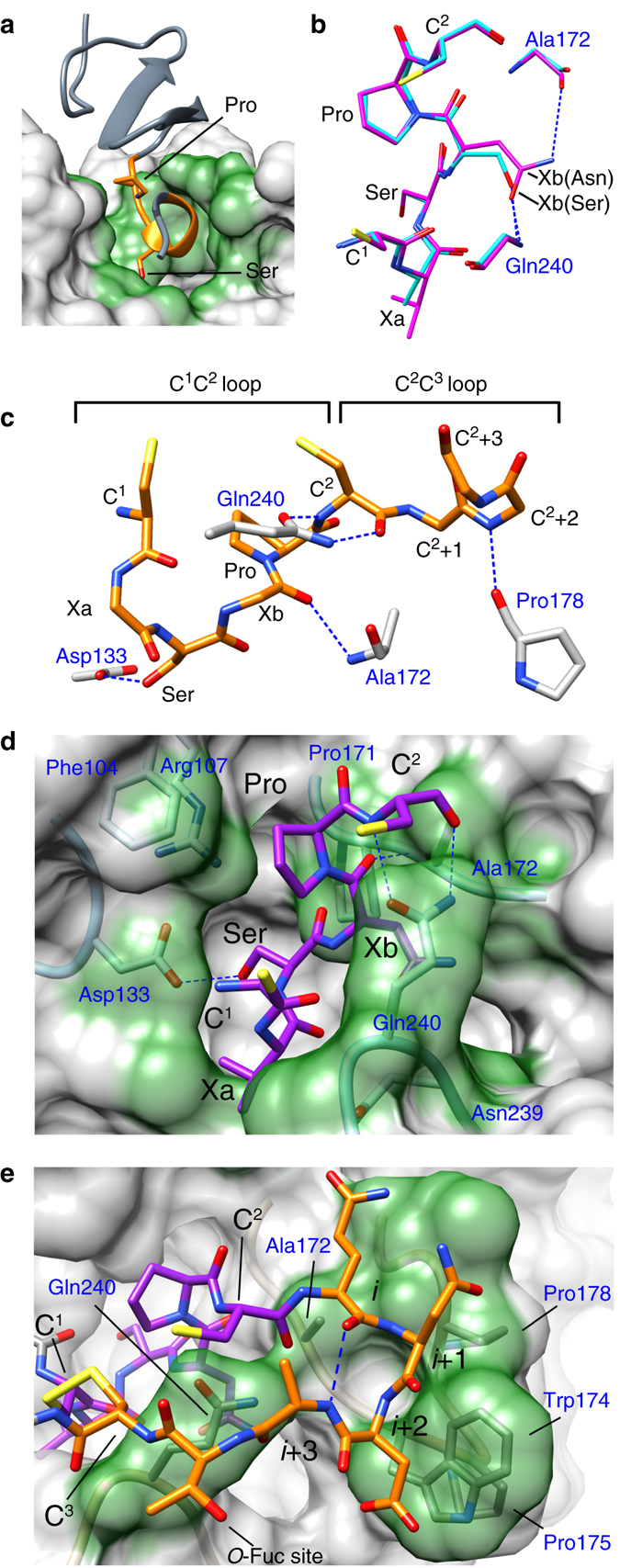
Recognition of the C^1^C^2^ and C^2^C^3^ loops. **a** The C^1^C^2^ loop (*orange*) is buried in POGLUT1. **b** Comparison of the C^1^XSNPC^2^ (hEGF12 complex; *magenta*) and C^1^XSSPC^2^ (hF7EGF1 complex; *cyan*) motifs, showing the Xb(Asn/Ser) sidechain hydrogen bonds. For clarity, the Ala172 and Gln240 sidechains are not shown. **c** The five conserved hydrogen bonds. For clarity, the sidechains of variable residues are not shown. **d** Binding surface (*green*) of the C^1^C^2^ loop (*purple*) in the POGLUT1/hEGF12/UDP complex. **e** Binding surface (*green*) of the C^2^C^3^ loop (*orange*) in the POGLUT1/hEGF12/UDP complex. *i*, *i* + 1, *i* + 2, and *i* + 3 mark the residues involved in the type I’ β-turn. The *i* + 2 position is a glycine in most other EGF-like domains

The glucosylation motif (C^1^XaSXbPC^2^) is buried in a deep cleft where it is largely shielded from solvent (Fig. 2a), an indication of the key role that it plays in POGLUT1 recognition. Residues C^1^, Xa, Ser, and Xb belong to a short 3_10_-helix that is terminated by the conserved Pro and the residues centered on the Pro make the most intimate contacts with POGLUT1. Among the three different EGF-like domains studied, there are five hydrogen bonds between POGLUT1 and the EGF-like domain that are common and four of them involve invariant atoms of the glucosylation motif (Fig. 2c). Of these, three involve backbone atoms of residues Xb and C^2^, which are hydrogen bonded to POGLUT1 residues Gln240 and Ala172. The fourth is a strong hydrogen bond between the hydroxyl group of the glucosylatable serine residue and Asp133, the POGLUT1 catalytic base^16^. The proline residue stabilizes the conformation required to make the observed backbone hydrogen bonds and it is sandwiched between POGLUT1 residues Phe104 and Gln240 (Fig. 2d). Of the two variable residues in the glucosylation motif (Supplementary Fig. 3), Xa is relatively exposed and its sidechain is directed toward bulk solvent. In contrast, the Xb residue is largely buried by POGLUT1 residue Gln240. Of the EGF-like domains studied, hEGF12 and EGF(+) contain an asparagine residue at the Xb position (C^1^XaSNPC^2^) while hF7EGF1 contains a serine (C^1^XaSSPC^2^). In all cases, the asparagine and serine sidechains make a strong hydrogen bond to the backbone NH group of Gln240 (Fig. 2b).

Comparison with the dRumi/hF9EGF1/UDP complex^16^ shows that the conformation of the hF9EGF1 domain and its glycosylation motif are very similar to that observed here (Supplementary Fig. 6). Moreover, analysis of structures deposited in the PDB show that the conformation of the glucosylation motif is very similar to that found in EGF-like domains in the absence of POGLUT1 (Supplementary Fig. 5), an indication that POGLUT1 does not appreciably distort the acceptor substrate.

### The C^2^C^3^ loop is a determinant of POGLUT1 recognition

As we now show, the C^2^C^3^ loop of the EGF-like domain, the site of the POFUT1 fucosylation motif (C^2^XXXXS/TC^3^), also serves as a determinant of recognition for POGLUT1. All of the EGF-like domains studied here possess the POFUT1 fucosylation motif and as the structures show, the fucosylatable Ser/Thr residue points to solvent. As such, the fucosylation state of the EGF-like domain would not be expected to significantly modulate its interaction with POGLUT1. The first four residues of the C^2^C^3^ loop form a type I′ β-turn and Loop 2 (residues 170–181) of POGLUT1 interacts with exposed backbone atoms of the first three. Of the five conserved hydrogen bonds between POGLUT1 and the EGF-like domains observed in our complexes, the fifth is between the NH group of the *i* + 1 residue (C^2^(+2)) of the β-turn and the backbone carbonyl oxygen atom of POGLUT1 residue Pro178 (Fig. 2c). Also notable are the interactions made by POGLUT1 residue Trp174 that is sandwiched between Pro175 and the peptide plane formed by the *i* + 1 and *i* + 2 residues of the β-turn. In the POGLUT1/hEGF12/UDP complex, the presence of the Asp sidechain at the *i* + 2 position of hEGF12 further extends the interaction surface with Trp174 (Fig. 2e). Interestingly, dRumi possesses a single lysine residue in place of Trp174 and Pro175 and the stacking interaction is not observed. Nevertheless, the hydrogen bond between the NH of the *i* + 1 residue and Pro178 is observed (Supplementary Fig. 6c). As shown in Table 1 and Supplementary Fig. 9, mutation of the 5-residue C^2^C^3^ loop of hEGF12 to 4-, 6-, and 7-residues leads to very poor substrates for both POGLUT1 and dRUMI, an indication that interactions with a five-membered C^2^C^3^ loop are important in both proteins.

**Table 1:** Kinetic parameters of POGLUT1 and dRumi with EGF-like domains and UDP-glucose (UG) or UDP-xylose (UX)

	Enzyme	Donor	*K*_M_^a^ (μM)	*V*_max_^a^ (μmol min^−1^ mg^−1^)	*n*	*V*_max_/*K*_M_ (μmol min^−1^ mg^−1^ μM^−1^)
hF7EGF1 (C^1^ASSPC^2^)	hPOGLUT1	UG	0.3 ± 0.1	0.20 ± 0.01	4	0.59 ± 0.08
	hPOGLUT1	UX	0.7 ± 0.2	0.19 ± 0.01	3	0.26 ± 0.06
hEGF12 *wt* (C^1^VSNPC^2^)	hPOGLUT1	UG	7.7 ± 1.5	0.18 ± 0.02	6	0.023 ± 0.005
	hPOGLUT1	UX	11 ± 1	0.10 ± 0.01	4	0.009 ± 0.001
	dRumi	UG	21 ± 3	0.48 ± 0.03	3	0.022 ± 0.003
hEGF12 N459S (C^1^VSSPC^2^)	hPOGLUT1	UG	5.3 ± 1.0	0.19 ± 0.01	5	0.036 ± 0.007
	hPOGLUT1	UX	6.6 ± 0.8	0.18 ± 0.01	4	0.028 ± 0.004
hEGF12 S458T (C^1^VTNPC^2^)	hPOGLUT1	UG	No reaction	0	3	
hEGF12 4-res C^2^C^3^	hPOGLUT1	UG	155 ± 10		3	
	dRumi	UG	160 ± 10		3	
hEGF12 6-res C^2^C^3^	hPOGLUT1	UG	Very poor (~1 mM)		3	
	dRumi	UG	Very poor (~1 mM)		3	
hEGF12 7-res C^2^C^3^	hPOGLUT1	UG	Very poor (~1 mM)		3	
	dRumi	UG	Very poor (~1 mM)		3	
	hPOGLUT1	UG	68 ± 10		3	
	hPOGLUT1	UX	62 ± 9		3	

^a^In all cases showing standard deviation of *n* repeats

### POGLUT1 predominantly modifies h-type EGF-like domains

EGF-like domains have been classified into four types and of these the hEGF and cEGF types (6-Cys-type domains) are the most common^20^. Strikingly, we found that 93.8% of the *O*-glucosylation motif-containing EGF-like domains are of the hEGF type (Supplementary Table 6, Supplementary Fig. 2a, b, Supplementary Data 1 and 2), the only EGF-like domain type found in Notch and its ligands. Moreover, we found that 84% of all hEGF domains possess a five-membered C^2^C^3^ loop, a property not shared by the cEGF domains (Supplementary Fig. 2d, e). The prevalence of a five-residue C^2^C^3^ loop among hEGF domains is particularly noteworthy given POGLUT1’s preference for a five-residue C^2^C^3^ loop and POFUT1’s strict dependence on a C^2^C^3^ loop of this length^17^. The glycosylation motifs for POGLUT1 and POFUT1 are found in ~25% (Supplementary Fig. 2b) and ~40%^17^ of all hEGF domains, respectively, and ~50% of POGLUT1 substrates are also POFUT1 substrates (Supplementary Table 6), a clear indication that hEGF domains and these enzymes are linked. Indeed, the presence of POFUT1 and hEGF domains has been found to accompany animal evolution^17^ and with the exception of a few parasitic organisms (Supplementary Table 5), we now show that all animal species sequenced also contain POGLUT1/Rumi homologs (Supplementary Data 1 and 2).

### POGLUT1 complexes with UDP-CH_2_-glucose and UDP-2F-glucose

**Fig. 3:**
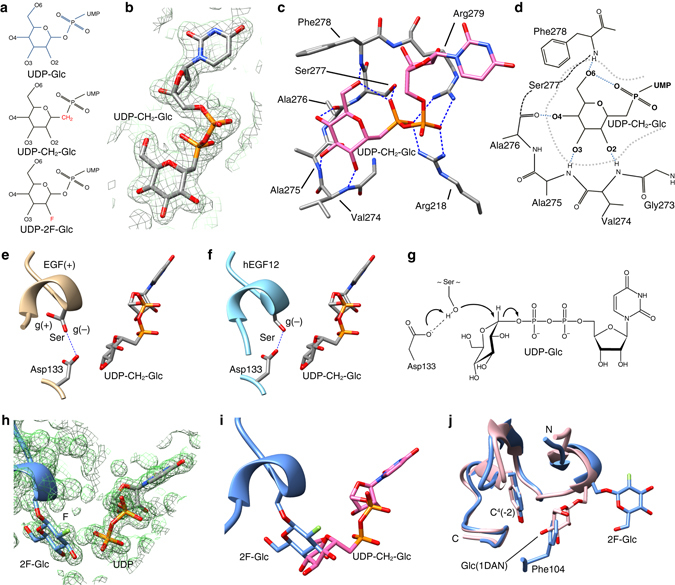
Donor substrate analog complexes. **a** The natural donor substrate, UDP-glucose, and the two donor analogs used for co-crystallization. The UDP-2F-glucose was found to be transferred resulting in the POGLUT1/EGF(+)-2F-glucose/UDP complex. **b** Composite omit map (2*mF*o–*DF*c) of the donor analog found in the 1.80 Å POGLUT1/EGF(+)/UDP-CH_2_-glucose complex, contoured at 1.50 RMSD (1.10 e Å^−3^). A 3 Å radius electron density mask was applied around the UDP-CH_2_-glucose for clarity. **c** The glucose subsite showing the hydrogen bonds. Two critical Arg residues interacting with both phosphates are also shown. **d** All hydrogen bonds involving the glucose moiety of UDP-CH_2_-glucose. **e** The EGF(+)/UDP-CH_2_-glucose complex showing the two rotamers (gauche (+) and gauche (−)) observed for the serine. **f** Superimposition of hEGF12 with the EGF(+)/UDP-CH_2_-glucose complex aligned on POGLUT1. **g** The S_*N*_2 catalytic mechanism of POGLUT1. **h** Composite omit map (2*mF*o–*DF*c) of the 1.31 Å POGLUT1/EGF(+)-2F-glucose/UDP product complex, contoured at 1.50 RMSD (1.20 e Å^−3^). A 3 Å radius electron density mask was applied around the UDP and the EGF(+)-2F-glucose for clarity. **i** Superimposition of the EGF(+)-2F-glucose (*blue*) product and the UDP-CH_2_-glucose donor analog (*magenta*). **j** Superimposition of the EGF(+)-2F-glucose (*blue*) and glucosylated hF7EGF1 (*pink*; PDB: 1DAN). POGLUT1 residue Phe104 interacts with the conserved aromatic C^4^(−2) residue in all POGLUT1/EGF complexes

Repeated attempts to obtain complexes of POGLUT1 with UDP-glucose or UDP-xylose invariably led to structures showing electron density for only the hydrolysis product, UDP. To gain further insight in the mechanism of catalysis and the dual donor substrate specificity shown by POGLUT1, we employed two different donor substrate analogs: (i) UDP-phosphono-glucose (UDP-CH_2_-glucose), a non-hydrolyzable analog^21^ and (ii) the slow substrate/inhibitor UDP-2-deoxy-2-fluoro-glucose (UDP-2F-glucose)^22^ (Fig. 3a). The structures of ternary complexes of POGLUT1, EGF(+) and each of the analogs were obtained. As shown in Fig. 3b and Supplementary Fig. 4, the POGLUT1/EGF(+)/UDP-CH_2_-glucose complex showed strong electron density for the entire UDP-CH_2_-glucose molecule. With the exception of the β-phosphate, the position and conformation of the nucleoside diphosphate moiety of this analog is essentially the same as that of the UDP in all of the UDP-containing ternary complexes. The methylene linker and glucose moieties are well ordered and the 2-, 3-, and 4-hydroxyls of the glucose make hydrogen bonds with backbone atoms of POGLUT1 residues, Val274, Ala275, and Ala276 of the 272–276 turn. The C6-hydroxyl group donates a strong hydrogen bond (2.84 Å) to a β-phosphate oxygen atom and simultaneously accepts a hydrogen bond from the NH group of POGLUT1 residue Phe278 (Fig. 3c, d). Given that the C6 atom also makes a stacking interaction with the sidechain of Phe278, it is clear that the hydroxymethyl group of the glucose moiety plays an important role in recognition by POGLUT1. Arg218 and Arg279 both coordinate both phosphates of the donor substrate analog (Fig. 3c), interactions that in many GT-B glycosyltransferases serve to stabilize the negative charge that develops at the transition state during catalysis. As shown by the complex, the only interaction between the donor and the acceptor substrates is the van der Waals contact between the acceptor hydroxyl and C1 of the donor.

The glycosylatable Ser sidechain of EGF(+) in the POGLUT1/EGF(+)/UDP-CH_2_-glucose complex is found to populate two different rotamers, as is also observed in the POGLUT1/EGF(+)/UDP complex. In the gauche (−) rotamer the serine hydroxyl donates a hydrogen bond to the sidechain of the catalytic base, Asp133, and in the gauche (+) rotamer it makes hydrogen bonds to Arg107 and the EGF-like domain C^1^(−1) backbone carbonyl. When hydrogen bonded to Asp133, the serine hydroxyl is positioned for in-line S_*N*_2 attack on C1 of the UDP-CH_2_-glucose analog and the structure provides a model for the Michaelis complex for POGLUT1 glucosylation. In both the POGLUT1/hF7EGF1/UDP and POGLUT1/hEGF12/UDP complexes, the serine hydroxyl is found only in the gauche (−) rotamer where it is hydrogen bonded to the sidechain of the catalytic base and positioned for nucleophilic attack (Fig. 3e–g).

The POGLUT1/EGF(+)/UDP-2F-glucose complex showed that the 2F-glucose moiety had been transferred to EGF(+) in the crystal (Fig. 3h). Bond formation and inversion of stereochemistry at C1 resulted in a rotation of the 2F-glucose moiety relative to that of the glucose moiety in the UDP-CH_2_-glucose complex (Fig. 3i). In this orientation/position, none of the hydrogen bonds to the saccharide hydroxyl groups observed in the UDP-CH_2_-glucose complex are observed. Notably, the orientation/position of the 2F-glucose moiety is very similar to that of the glucose moiety found to be transferred in the dRUMI/hF9EGF1-glucose/UDP product complex (PDB ID: 5F84)^16^. However, the orientation/position of the transferred 2F-glucose moiety is not that observed in the PDB for the glucose moiety of glucosylated EGF-like domains that are not bound by POGLUT1/Rumi (Fig. 3j).

### The donor substrate site accesses two local conformations

**Fig. 4:**
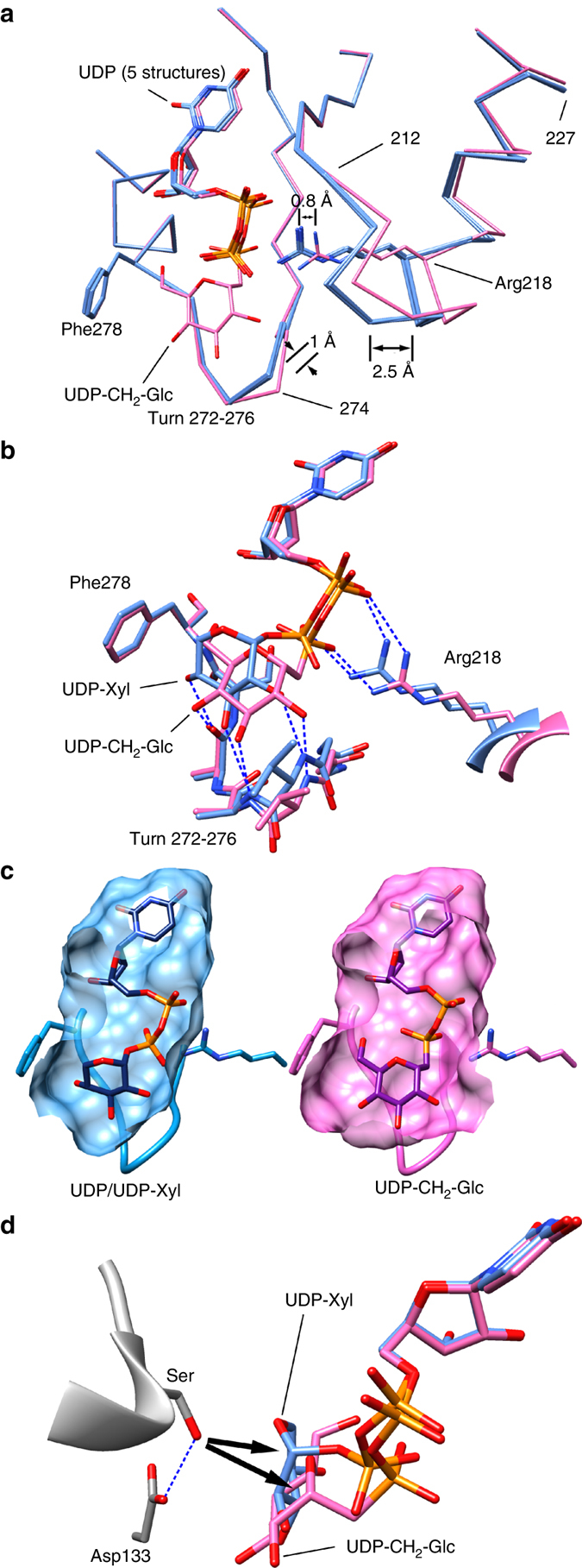
Two local conformational states of the glucose subsite and docking of UDP-xylose. **a** The UDP-CH_2_-glucose complex (*pink*) compared to the five UDP-bound structures (*light blue*). **b** UDP-xylose docked into the UDP conformation (*light blue*), compared to the UDP-CH_2_-glucose complex (*pink*). **c** Comparison of the binding pocket of the UDP bound state (*left*, showing a docked UDP-xylose) and the UDP-CH_2_-glucose bound state (*right*). **d** Superimposition of the docked UDP-xylose (*blue*), the co-crystallized UDP-CH_2_-glucose (*pink*), and the hF7EGF1 complex (showing the acceptor Ser and the catalytic base Asp133). The two *arrowheads* indicate the direction of nucleophilic attack for glucosylation and xylosylation

A comparison of the UDP-CH_2_-glucose complex with that of all of the UDP-containing complexes shows that POGLUT1 can access two local conformational states (Fig. 4a). In the UDP-CH_2_-glucose complex, the 272–276 turn of POGLUT1, which interacts with the glucose moiety, has undergone a segmental shift of up to 1 Å relative to that seen in the UDP complexes. In addition, POGLUT1 residues 212–227, which form a strand-turn-helix, shift up to 2 Å to enable the sidechain of Arg218 to interact with the β-phosphate of the nucleotide sugar. Overall, these conformational changes lead to an increase in the volume of the saccharide subsite of the donor substrate to accommodate the glucose moiety.

In addition to transferring glucose, POGLUT1 can also transfer xylose^14^, a 5-carbon sugar that differs from glucose only by the lack of the hydroxymethyl group of glucose. As discussed above, the hydroxymethyl group is buried and makes key interactions with POGLUT1 in the UDP-CH_2_-glucose complex, interactions that would not be possible with xylose. Building on the observation that POGLUT1 can access two local conformational states, we explored the possibility that POGLUT1 binds UDP-xylose when it is in its UDP-bound conformation. Indeed, with either SwissDock or AutoDock/AutoDock Vina^23–25^, we obtained a compelling model for UDP-xylose binding based on the POGLUT1/hF7EGF1/UDP complex (Fig. 4b, c). The xylose moiety is tilted relative to that observed for the glucose moiety in the POGLUT1/EGF(+)/UDP-CH_2_-glucose complex, but its C2, C3, and C4 hydroxyl groups are hydrogen bonded to the same POGLUT1 groups. The xylose C5 atom, absent the hydroxymethyl group found in glucose, is stacked against POGLUT1 residue Phe278. The C1-O bond of UDP-xylose is well aligned for in-line S_*N*_2-like attack by the serine hydroxyl of the EGF-like domain (Fig. 4d) and Arg218 and Arg279 both coordinate the β-phosphate. Attempts to perform the same docking procedure with UDP-glucose were not successful, an indication that UDP-glucose cannot be accommodated by POGLUT1 when it is in the UDP-bound conformation.

UDP-xylose could also be docked using the POGLUT1/EGF(+)/UDP-CH_2_-glucose complex, although in this case the xylose moiety does not fill the glucose subsite owing to the absence of the hydroxymethyl group (Supplementary Fig. 8b). This UDP-xylose docking model and the one obtained using the POGLUT1/EGF(+)/UDP complex were then subject to all-atom (protein and ligand) energy minimization-repacking calculations using Rosetta^26^. The complex based on the latter showed little change after energy minimization, an indication of the quality of the model obtained when UDP-xylose is docked to POGLUT1 in the UDP-bound conformation. In contrast, the model for UDP-xylose binding based on the POGLUT1/EGF(+)/UDP-CH_2_-glucose complex showed significant local change. The xylose moiety moved toward Phe278 and both the 272–276 turn and the Phe278 sidechain of POGLUT1 moved to interact with the xylose to minimize the cavity created by the loss of the hydroxymethyl group (Supplementary Fig. 8). Taken together, docking and energy minimization supports the suggestion that the POGLUT1 conformation observed in the UDP-containing complexes is responsible for UDP-xylose binding/catalysis. However, further structural analysis will be required to validate that this is the case.

### POGLUT1 catalysis and its serine specificity

**Fig. 5:**
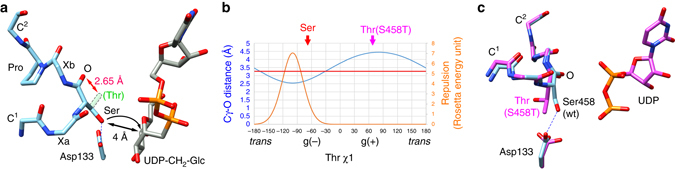
The structural basis for the serine-specificity of POGLUT1/Rumi. **a** Superimposition of hEGF12 and UDP-CH_2_-glucose. The hypothetical position of the γ-methyl group of a Thr residue in the gauche (−) rotamer is indicated by *dotted lines*. The minimum distance for either a Ser or a Thr to the UDP-CH_2_-glucose is 4 Å. **b** Rotamer analysis of Thr at the *O*-glucosylation site. The distances between the γ-methyl group and the backbone carbonyl group are plotted against the *χ*1 angles as the *blue curve*. The minimum non-clashing van der Waals distance between a methyl carbon and a carbonyl oxygen atom (3.25 Å) is indicated by the *red line*. The repulsive energy is plotted as the *orange curve* in Rosetta energy units. The *red* “Ser” indicates the *χ*1 angles found in the wild-type EGF-like domain complexes. The *magenta* “Thr” indicates the *χ*1 angle found in the hEGF12(S458T) mutant complex. **c** The crystal structure of the *O*-glucosylation site mutant (S458T; *magenta*) of hEGF12 superimposed on the wild type structure (*blue*). The minimum distance between the co-crystallized UDP and Thr458 is >6 Å

Unlike other protein-*O*-glycosyltransferases that can glycosylate both serine and threonine, POGLUT1 can only glycosylate serine^3, 15, 27^ and our structures now provide an explanation for why this is the case. POGLUT1 is an inverting glycosyltransferase, a class of enzymes that typically employ an S_*N*_2-like mechanism and possess a catalytic base that activates the nucleophilic hydroxyl group of the acceptor substrate^28^. As shown in Fig. 3e–g, the hydrogen bond to the POGLUT1 catalytic base, Asp133, fixes the serine hydroxyl of the acceptor substrate in the gauche (−) rotamer (*χ*1 = −70°) where it is aligned for nucleophilic attack. Given its main chain conformation (Φ = −90 ± 5°, Ψ = 15 ± 5°), threonine could not access this rotamer owing to the steric clash that would ensue between its sidechain methyl group and its own carbonyl oxygen atom (Fig. 5a). Indeed, the sidechain rotamer energy landscape clearly excludes the gauche (−) rotamer for a threonine residue with these Φ/Ψ angles (Fig. 5b).

To confirm this rationalization, we mutated the *O*-glucosylation site of hEGF12 to a threonine residue (hEGF12:S458T) and measured its ability to be glucosylated and determined its structure in complex with POGLUT1 and UDP. Consistent with the requirement for serine, the threonine mutant was not glucosylated, even at a concentration of 100 μM (Table 1, Supplementary Fig. 9f), and as shown by the structure, the conformation of the *O*-glucosylation motif was essentially unchanged. However, the threonine sidechain was found in the gauche (+) conformation (*χ*1 = 66°) where it hydrogen bonds to Arg107 of POGLUT1 and the carbonyl oxygen atom of the C^1^(−1) residue of hEGF12. In this rotamer, the threonine hydroxyl group is not positioned for in-line nucleophilic attack and its methyl group makes contact with the sidechain of Asp133, an interaction that shifts the Asp carboxyl group (Fig. 5c) relative to that seen in all of the other complexes.

### *O*-xylosylation does not require a diserine-containing motif

**Fig. 6:**
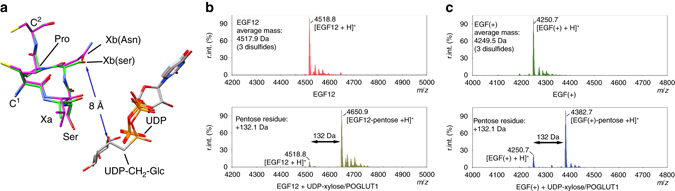
A diserine-containing motif does not determine the donor substrate specificity. **a** Superimposition of the C^1^XSNPC^2^ (hEGF12; *magenta*) and C^1^XSSPC^2^ (hF7EGF1; *green*) motifs and the UDP-CH_2_-glucose complex. The Xb residue that defines a diserine-containing motif is 8 Å away from the glucose moiety. **b**, **c** Deconvoluted ESI-MS spectra showing quantitative in vitro xylosylation of hEGF12 (**b**) and EGF(+) (**c**) by POGLUT1

Previous work had suggested that POGLUT1 required the presence of a diserine-containing glycosylation motif (C^1^XaSSPC^2^, i.e., an additional serine at the Xb position) for efficient xylosylation^14^. Among the EGF-like domains studied here, EGF(+) (C^1^ASNPC^2^) and hEGF12 (C^1^VSNPC^2^) do not possess a diserine-containing motif, while hF7EGF1 (C^1^ASSPC^2^) does. As shown by their UDP complexes, there is little difference between the two EGF-like domain types (Fig. 2b). Moreover, the Xb residue does not make direct or indirect interactions with the donor substrate in either the UDP-CH_2_-glucose complex (Fig. 6a) or our model for UDP-xylose binding. As such, our structures do not provide insight into why a diserine-containing glycosylation motif might be required for xylosylation.

To explore this further, we assayed hEGF12 and EGF(+), substrates lacking diserine, and found that they can both be readily xylosylated (Fig. 6b, c, Supplementary Fig. 10). We also determined the kinetic parameters for hEGF12 and hF7EGF1, for both glucosylation and xylosylation, at saturating concentrations of UDP-glucose and UDP-xylose. A comparison of the *V*_max_ values for all four combinations of donor and acceptor substrates shows that *V*_max_ for the hEGF12/UDP-xylose combination is ~50% lower than that of the other three (Table 1, Supplementary Fig. 9). In addition, we produced the N459S mutant of hEGF12 (C^1^VSSPC^2^), designed to introduce a diserine. The *V*_max_ value of this mutant increased (~2-fold) for the xylosylation reaction, to that observed for the others and it did not change appreciably for the glucosylation reaction. Moreover, the *K*_M_ value of this mutant was ~2-fold lower than that of the wild-type acceptor for the xylosylation reaction. Taken together, these results show that *wt* hEGF12 is a substrate for both glucosylation and xylosylation and that mutating its glucosylation motif to introduce a diserine leads to only a modest ~3-fold increase in *V*_max_/*K*_M_ for the xylosylation reaction. As shown in Table 1, the *K*_M_ values for hF7EGF1 for both glucosylation and xylosylation are considerably lower than that for hEGF12 and the *V*_max_ values for both reactions are similar.

## Discussion

POGLUT1/Rumi recognizes a diverse array of EGF-like domain substrates and can only glucosylate/xylosylate those that are properly folded, properties shared by POFUT1. We now show that for POGLUT1 these properties are a result of an extended shape complementarity as well as interactions with the C^1^C^2^ loop (the site of glucosylation/xylosylation), the C^2^C^3^ loop (the site of POFUT1 fucosylation), and a conserved aromatic residue at the C^4^(−2) position of the EGF-like domain. Backbone atoms are important in the recognition of both loops and, as such, structural/conformational features common to all of its EGF-like domain substrates play key roles in binding/catalysis. Extensive shape complementarity and interactions with backbone atoms of the O-fucosylation motif (i.e., the C^2^C^3^ loop) also characterizes POFUT1’s interactions with its substrates^17^, evidence that the two enzymes recognize their EGF-like domain substrates by similar mechanisms.

Unlike most other protein-*O*-glycosyltransferases, POGLUT1 is specific for acceptor substrates with a serine at the site of glycosylation. Contrary to that previously suggested^16^, we now show that the serine specificity is a result of well-established mainchain–sidechain steric constraints^29, 30^. Given the mainchain conformation of the *O*-glucosylation motif, only serine can access the sidechain rotamer required for activation and in-line nucleophilic attack on C1 of the donor substrate. As such, POGLUT1’s requirement for folded EGF-like domain substrates also leads to its specificity for serine. This is to be contrasted with the large family of polypeptide GalNAc transferases that target flexible or disordered regions of their protein substrates and that can glycosylate either serine or threonine^31^.

Many glycosyltransferases possess loops, often ordered on donor substrate binding, that undergo an order–disorder transition during successive catalytic cycles^32, 33^. These loops have been proposed to prevent donor substrate hydrolysis, to facilitate acceptor binding, to seal the catalytic site from bulk water and following catalysis, to promote product release. Although ordered in all of our structures and the previously reported *apo* form of dRUMI^16^, Loop 1 and Loop 2 of POGLUT1 almost certainly require an order–disorder transition to permit substrate binding and product release. POFUT1 is devoid of such loops and as recently shown^17^, the EGF-like domain interacts with POFUT1 to form a seal around C1 of the donor substrate that excludes bulk water without the involvement of loops that undergo an order–disorder transition. POGLUT1 and POFUT1 recognize different (but overlapping) surfaces on the folded EGF-like domain and these enzymes have clearly evolved a different means of generating the chemical environment required for catalysis. In both cases, however, the acceptor substrate contributes to burying the donor substrate, a stacking arrangement common to glycosyltransferases and that leads to the ordered sequential *Bi-Bi* kinetic mechanism that they typically display.

Although the turnover of a nucleoside diphosphate-2-fluorosugar is not unprecedented^21^, transfer to the acceptor substrate is not typically observed since the inductive effect of the fluorine destabilizes the positively charged transition state sufficiently^28^. The observation that 2F-glucose was transferred to EGF(+) in our POGLUT1/EGF(+)/UDP-2F-glucose crystal suggests that either the POGLUT1 transition state develops little positive charge or that even though it is destabilized, the activation energy for catalysis is still relatively low. The structure also provides insight into the process of product release as it represents a snapshot along the reaction coordinate while the 2F-glucose-EGF(+) product is still bound to POGLUT1. The position/orientation of the transferred saccharide moiety in these complexes is not that observed for the glucose moiety in the glucosylated EGF-like domains found in the PDB. In the latter, the glucose moiety interacts with the highly conserved aromatic residue at the C^4^(−2) position that interacts with POGLUT1 in our complexes. It follows that reorientation of the glucose moiety to interact with the C^4^(−2) residue after transfer may serve as a means of promoting release of the glycosylated EGF-like domain.

Previous work had suggested that POGLUT1’s ability to efficiently xylosylate EGF-like domains was dependent on a diserine-containing glycosylation motif^14^. We have now shown that POGLUT1 can xylosylate EGF(+) and hEGF12, EGF-like domains that contain an Asn at the Xb position. Moreover, we showed that the introduction of a serine residue at the Xb position in hEGF12 leads to only a modest improvement in *V*_max_/*K*_M_ for xylosylation. These results are consistent with the observation that the sidechain of the Xb residue interacts with POGLUT1 and does not interact with the donor substrate. The ability to xylosylate hEGF12, an EGF-like domain possessing the C^1^XaSNPC^2^ motif found in 36% of POGLUT1 substrates (Supplementary Fig. 3), shows that POGLUT1 has the potential to xylosylate many more EGF-like domains than previously thought. The GXylT-mediated and XXylT-mediated elongation of *O*-glucose moieties play important roles in Notch trafficking and signaling^34, 35^. As such, the nature of the roles that the elongation of *O*-xylose might play is an important and open question. Indeed, it has been suggested that the elongation of *O*-xylose on Notch by enzymes involved in GAG synthesis might be possible^14^.

The hydroxymethyl group of the glucose moiety of the donor substrate is buried and makes key hydrogen bonds and van der Waals interactions with POGLUT1. Absent compensatory conformational changes in POGLUT1, the loss of the interactions with the hydroxymethyl group, and the cavity created in its absence, would be expected to destabilize a UDP-xylose complex. Indeed, our structures show that POGLUT1 can assume two local conformational states, the one observed in the UDP-CH_2_-glucose complex and the one observed in all of the UDP-containing complexes. In conjunction with our structural work, docking and energy minimization suggests that binding/reaction of UDP-glucose and UDP-xylose is respectively mediated by these two conformations. We have also shown that *V*_max_/*K*_M_ values for the acceptor substrates assayed here are similar for the two donor substrates. It is certainly possible that access to conformations that are individually optimal for the binding and reaction of each of them are responsible for the comparable catalytic efficiencies observed. In any case, comparable catalytic efficiencies suggest that the extent to which glucose and xylose are transferred to a given acceptor site will depend on the UDP-glucose and UDP-xylose concentrations in the ER^36, 37^.

Proteins such as the Notch receptor possess long tandem repeats of hEGF domains and both the glucosylation and fucosylation of these domains have been found to play roles in their folding, thermal stability and trafficking/secretion^4, 5, 11–13, 35^. The importance of these roles is reflected in the fact that the presence of hEGF domains and POFUT1 has accompanied animal evolution^17^, a correlation that we now show extends to POGLUT1 with few exceptions. We also find that the C^2^C^3^ loop of the hEGF domain, the site of POFUT1 fucosylation, is also recognized by POGLUT1, an observation that may be of significance from an evolutionary standpoint. Among hEGF domains this loop typically contains 5-residues, the optimal length for recognition by both POFUT1 and POGLUT1. The use of a common structural element in this way provides a means of targeting hEGF domains, while minimizing constraints on hEGF domain evolution, and suggests that these enzymes have co-evolved with hEGF domains. Indeed, the shared mechanisms that allow POGLUT1 and POFUT1 to recognize diverse and folded substrates have presumably facilitated the evolution and expansion of hEGF domains and the proteins that contain them.

## Methods

### Expression and purification of proteins

The cDNA sequence encoding residues 29–385 of human POGLUT1 (Supplementary Fig. 1) was cloned into the *piggyBac* transposon-based mammalian expression vector PB-T-PAF^38^. The resulting construct includes an N-terminal human cystatin S secretion signal^39^, a Protein A domain for affinity purification, a TEV cleavage site, and the hPOGLUT1 domain. The Protein A-POGLUT1 fusion protein was secreted from a stably transfected HEK293S GnTI-cell line (obtained from MIT under a MTA)^40^. Cells were scaled-up in DMEM/F12 medium supplemented with 3% (v/v) fetal bovine serum and 1% penicillin-streptomycin. In the production phase, doxycycline was added to a final concentration of 1 μg ml^−1^ to induce the expression of the target protein. The tissue culture medium containing the secreted Protein A–POGLUT1 fusion protein was concentrated 10-fold and purified by IgG Sepharose affinity chromatography (GE Healthcare, cat. #17-0969-01). On-column cleavage was performed using ~0.1 mg ml^−1^ tobacco etch virus (TEV) protease to release the hPOGLUT1 fragment from the IgG beads. The human POGLUT1 fragment expressed contains four *N*-glycosylation sites. The *N*-glycosylated POGLUT1 was deglycosylated by treating the protein with 0.01 mg mL^−1^ EndoH^41^ at 4 °C for 24 h. The deglycosylated POGLUT1 was further purified by ion-exchange chromatography on a HiTrap SP sepharose column (GE Healthcare cat. #17-1151-01) and size-exclusion chromatography on a Superdex 200 10/300 column (GE Healthcare, cat. #17-5175-01). The *Drosophila* Rumi (residues 39–406, containing no *N*-glycans) used for enzymatic assays was produced with the same procedure except for the EndoH treatment. For the affinity chromatography and size-exclusion chromatography, the buffers contained 10 mM HEPES (pH 7.0), 250 mM NaCl. The purified hPOGLUT1 and dRumi were stored in 5 mM HEPES buffer (pH 7.0) containing 250 mM NaCl at 4 °C. The cloning primers are listed in Supplementary Table 2. Protein sequence alignment of hPOGLUT1 with homologs found in representative animal species can be found in Supplementary Fig. 1.

The cDNAs encoding the human Notch1 EGF12 domain and human Factor VII EGF1 domain, and a synthetic sequence, EGF(+), were cloned into the pMAL-p2X vector (New England Biolabs). EGF(+) is a consensus sequence of POFUT1 substrates^17^ and it is also a good POGLUT1 substrate. The mutants of EGF-like domains were generated using the In-Fusion HD Cloning System (Clontech cat. #639647). The protein sequences of the EGF-like domains and their mutants are listed in Supplementary Table 3. The MBP-EGF fusion proteins were produced in the BL21(DE3) strain of *E. coli* by a 3-h induction at 37 °C using 0.25 mM IPTG. The MBP-EGF fusion proteins were then affinity-purified using amylose resin (New England Biolabs, cat. #E8021L). The maltose-eluted MBP-EGF fusion proteins were further purified by ion-exchange chromatography on a HiTrap Q HP column (GE Healthcare cat. #17-1153-01). After TEV cleavage, the free EGF-like domains were separated from the MBP domain by size-exclusion chromatography on a Superdex 75 10/300 GL column (GE Healthcare cat. #17-5174-01) and further purified by ion-exchange chromatography on a HiTrap Q HP column. The free EGF-like domains were then purified by reverse-phase HPLC on a C18 column with an acetonitrile:H_2_O gradient running from 20 to 30% (v/v) in the presence of 0.06% (v/v) trifluoroacetic acid. The EGF-like domains were then concentrated to 2–10 mg mL^−1^ and stored in 5 mM HEPES (pH 7.0), 50 mM NaCl at 4 °C.

### Protein sequence database analysis

The NCBI BLAST non-redundant (nr) protein database contains all known protein sequences and was analyzed using a set of Perl programs, as previously described^17^. To avoid sampling bias only organisms with fully sequenced genomes (listed in the NCBI Taxonomy database) were analyzed. Detection of the O-fucosylation motif was based on a search for the C^2^XXXX(S/T)C^3^ sequence and the detection of the O-glucosylation motif was based on a search for the C^1^XSXPC^2^ motif.

### Crystallization of POGLUT1 complexes

The POGLUT1/EGF complexes with UDP were crystallized using the hanging-drop vapor-diffusion method. Crystals were obtained from 10–20% (v/v) PEG 5000 MME, 50 mM MES pH 6.5, 2–10 mM CaCl_2_, 0–250 mM NaCl, 5–10% (v/v) glycerol or 2-methyl-2,4-pentanediol (MPD), and 1–2 mM UDP. Artificial mother liquors containing 20–25% (v/v) glycerol or 10–20% MPD were used to soak the crystals before freezing in liquid nitrogen. All crystals grew in space group *P*2_1_2_1_2_1_, with one POGLUT1/EGF/UDP complex in the asymmetric unit (Supplementary Table 1). In an attempt to produce a UDP-glucose or UDP-xylose complex, the *O*-glucosylation site serine of hEGF12, hF7EGF1 and EGF(+) were mutated to alanine. In addition, to limit hydrolysis of the donor substrate, a few different measures were taken, including lowering the crystallization temperature to 4 °C, shortening the crystal growth time to 1 day (with seeding), macro seeding followed by the addition of fresh donor substrate and soaking crystals with 2–5 mM fresh donor substrate prior to crystal harvesting. Despite all of these efforts, in all of the resultant structures we only observed the hydrolysis product UDP in the donor substrate binding site.

The slow substrate/inhibitor, UDP-2F-glucose^22^, and the non-hydrolyzable analog, UDP-CH_2_-glucose^21^, were used in co-crystallization experiments with EGF-like domains containing a glycosylatable serine. The use of UDP-2F-glucose resulted in complete transfer of the 2F-glucose moiety to the EGF(+) domain. By contrast, the UDP-CH_2_-glucose was intact in the crystal structure, leading to a donor substrate analog complex with POGLUT1 and EGF(+).

### Structure determination of the POGLUT1 complexes

The structure of the POGLUT1-EGF complex was solved by the single wavelength anomalous dispersion technique using POGLUT1/EGF(+)/UDP crystals grown in the presence of 0.5 M potassium iodide. The diffraction data were collected at 1.77 Å on beamline 08ID-1 at the Canadian Light Source. The data set used for phasing was from a single crystal processed to 2.2 Å resolution. Heavy atom sites were determined using the SHELXD program. Twelve iodide sites were found with occupancy >0.2. The protein phases were calculated using the SHELXE program^42^ with an estimated mean Figure of Merit of 0.654 and pseudo-free CC of 68.59%. Both POGLUT1 and EGF(+) were manually built using the program COOT^43^. All residues except for residue Gly29 of POGLUT1 and the two N-terminal residues (Gly-Ser) of the EGF(+) construct could be built into the initial map. This structure was refined using the programs REFMAC5^44^ and phenix.refine^45^ to an *R*_free_ of 0.21 and an *R*_work_ of 0.18.

The native data sets of the POGLUT1/EGF/UDP complexes were collected at 0.98 Å on beamline 08ID-1 at the Canadian Light Source. The native data sets of the POGLUT1/EGF/UDP-CH_2_-glucose and POGLUT1/EGF-2F-glucose/UDP complexes were collected at 1.00 Å on beamline 17-ID at the Advanced Photon Source (APS). The native data set of the POGLUT1/hEGF12(S458T)/UDP complex was collected at 1.54 Å on a Bruker D8-Venture system equipped with a Cu rotating anode X-ray source. All of the native complexes were solved by molecular replacement using the refined POGLUT1 structure from the POGLUT1/EGF(+)/UDP complex as search model. The EGF-like domains were built manually from 2*mF*o–*DF*c maps. The complexes were built using COOT and refined using REFMAC5 and phenix.refine. The composite omit maps of the EGF-like domains and the UDP or donor analogs can be found in Supplementary Fig. 4. The Dali^46^ search result of hPOGLUT1 can be found in Supplementary Table 4. Structural comparison of hPOGLUT1 with the top Dali search hits can be found in Supplementary Fig. 7.

### Docking of UDP-xylose and UDP-glucose to POGLUT1

The docking of UDP-xylose and UDP-glucose to POGLUT1 was first performed using the SwissDock server (http://www.swissdock.ch/)^23, 47^. POGLUT1 structures in either the UDP-bound conformation or the UDP-CH_2_-glucose-bound conformation were used in the docking searches. The UDP-xylose and UDP-glucose ligands used for docking were supplied by the SwissDock ligand library. As a control, the UDP-CH_2_-glucose molecule was randomized, idealized, and then uploaded to the SwissDock server for docking into the POGLUT1 structures. Using the POGLUT1 structure in the UDP-bound conformation, only UDP-xylose was successfully docked by SwissDock. Using POGLUT1 in the UDP-CH_2_-glucose-bound conformation, UDP-xylose, UDP-glucose and UDP-CH_2_-glucose were all successfully docked.

The docking of UDP-glucose and UDP-xylose to POGLUT1 was then performed again using the AutoDock4 program^24^ and the AutoDock Vina program^25^ to permit protein sidechain flexibility. Protein sidechains surrounding the UDP-glucose binding pocket were treated as flexible. These programs generated results similar to those obtained with the SwissDock server.

### Optimization of the UDP-xylose docking models

The docking solutions were further optimized by re-docking and all-atom repacking calculations using the Rosetta software suite using a previously reported docking protocol^26^. The scoring function settings were not modified from the original protocol. The UDP-xylose docking solutions obtained from SwissDock, AutoDock4, or Autodock Vina were used as the starting point. Both the torsion angles and the overall position of the UDP-xylose molecules were varied in small steps to re-dock them. Then multiple cycles of all-atom repacking and high-resolution docking were carried out before final energy minimization. The backbone and sidechains of all residues within a radius of 10 Å from any atom in the UDP-xylose molecule were allowed to be flexible. The flexible region was extended along the chain by 5 residues (backbone only). Multiple re-docking and repacking solutions were generated from each original docking solution for comparison.

### Enzyme kinetic assays and mass spectrometry

The enzyme kinetic properties of POGLUT1 were assayed using a continuous spectrophotometric assay that couples the generation of UDP with the conversion of NADH to NAD^48^. For the determination of the kinetic properties of the EGF-like domains, each reaction consisted of 50 mM HEPES (pH 7.0), 75 mM NaCl, 75 mM KCl, 20 mM MgCl_2_, 400–1000 μM phosphoenolpyruvate (Sigma-Aldrich, cat. #P7127), 200–500 μM NADH (Sigma-Aldrich, cat. #N8129), 75 unit per ml pyruvate kinase (Sigma-Aldrich, cat. #P7768), 75 unit per ml lactate dehydrogenase (Sigma-Aldrich, cat. #L2500), 2.5 mM UDP-glucose (Sigma-Aldrich, cat. #670120), or 2.5 mM UDP-xylose (CarboSouce Services, Complex Carbohydrate Research Center), an appropriate amount of POGLUT1/dRumi and variable amounts of the EGF-like domains (in the form of MBP-fusion proteins) in a total volume of 50 or 100 μl.

The reactions were carried out in 384-well plates in a temperature-controlled EnSpire® plate reader (PerkinElmer) at 37 °C. The reactions were allowed to proceed for 30–300 min until the EGF-like domain substrates were completely consumed. The UV absorbance of the reactions at 340 nm was constantly monitored to generate reaction progress curves^49^. These curves were then corrected for the rate of NADH oxidation and UDP-glucose hydrolysis obtained from the end-of-reaction region. The corrected reaction progress curves were then fitted to a one-substrate Michaelis–Menten model, assuming that the donor substrate at 2.5 mM concentration saturates the enzyme (*K*_M_ = ~60 μM for both UDP-glucose and UDP-xylose as listed in Table 1). Curve fitting was carried out using the program Dynafit^50^. For each EGF-like domain, multiple reactions starting at various concentrations were fitted globally and the resulting kinetic parameters (*K*_M_ and *V*_max_) were generated (Table 1, Supplementary Fig. 9). For the hEGF12 6-res and 7-res C^2^C^3^ loop mutants we were unable to start the experiments at concentrations close to their expected *K*_M_ values owing to their greatly reduced affinities to POGLUT1/Rumi. Therefore, errors larger than that suggested by the curve fitting statistics are expected and only approximate values, based on the curve fitting results, were reported in Table 1. For the determination of the *K*_M_ values of UDP-glucose or UDP-xylose, varying concentrations of these donor substrates were incubated with POGLUT1 and 50 μM EGF(+). The initial velocities of these reactions were fitted to a Michaelis-Menten model to obtain the *K*_M_ values.

To confirm the xylosylated products, 100 μM EGF(+) or hEGF12 was incubated with 1 mM UDP-xylose and 1 μM POGLUT1 at 37 °C for 3 h. The EGF-like domains were analyzed by LC-MS (C18 reverse phase) using electrospray ionization mass spectrometry (ESI-MS) on a Waters Xevo G2-S QTof instrument. The data were analyzed and deconvoluted using the program mMass^51^.

### Statistical methods

Each *V*_max_ and *K*_M_ value was generated from three or more independent experiments. The number of experiments (*n*), the average values and the standard deviation values are reported in Table 1.

### Code availability

The programs for extracting and analyzing EGF-like domain sequences are available from the authors on request.

### Data Availability

The data supporting the findings of this study are available within the paper and its supplementary information files, or are available from the corresponding author upon request. The structure coordinates have been deposited at the Protein Data Bank with accession codes 5L0R (POGLUT1/UDP/hEGF12), 5L0S (POGLUT1/UDP/hF7EGF1), 5L0T (POGLUT1/UDP/EGF(+)), 5L0U (POGLUT1/UDP-CH_2_-glucose/EGF(+)), 5L0V (POGLUT1/UDP/EGF(+)-2F-glucose), and 5UB5 (POGLUT1/UDP/hEGF12(S458T)).

## Electronic supplementary material

Supplementary Information

Supplementary Data 1

Supplementary Data 2

Peer Review File Reviewer reports and authors' response from the peer review of this Article at Nature Communications
