# Supplementary material for: Structural basis of Notch O-glucosylation and O–xylosylation by mammalian protein–O-glucosyltransferase 1 (POGLUT1)
Source: Nat Commun. 2017 Aug 4;8:185. doi: 10.1038/s41467-017-00255-7 (PMC5543122; doi:10.1038/s41467-017-00255-7)
Supplement: Supplementary file 1 — Supplementary Information [file 41467_2017_255_MOESM1_ESM.pdf]

File Name: Supplementary Information

Description: Supplementary Figures, Supplementary Tables and Supplementary Reference

File Name: Supplementary Dataset 1

Description: This file contains statistics for the distribution of EGF-like domain types (hEGF, cEGF, lamEGF, intEGF) and O-glucosylation motifs in 339 animal species. For each species, the number of EGF-like domains of each type (purple bars) and the number of EGF-like domains that contain the O-glucosylation motif (green bars) are shown in the left panel. The percentages of the EGF-like domains containing the O-glucosylation motif in each type are shown in the right panel (the percentages for the intEGFs are not shown due to the small number of intEGFs in each species). The phyla are indicated in the round brackets. Species lacking a POGLUT1/Rumi homologue sequence are marked with "*No POGLUT1/Rumi" in the EGF counts panels.

File Name: Supplementary Dataset 2

Description: Statistics for the distribution of EGF-like domain types (hEGF, cEGF, lamEGF, intEGF) and O-glucosylation motifs in 339 animal species. These data were used to generate the graphs shown in Supplementary Data 1.

File Name: Peer Review File

Description:

PDB entry	SAD phasing (iodide derivative)	5L0R	5L0S	5L0T	5L0U	5L0V	5UB5
**Acceptor**	EGF(+)	hEGF12	hF7EGF1	EGF(+)	EGF(+)	EGF(+)-2F-Glc	hEGF12:S458T
**Donor**	UDP	UDP	UDP	UDP	UDP-CH_2-Glc	UDP	UDP
**Data collection**							
Space group	$P2_12_12_1$	$P2_12_12_1$	$P2_12_12_1$	$P2_12_12_1$	$P2_12_12_1$	$P2_12_12_1$	$P2_12_12_1$
Cell dimensions							
a, b, c (Å)	69.1 78.4 82.5	70.6 73.6 83.3	70.6 76.9 82.6	71.7 75.2 83.0	71.9 74.4 83.3	70.8 74.9 83.0	71.0 74.1 83.6
α, β, γ (°)	90 90 90	90 90 90	90 90 90	90 90 90	90 90 90	90 90 90	90 90 90
Wavelength (Å)	1.7712	0.9795	0.9795	0.9795	1.0000	1.0000	1.5418
Resolution (Å)[a]	41-2.20(2.28-2.20)	42-1.50(1.55-1.50)	41-1.45(1.50-1.45)	44-1.43(1.48-1.43)	37-1.80(1.86-1.80)	35-1.31(1.35-1.31)	21-2.09(2.16-2.09)
R_{merge}	0.070(0.294)	0.052(0.864)	0.056(0.898)	0.079(1.584)	0.053(0.801)	0.042(0.805)	0.091(0.549)
R_{meas}	0.076(0.334)	0.058(0.976)	0.061(0.996)	0.084(1.700)	0.058(0.868)	0.046(0.875)	0.095(0.608)
I/σ (I)	20.2(4.7)	19.2(1.6)	17.6(1.7)	14.8(1.6)	20.0(2.5)	19.6(2.1)	18.7(3.2)
$CC_{1/2}$	0.998(0.927)	0.999(0.662)	0.999(0.653)	0.998(0.554)	0.999(0.849)	0.999(0.783)	0.998(0.826)
Completeness (%)	99.5(95.6)	98.3(90.5)	99.3(95.3)	100.0(99.7)	90.9(99.8)	96.1(99.8)	99.0(91.5)
Redundancy	6.7(4.5)	5.7(4.7)	6.4(5.3)	8.1(7.7)	6.5(6.7)	6.5(6.4)	11.0(5.3)
**Refinment**							
Resolution (Å)		42-1.50(1.55-1.50)	41-1.45(1.50-1.45)	44-1.43(1.48-1.43)	37-1.80(1.86-1.80)	35-1.31(1.35-1.31)	21-2.09(2.16-2.09)
No. reflections		68848(6230)	79612(7529)	83342(8230)	38232(4135)	103504(10594)	26478(2398)
R_{work}		0.151(0.285)	0.141(0.258)	0.157(0.284)	0.172(0.250)	0.1606(0.261)	0.153(0.233)
R_{free}		0.173(0.290)	0.159(0.253)	0.173(0.307)	0.195(0.260)	0.175(0.277)	0.194(0.280)
Non-hydrogen atoms							
Protein		3288	3289	3266	3268	3250	3293
Ligand/ion		61	81	77	79	101	68
Water		351	377	482	308	582	339
B factors							
Protein		28.8	26.2	27.3	40.0	21.8	29.1
Ligand/ion		22.4	31.4	28.1	49.0	31.4	33.9
Water		37.5	37.7	36.5	42.6	32.8	35.2
r.m.s deviations							
Bond lengths (Å)		0.013	0.009	0.011	0.007	0.011	0.012
Bond angles (°)		1.19	1.01	1.23	0.99	1.27	1.23

Supplementary Table 1. Crystallographic data collection and refinement statistics

[a] Values in parentheses are for the highest-resolution shell.

Primer	Sequence
EGF(+)-fwd	gtacttccagggatccGACATCGATGAATGCGCG
EGF(+)-rev	tgcatgcggccgcttaAATATCATCCTCACAGTTCGG
hEGF12-fwd	ctgtacttccagggatccGACGTTAATGAATGCGTG
hEGF12-rev	tgcatgcggccgcttaGGTGTTTACTTCGCAGT
hF7EGF1-fwd	ctgtacttccagggatccGACGGTGACCAGTGCGCG
hF7EGF1_rev	gttatctatgcggccgcttaTTTGTGGGTTTCGCAGTT
hPOGLUT1_29-fwd	tttatatttccagGGTTCAAAATGGAAAGTATTTA
hPOGLUT1_385-rev	ctatgcggccgcttaGGGAATAATTTGATCATAACCTTT
dRumi_39-fwd	tttatatttccagggcgcgcccGACCAAATAAACGAGGATGA
dRumi_406-rev	gttatctatgcggccgcttaAGGCCCTATGTAAATAAGTT

Supplementary Table 2. Cloning primers used

Sequences in uppercase indicate the template-pairing regions

	Sequence	Notes
**EGF(+)**	GSDIDECASNPCQNGGTCVNTVGSYTCLCPPGFTGPNCEDDI	*wt*
**EGF(+):S7A**	GSDIDECA**A**NPCQNGGTCVNTVGSYTCLCPPGFTGPNCEDDI	O-Glc site mutant. [a]
**hF7EGF1**	GSDGDQCASSPCQNGGSCKDQLQSYICFCLPAFEGRNCETHK	*wt*
**hF7EGF1:S52A**	GSDGDQCA**A**SPCQNGGSCKDQLQSYICFCLPAFEGRNCETHK	O-Glc site mutant. [a]
**hEGF12**	GSDVNECVSNPCQNDATCLDQIGEFQCICMPGYEGVHCEVNT	*wt*
**hEGF12:S458A**	GSDVNECV**A**NPCQNDATCLDQIGEFQCICMPGYEGVHCEVNT	O-Glc site mutant. [a]
**hEGF12:S458T**	GSDVNECV**T**NPCQNDATCLDQIGEFQCICMPGYEGVHCEVNT	O-Glc site mutant
**hEGF12:N459S**	GSDVNECVS**S**PCQNDATCLDQIGEFQCICMPGYEGVHCEVNT	diserine motif
**hEGF12: 4-res C^2C^3**	GSDVNECVSNPCQN_ATCLDQIGEFQCICMPGYEGVHCEVNT	
**hEGF12: 6-res C^2C^3**	GSDVNECVSNPCQND**G**ATCLDQIGEFQCICMPGYEGVHCEVNT	
**hEGF12: 7-res C^2C^3**	GSDVNECVSNPCQND**GG**ATCLDQIGEFQCICMPGYEGVHCEVNT	

Supplementary Table 3. EGF-like domains and mutants used [b,c]

[a] O-glucose site Ser to Ala mutants used for co-crystallization with UDP-Glc/UDP-Xyl. The resulting structures showed only UDP in the donor binding site and were not reported.

[b] In all cases an additional Gly and Ser residue resulting from the BamHI restriction site (GGATCC) is present on the N-terminus of each EGF-like domain.

[c] Underscore, the O-glucosylation site; in bold font, sites of mutation.

Dali #	Chain	Z	rmsd	%id	Donor	Link-age	Acceptor	Mech-anism	Enzyme
**1**	5f85-A	52	1	58	UDP-Glc	β-	EGF-like domain	inverting	Rumi, the *Drosophila* homolog of POGLUT1
**10**	4xsu-A	11.6	3.8	8	UDP-Glc		mannose		Anabaena Alr3699/HepE, a glucosyltransferase involved in the formation of the heterocyst envelope polysaccharide in cyanobacteria.
**15**	5i45-A	11.4	3.1	6					C-terminal domain of Glycosyl Transferase Group 1 family protein (LpcC) from *Francisella tularensis*
**18**	1ya6-A	11.3	4.6	11	UDP-Glc	α-	5-hydroxy-methylcytosine in DNA	retaining	Bacteriophage T4 α-glucosyltransferase
**19**	3mbo-D	11.1	4.1	8	UDP-GlcNAc	α-	L-malate	retaining	Glycosyltransferase BaBshA
**20**	2bgt-A	11.1	4.4	8	UDP-Glc	β-	5-hydroxy-methylcytosine in DNA	inverting	Bacteriophage T4 β-glucosyltransferase
**26**	2jjm-C	10.9	4.2	9					A GT4 glycosyltransferase from *Bacillus anthracis* ORF BA1558
**39**	3okp-A	10.8	3.9	10	GDP-Man	α1,6	phosphatidyl-myo-inositol	retaining	*Corynebacterium glutamicum* PimB
**49**	4x7m-B	10.7	4.5	9	UDP-GlcNAc	α1,4	wall teichoic acid	retaining	*Staphylococcus aureus* TarM
**57**	3fro-C	10.6	3.5	6	UDP-Glc	α1,4	glycogen	retaining	*Pyrococcus abyssi* glycogen synthase
**59**	2nzx-B	10.6	4.1	7	GDP-Fuc	α1,3	Gal-β-1-4-GlcNAc-β-O-R	inverting	*Helicobacter pylori* α1,3-Fucosyltransferase
**65**	1rzu-B	10.5	3.4	5	UDP-Glc	α1,4	glycogen	retaining	Glycogen synthase from *Agrobacterium tumefaciens*
**100**	2f9f-A	10.3	3.1	9					Putative mannosyl transferase (wbaZ-1) from *Archaeoglobus fulgidus*
**106**	3c4v-A	10.3	4.1	8	UDP-GlcNAc	α1,3	1-L-myo-inositol-1-phosphate	retaining	*Corynebacterium glutamicum* MshA
**109**	2gek-A	10.2	4.9	10	GDP-Man	α1,2	phosphatidyl-myo-inositol	retaining	*Mycobacterium smegmatis* phosphatidylinositol mannosyltransferase PimA
**116**	2x6r-A	10.1	4	8	UDP-Glc	α,α1,1		retaining	*Pyrococcus Horikoshii* trehalose synthase
**120**	3oy7-A	10	4.1	13	GDP-Man				Gene product B736L from chlorovirus NY-2A

Supplementary Table 4. Dali search results for POGLUT1

Redundant entries were removed. Entries with Z ≥ 10.00 are shown. The donor/acceptor/linkage/mechanism of these enzymes are based on the related literature. See **Supplementary Fig. 7** for structural overlays.

Phylum	Species	Presence of POFUT1	Life style
Nematoda	*Ancylostoma ceylanicum*	not detected	parasitic
Nematoda	*Ancylostoma duodenale*	POFUT1 homologue<br>E value = 6e-16	parasitic
Nematoda	*Caenorhabditis brenneri*		
Nematoda	*Caenorhabditis briggsae*		necromenic?
Nematoda	*Caenorhabditis elegans*		phoretic (facultative necromentic?)
Nematoda	*Caenorhabditis remanei*		phoretic (facultative necromentic?)
Nematoda	*Dictyocaulus viviparus*		parasitic
Nematoda	*Haemonchus contortus*		parasitic
Nematoda	*Loa loa*		parasitic
Nematoda	*Necator americanus*		parasitic
Nematoda	*Oesophagostomum dentatum*	not detected	parasitic
Nematoda	*Pristionchus pacificus*	not detected	necromenic
Nematoda	*Strongyloides ratti*		parasitic
Platyhelminthes	*Clonorchis sinensis*	not detected	parasitic
Platyhelminthes	*Echinococcus granulosus*	not detected	parasitic
Platyhelminthes	*Echinococcus multilocularis*	not detected	parasitic
Platyhelminthes	*Hymenolepis microstoma*	not detected	parasitic
Platyhelminthes	*Opisthorchis viverrini*	not detected	parasitic
Platyhelminthes	*Schistosoma haematobium*		parasitic
Platyhelminthes	*Schistosoma japonicum*	POFUT1 homologue<br>E value=3e-14	parasitic
Platyhelminthes	*Schistosoma mansoni*		parasitic
Cnidaria/myxozoa	*Thelohanellus kitauei*	not detected	parasitic

Supplementary Table 5. The 22 species with no POGLUT1/Rumi sequence [a,b]

[a] Shown are the 22 species without a significant (BLAST E value < 1e-20) POGLUT1/Rumi homologue. The NCBI BLAST non-redundant (nr) protein database was used. All 339 genome-sequenced animal species were analyzed. See **Supplementary Data Set 1** for the O-glucosylation motif frequencies in all 339 species.

[b] In the "Presence of POFUT1" column, blank cells indicate that a POFUT1 homologue (with protein BLAST E value < 1e-20) exists, as reported [1]. All 9 species that were found to lack POFUT1 sequence ("not detected") are among the 22 species without POGLUT1/Rumi.

	6-Cys EGFs	hEGFs	Fuc	Glc	(Glc&Fuc)	Glc_hEGF	(Glc&Fuc) /Glc	Glc_hEGF /Glc
339 animals	316904	156184	66179	40598	20930	38064	51.6%	93.8%
Hs	1258	652	296	166	89	153	53.6%	92.2%
Ci	1491	596	384	167	71	140	42.5%	83.8%
Sp	3751	2995	1665	1527	822	1502	53.8%	98.4%
Ob	1012	581	248	164	76	159	46.3%	97.0%
Dm	777	249	104	47	24	46	51.1%	97.9%
Ep	1518	994	465	188	108	188	57.4%	100.0%
Aq	1008	388	182	62	44	62	71.0%	100.0%
Ta	1038	763	363	398	194	395	48.7%	99.2%

Supplementary Table 6. Correlation of O-glucosylation motifs with O-fucosylation motifs and with hEGFs

"(Glc&Fuc)/Glc", percentages of O-glucosylation motif containing domains that also contain the O-fucosylation motif. "Glc_hEGF/Glc", percentages of O-glucosylation motif containing domains that are hEGF domains. The other columns list the EGF-like domain counts as follows: all 6-Cys EGF-like domains ("6-Cys EGFs"), hEGF-type domains ("hEGFs"), O-fucosylation motif containing domains ("Fuc"), O-glucosylation motif containing domains ("Glc"), domains containing both motifs "(Glc&Fuc)", hEGF domains that contain the O-glucosylation motif ("Glc_hEGF"). 339 animal species were analyzed. A few representative species are listed below the totals for all 339 animal species: *Hs*, *Homo sapiens*; *Ci*, *Ciona intestinalis* (vase tunicate); *Sp*, *Strongylocentrotus purpuratus* (purple sea urchin); *Ob, Octopus bimaculoides* (octopus); *Dm, Drosophila melanogaster*; *Ep, Exaiptasia pallida* (Sea anemone; *Cnidaria*); *Aq, Amphimedon queenslandica* (sponge); *Ta, Trichoplax adhaerens* (a basal animal; *Placozoa*).

```
                                                                 29
Hs    1 ------MEWW ASSPLRLWLL LFLLPSAQ-- ---------- ------GRQK ESGSKWKVFI DQINRSLENY  46
Ci    1 ------MLHL HSKHYKISAL HTVLFVSV-- ---------- -IWANLSRAS AKEPNWQVYL DAISEAAANY  51
Sp    1 ---MSASCRT KMGMARSGIL RFCLLLT--- ---------C SQLLFTQADT ENDEKWQPYL SLIEEATSSY  55
Ob    1 ---MATKFYL SSCLLLSTVL TLVLASQPDT CQTDGSCDEK PSSDHEKSKY STGNKWQKYL TLIEKANKNY  67
Dm    1 ------MLIN HLIVVLLISL VGTGGAEDD- --GLCSADQK SCAQSEPDQI NEDEFSFKIR RQIEKANADY  61
Ep    1 MNFLLLTLYS LSLFKNIISL EKDQNCDR-- -----ENCED DRVNHEKEKY KLKASWKGYL DNINSAVAKF  63
Aq    1 ------MLQY LLLFLLLTAV HCT------- ---------- -----AEPST RNSSKWSHYI QQYENAISLQ  42
Ta    1 ---------- ---------- ---------- ---------- ---------K ADNDKWDDYL KIITKAVSSF  21

Hs   47 ----EPCSSQ NCSCYHGVIE EDLTPFR--- GGISRKMMAE VVRR------ KLGTHYQITK NRLYRENDCM 103
Ci   52 ----TSCGGN -CTCHSDVIT SDLKLWR-ER GGITNEDMKR GLE------- -RSVHYQIID HKLYRQDKCM 107
Sp   56 ----EACVTD DCSCHAGVMD EDLSVWE-ER GGITRADVVK AES------- -RGTLYQVIN HRLYREEKCM 112
Ob   68 ----KSCKQT NCSCHIQVLD DDLHVWEHEK KGISRAEMQA AKD------- -KGVYYQIIK NKLYREKECM 125
Dm   62 KPCSSDPQDS DCSCHANVLK RDLAPYK--S TGVTRQMIES SAR------- -YGTKYKIYG HRLYRDANCM 121
Ep   64 ----HDCSNK KCGCYSNVID QDLQLWR-DR GGITKHDFTA ASG------- -KGTHYQIIN HKLYRQEDCM 120
Aq   43 G--NTSHTYT CGGEYMGVLE SDLGVWR-ER GGIKREEFIH AKS------- -KGVHYQIVN GKLYREKDCL 101
Ta   22 ----KDCKND RCGCYKKVID NDLKPWR-LK KGITKEIFDK AANQGSHRGA EKGSHYQIIN HKVYRHERCT  86
     Phe104                       Asp133
Hs  104 FPSRCSGVEH FILEVIGRLP DMEMVINVRD YPQVPKWME- -PAIPVFSFS KTSEY-HDIM YPAWTFWEGG 170
Ci  108 FPSRCSGIEH FILEIINDLP DMELGINVHD WPQVMKHSP- -YPMPILSFS KVAKEHQDIM YPAWTFWAGG 175
Sp  113 FPARCNGVEH FILRIIKKLP DMEFVMNVRD WPQSGKYT-- -DPIPVLSFS KVQSQHYDIM YPAWTFWEGG 179
Ob  126 FPFRCSGVEH FIKKLLDDLP DMEMVINVHD YPRVQKFL-- -DPLPVFSFS KVVSDFWDIM YPAWTFWEGG 192
Dm  122 FPARCEGIEH FLLPLVATLP DMDLIINTRD YPQLNAAWGN AAGGPVFSFS KTKEY-RDIM YPAWTFWAGG 190
Ep  121 FLFRCKGVEH FILEIIDKLP NMEMIINVRD WPQVPVWG-- -EPLPVFSFS KTSNE-RDIM YPAWTFWEGG 186
Aq  102 FSFRCKGVEH FILNIIEDLP NMELIINVFD YPKSHKYH-- -SPLPVFSFS KTVHY-WDIM YPAWTFWSGG 167
Ta   87 FPARCKGIEH FLKKIAKKLP NLELIINTHD WPKVPKWD-- -ELLPVFSFS KTHNE-NDIM YPAWSFWEGG 152
                                                          Arg218               loop
         loop170-181                                                         238-242
Hs  171 PAVWPIYPTG LGRWDLFRED LVRSAAQWPW KKKNSTAYFR GSRTSPERDP LILLSRKNPK LVDAEYTKNQ 240
Ci  176 PAVWPIYRNG LGRWDLMRKD LKKKDNEFPW EKKQNIGFFR GSRTSSERDP LVLLSRENPD LVDAQYTKNQ 245
Sp  180 PAVWPLFPTG LGRWDLFRES IDKESQKLPW DTKEDKAFFR GSRTTAERDP LVLLSRDDPD LVDASYTKNQ 249
Ob  193 PAVWPIYPTG LGRWDQQRKI ISQKAAEFPW DKKISKGFFR GSRTSSERDP LILLSRKSPN LVDAEYTKNQ 262
Dm  191 PATK-LHPRG IGRWDQMREK LEKRAAAIPW SQKRSLGFFR GSRTSDERDS LILLSRRNPE LVEAQYTKNQ 259
Ep  187 PAVWPIYPTG LGRWDLMRDA LIKKSQEFPW EKKAPIAFFR GSRTSHERDP LVLLSRSNPE LADAKYTKNQ 256
Aq  168 PAVS-VEPTG LGRWDLKRIS ITKSAKQWPW DKKKSLLFFR GSRTSSERDS LILLSRDKPH LVDAAYTKNQ 236
Ta  153 PAVWPIFPNG LGRWDVLRKS LQKASDKWPW DKKKSIAFFR GSRTSAERDP LILLSRAKPK LVNASYTKNQ 222
          Trp174                      272-278 Arg279                          Gln240
Hs  241 AWKSMKDTLG KPAAKDVHLV DHCKYKYLFN FRGVAASFRF KHLFLCGSLV FHVGDEWLEF FYPQLKPWVH 310
Ci  246 AWKSKKDTLG AEPAEIVHLL DHCQYKYLFN FRGVAASFRL KHLFLCGSLV FHVGEDWLEF FYPALKPWVH 315
Sp  250 AWKSDADTLH MPPAKEMTLE DHCKYRYLFN FRGVAASFRL KHLFLCRSLV FHVGDEWLEF FYPALKPWVH 319
Ob  263 AWKSMEDTLG KKPAEEVKLE DHCKYKYLFN FRGVAASFRL KHLFLCDSLV FHVGSDWLEF FYPSLKPWVH 332
Dm  260 GWKSPKDTLD APAADEVSFE DHCKYKYLFN FRGVAASFRL KHLFLCKSLV FHVGDEWQEF FYDQLKPWVH 329
Ep  257 AWKSDKDTLY AEPAKEVPLE DHCQFRYLFN FRGVAASFRF KHLFLCKSLV FHVGEEWLEF FYPALKPWVH 326
Aq  237 AWRSSKDTLN APPADEVKLE EHCQYKYLVN FRGVAASFRF KHLFLCHSVV FHVGKEWIEF FYPALKPWIH 306
Ta  223 AWRSKADTLG EEPAKEVTLE DHCKYKYLFN FRGVAASFRF RHLFLCNSVV LHIGHEWQEF FYPALTPWVH 292

Hs  311 YIPVK--TDL SNVQELLQFV KANDDVAQEI AERGSQFIRN HLQMDDITCY WENLLSEYSK FLSYNV-TRR 377
Ci  316 YIPVS--PSL NEVKDLINFA KENDEVVKKI ANRGKRFITK HLKMDDISCY WKKLLFQYAE LLKYKVGRRM 383
Sp  320 YIPVK--QDL SDARELIEFA KANQEVAQQV ADRGRDFIWN HLRMDDVQCY WKDLLKRYAK LQKYKP-KHR 386
Ob  333 YIPVP--NDM SNVRELLEFA QENDDTVKKI ADRGRKHIWN HLQMKDITCY WKTLLQRYSR LFNYKP-HFN 399
Dm  330 YVPLKSYPSQ QEYEHILSFF KKNDALAQEI AQRGYDFIWE HLRMKDIKCY WRKLLKRYVK LLQYEV-KPE 398
Ep  327 YIPVK--TDL SDARDLIEFA NANDEVAQGI ASRGYDFIKD HLRMQDIRCY WKKLLKNYAK LLKWKP-VRN 393
Aq  307 YVPLT--TDT VDIQDMIDFV KDNDDIAKSI AVRGFEFVWN NLRPEDVECY WKRLLIEYSK LLLWTV-DKE 373
Ta  293 YIPVD--PDQ RNTEEIIRFA IENDEEMKRL AKRGRDFILN HLRMKDVECY WELLLKQYAK LLKWKP-QLN 359
                385
Hs  378 KGYDQIIPKM LKTEL 392
Ci  384 EYQEIVSPHK HKSEL 398
Sp  387 KDLQEIKPKK DEL-- 399
Ob  400 KDLILIK--- ----- 406
Dm  399 DQLIYIGPKK DEL-- 411
Ep  394 NKLIQIHPEK KSS-- 406
Aq  374 RDYSLIS--- ----- 380
Ta  360 PSFDLVS--- ----- 366
```

Supplementary Figure 1. Sequence alignment of POGLUT1/Rumi

POGLUT1/Rumi sequences of representative species are shown. Selected residues discussed in the main text are indicated by thick arrowheads and brackets. The hPOGLUT1 construct used in this study included residues 29-385, as indicated by the two arrows above the alignment. *Hs*, *Homo sapiens*; *Ci*, *Ciona intestinalis* (vase tunicate); *Sp*, *Strongylocentrotus purpuratus* (purple sea urchin); *Ob, Octopus bimaculoides* (octopus); *Dm, Drosophila melanogaster* (fruit fly); *Ep, Exaiptasia pallida* (sea anemone; *Cnidaria*); *Aq, Amphimedon queenslandica* (sponge); *Ta, Trichoplax adhaerens* (a basal animal; *Placozoa*).

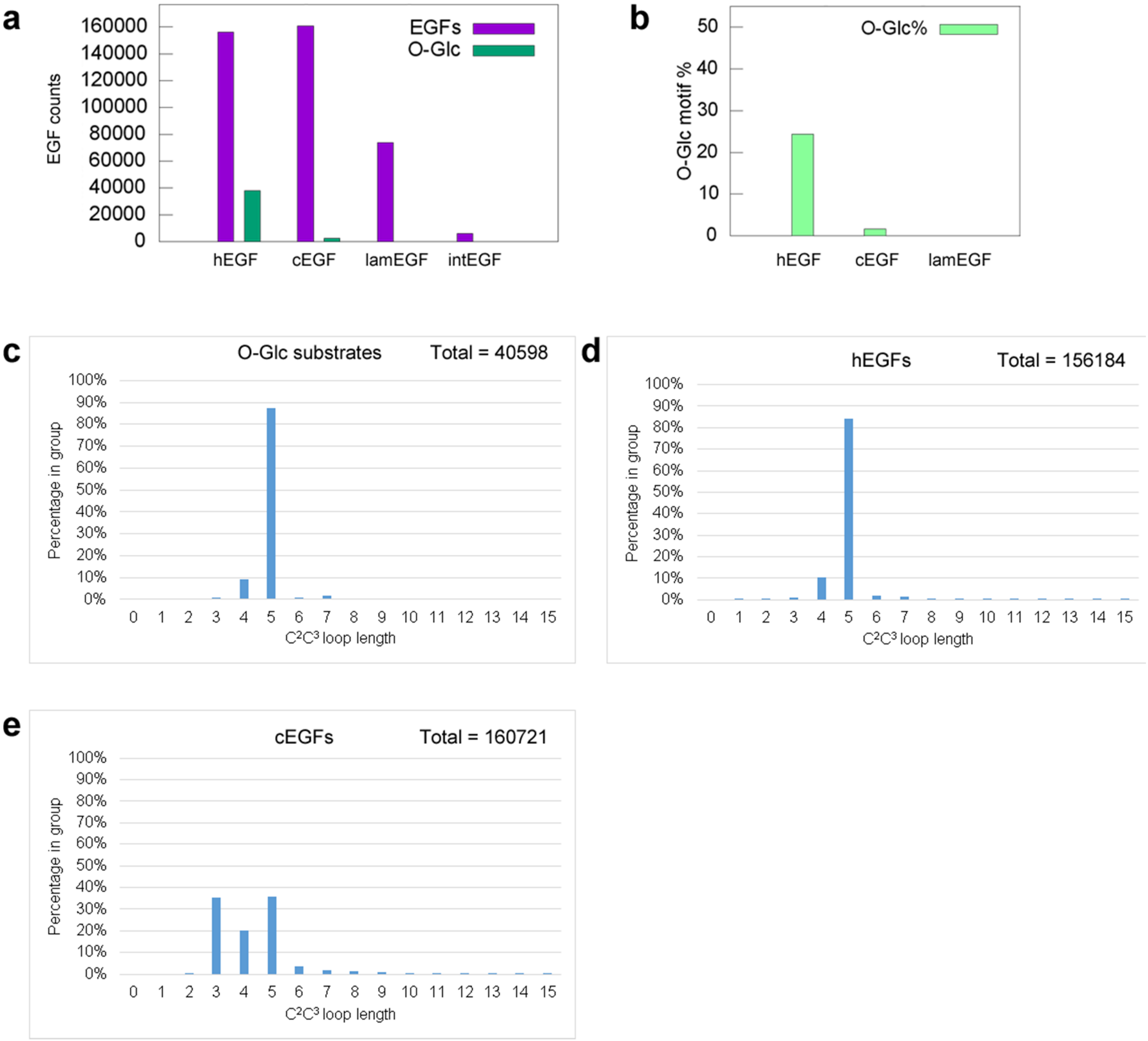

Supplementary Figure 2. Correlation of the O-glucosylation motif, hEGFs, and the length of the C^2C^3 loop

All statistics shown were generated from 339 animal species. (**a**) Number of EGF-like domains ("EGFs") and number of O-glucosylation motifs ("O-Glc") found in the four types of EGF-like domains. (**b**) Frequencies of the O-glucosylation motif found in hEGFs, cEGFs and lamEGFs. There are very few intEGFs in each species and the statistics are not shown. (**c**) The C^2C^3 loop length distribution found in O-glucosylation motif-containing domains. (**d,e**) The C^2C^3 loop length distributions in the hEGFs and cEGFs, respectively. hEGFs and cEGFs each account for approximately half of the 6-Cys EGF-like domains. lamEGFs and intEGFs are 8-Cys EGF-like domains.

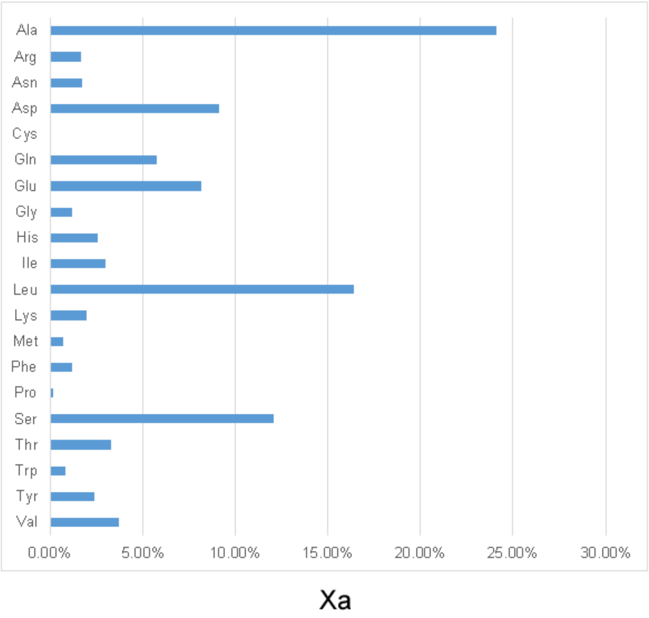

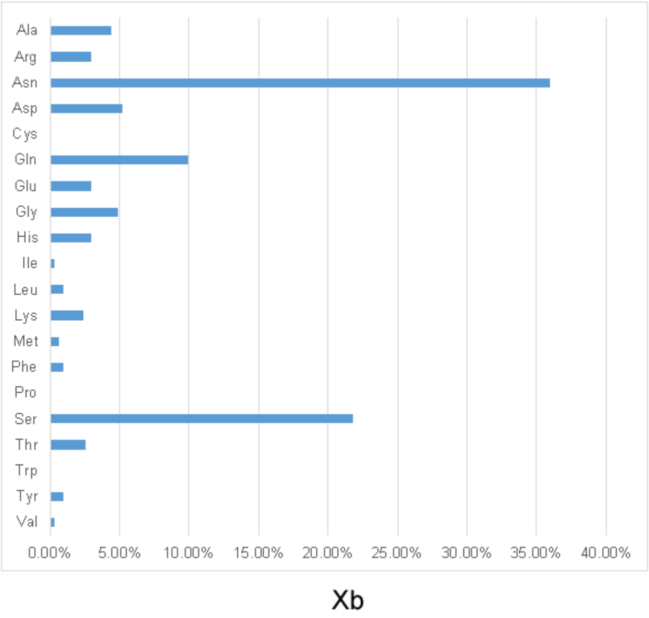

Supplementary Figure 3. Frequencies of amino acids at the Xa and Xb positions in the O-glucosylation motif

Frequencies are computed from ~40,600 O-glucosylation motif ($C^1XaSXbPC^2$) containing EGF-like domains found in 339 animal species. Xb = Ser defines the diserine motif. In the 3 co-crystallized EGF-like domains, Xa = Ala/Val, Xb = Asn/Ser.

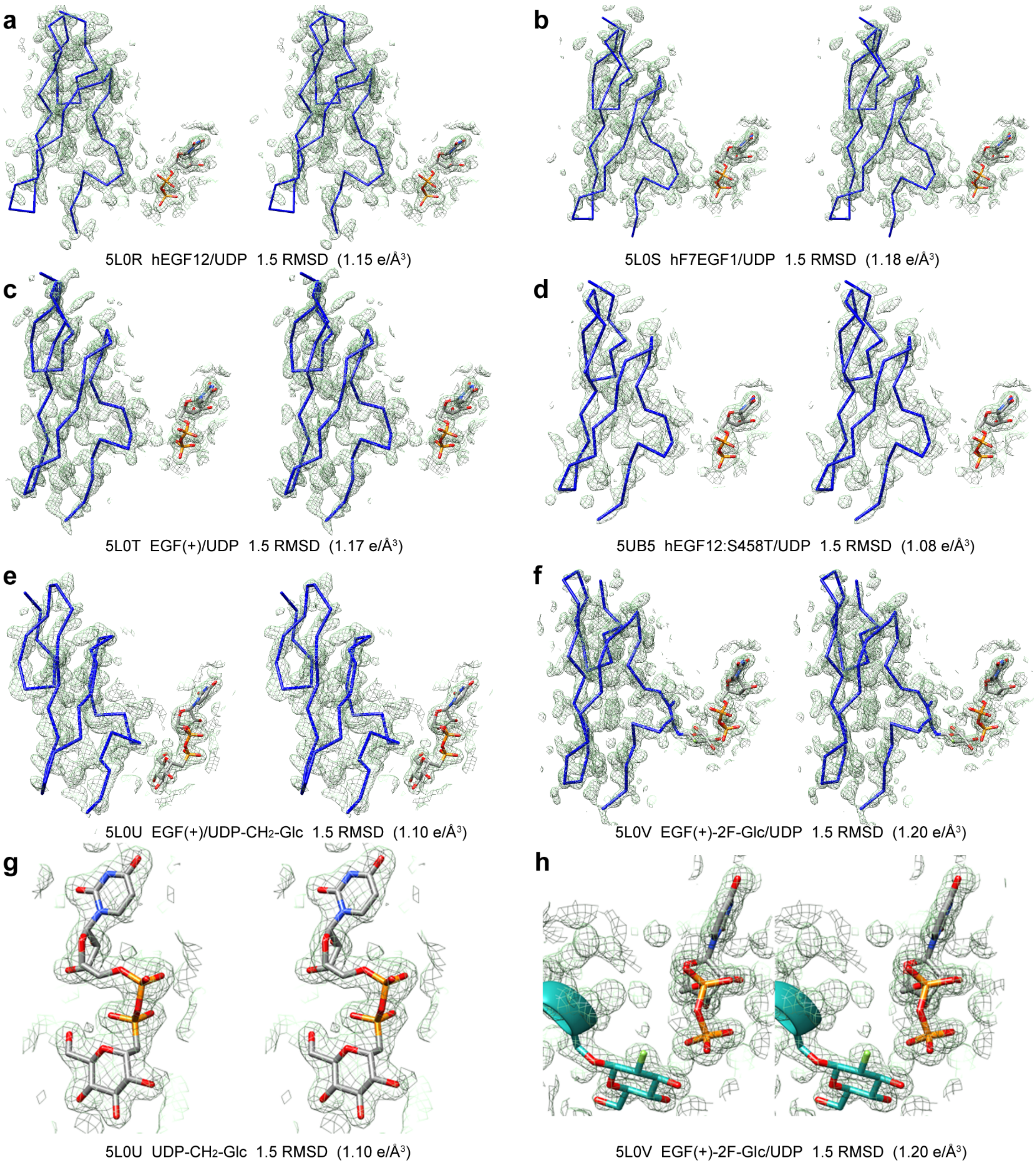

Supplementary Figure 4. Composite-omit maps of the EGF-like domains and the co-crystallized UDP or donor analogs found in the six POGLUT1 complexes

(**a**–**f**) The EGF-like domains and the UDP or donor analogues in the six reported complexes. (**g**, **h**) The UDP-CH_2-glucose and the 2F-glucose/UDP. The composite omit maps (2*mF*o – *DF*c) are contoured at 1.5 RMSD. Each panel shows a wall-eye stereo view. For clarity, a 3 Å radius electron density mask was applied.

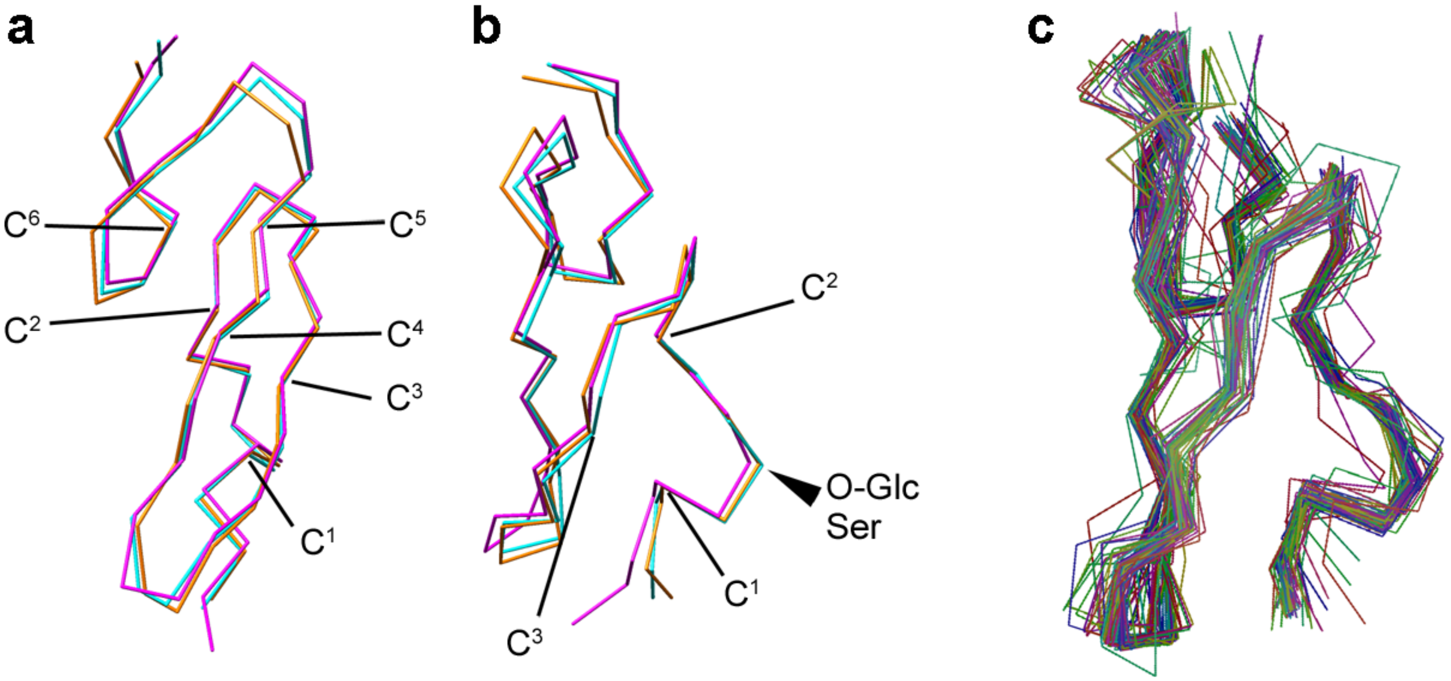

Supplementary Figure 5. Comparison of the 3 co-crystallized EGF-like domains and POGLUT1 substrates in the PDB

(**a,b**) The 3 co-crystallized EGF-like domains. The structural alignments were generated based on all Cα atoms of the hPOGLUT1 molecule. The Cα traces of the 3 EGF-like domains are shown in two views. Magenta, EGF(+); Cyan, hF7EGF1; Orange, hEGF12. Pairwise Cα RMSD: hEGF12–hF7EGF1, 0.77 Å; hEGF12–EGF(+), 1.16 Å; hF7EGF1–EGF(+), 0.82 Å; (**c**) Overlay of 82 structures of the 14 unique EGF-like domains in the PDB that contain the O-glucosylation motif.

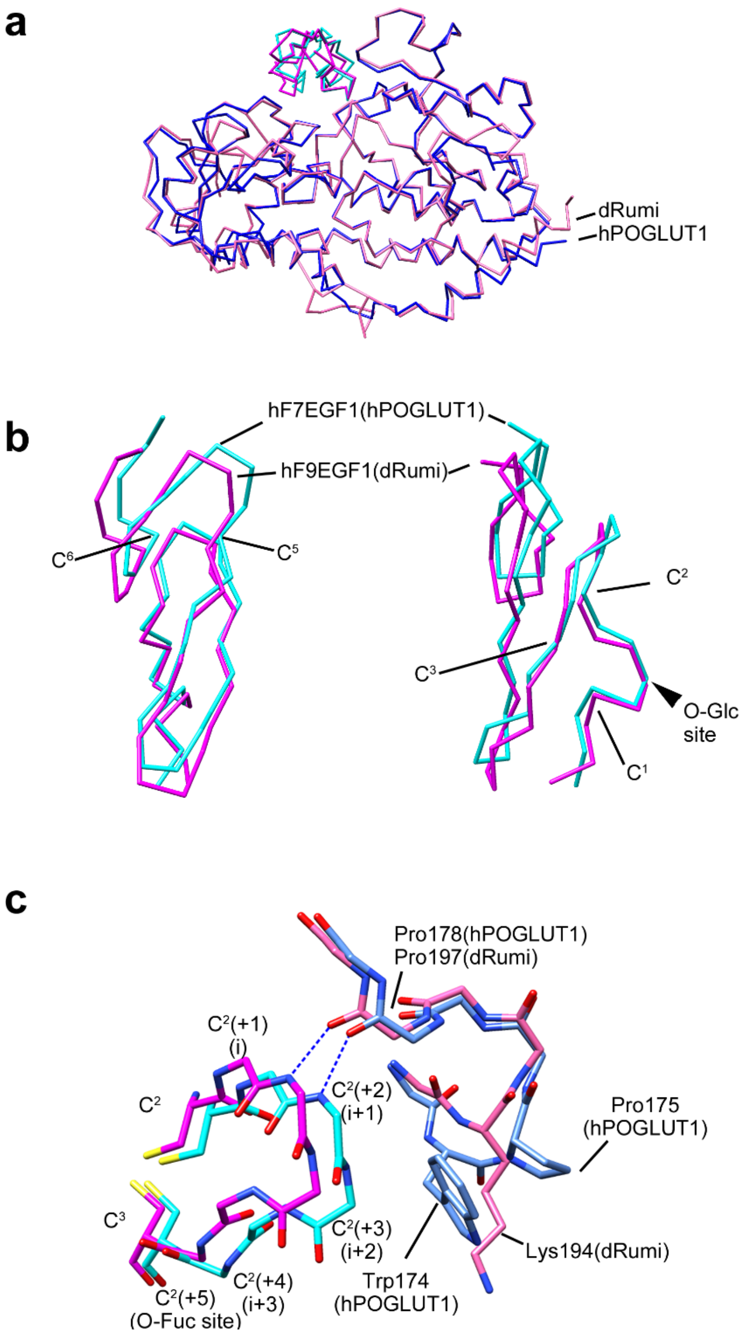

Supplementary Figure 6. Comparison between the hPOGLUT1/hF7EGF1/UDP complex and the dRumi/hF9EGF1/UDP complex

(**a**) Overlay of the hPOGLUT1/hF7EGF1/UDP complex with the dRumi/hF9EGF1/UDP complex (PDB: 5F85), showing the Cα traces. Pink, dRumi; blue, hPOGLUT1; magenta, hF9EGF1; cyan, hF7EGF1. The overlay was generated by aligning the two enzymes (Cα RMSD = 0.68 Å for 316 pruned Cα pairs, or 1.80 Å for all 352 Cα pairs). (**b**) Two views of the hF7EGF1 and hF9EGF1 in the overlay shown in (**a**). The differences between the backbone traces mainly result from a small rotation of the EGF-like domain in the dRumi complex towards the C^6(-1)/C^6(-2) residues. The largest backbone deviations are found in the C^5C^6 region (left panel). The backbone traces of the C^1C^2 loops are very similar (right panel). The Cα RMSD between the two EGF-like domains is 2.10Å. (**c**) The hPOGLUT1 Trp174/Pro175 residues that stack with the β-turn of the C^2C^3 loop (residues i, i+1, i+2, i+3) are replaced by a single Lys194 residue in dRumi. With exception of *Ecdysozoa* (nematodes and arthropods) and sponges, POGLUT1/Rumi contain the Trp-Pro sequence at this position (see **Supplementary Fig. 1** for sequence alignment). Cyan/blue, hF7EGF1/hPOGLUT1 complex; magenta/pink, hF9EGF1/dRumi complex.

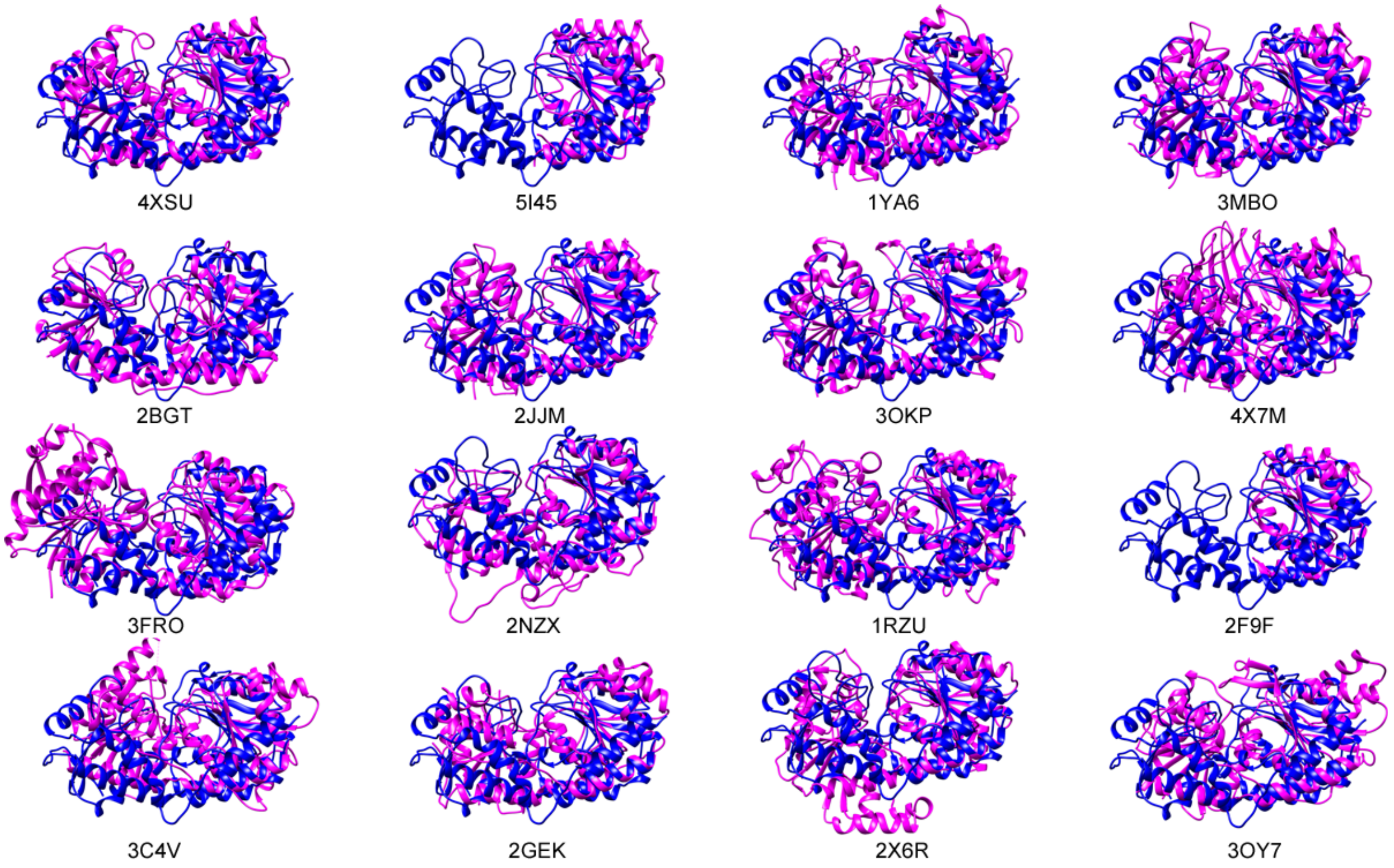

Supplementary Figure 7. Overlay of hPOGLUT1 with the top Dali search results

In all panels hPOGLUT1 is shown in blue and the overlaid protein is shown in magenta. These enzymes carry out diverse functions, utilize a few different donor substrates and employ both inverting and retaining catalytic mechanisms (see **Supplementary Table 4**).

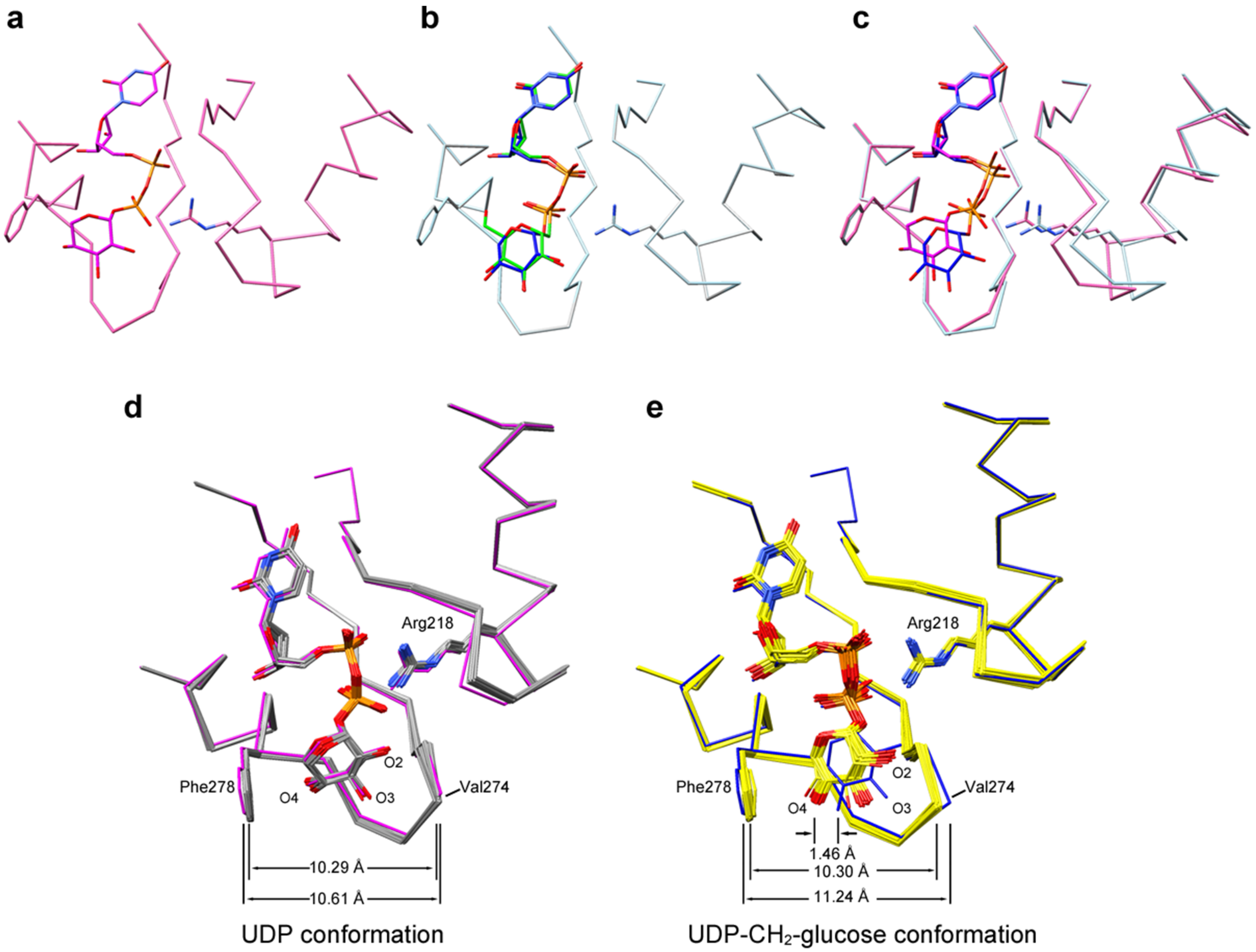

Supplementary Figure 8. Docking and optimization of UDP-xylose binding to POGLUT1

(**a**) UDP-xylose (magenta) docked into the UDP-conformation of hPOGLUT1 (pink). (**b**) UDP-xylose (blue) docked into the UDP-CH_2-glucose-conformation of hPOGLUT1 (light blue). The crystal structure of UDP-CH_2-glucose (green) is superimposed to show the cavity created by the missing hydroxymethyl groups in the docked UDP-xylose. (**c**) Superimposition of UDP-xylose docked in the two POGLUT1 conformations; colored as in (**a**) and (**b**). (**d,e**) Rosetta optimization of the complexes shown in (**a**) and (**b**), respectively. The UDP-xylose was perturbed and re-docked, followed by repacking and energy-minimization. 50 resulting models (gray and yellow, respectively) and the starting docking models (magenta and blue, as in (**a**) and (**b**), respectively) are shown. The width of the saccharide subsite in (**d**) and (**e**) are measured by the distance between the centroid of the Phe278 benzene rings and the centroid of the Cα atoms of residue Val274. In (**e**) the distance between the centroid of O4 in the 50 optimization results and the original position of O4 in the docking model is also indicated. The equivalent distance in (**d**) to that is 0.33 Å.

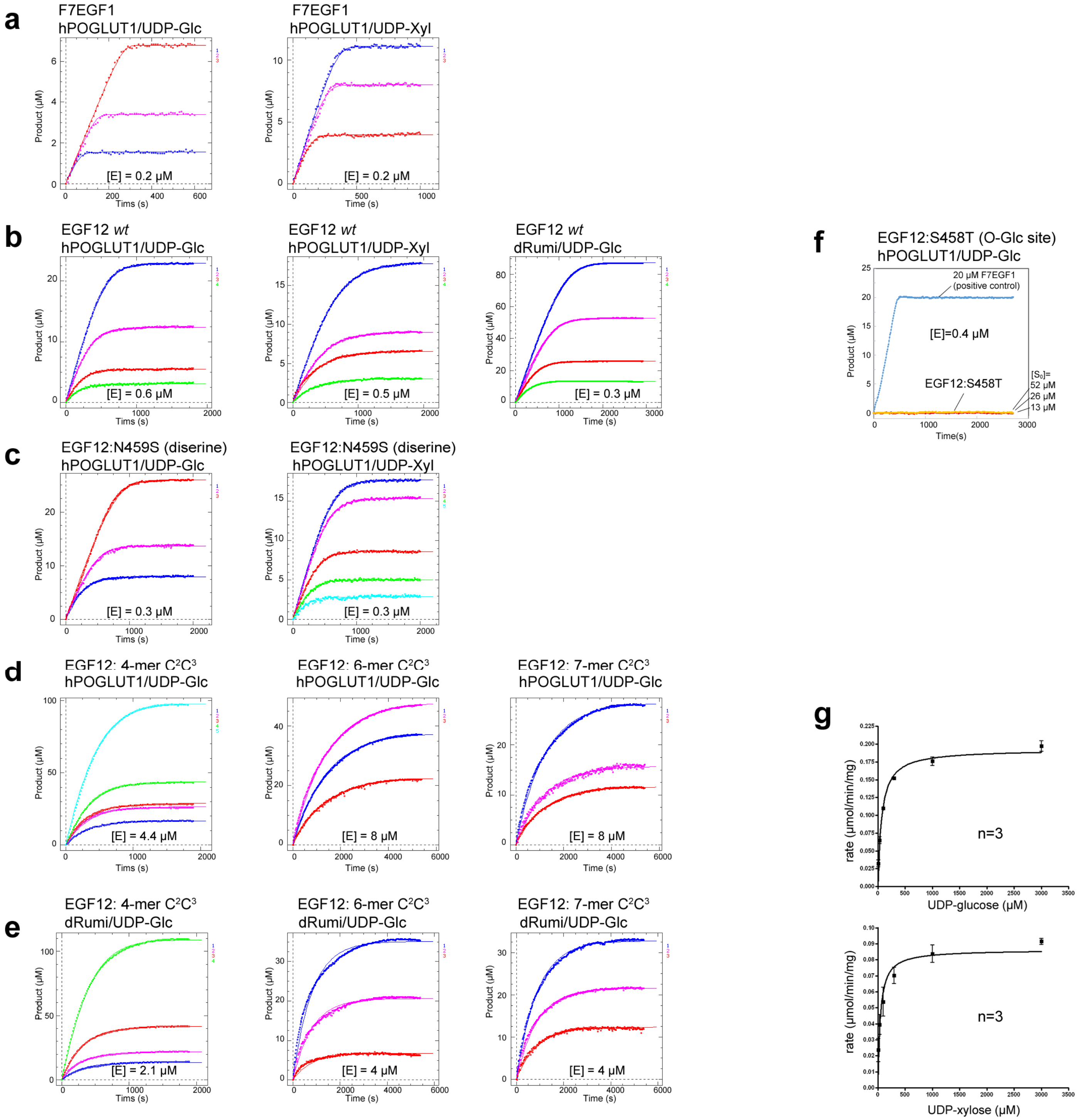

Supplementary Figure 9. Kinetic analysis of POGLUT1 and Rumi

(a-e) Reaction progress curves of hPOGLUT1 and dRumi catalyzed O-glucosylation or O-xylosylation reactions. Data were fitted to a one-substrate model (thin lines) using the program Dynafit. For each experiment, only one of 3-6 repeats is shown. The curves in each panel were globally fit to generate a K_M and a V_{max}. The K_M and V_{max} values from the multiple repeats were used to generate the mean and standard deviation values reported in **Table 1**. In all panels in **d** and **e**, the enzyme concentrations and reaction times were both greatly increased in order to record complete reaction curves. **(f)** The hEGF12:S458T mutant was inactive when tested at 3 different starting concentrations. hF7EGF1 was used as a positive control. **(g)** The K_M values (listed in **Table 1**) of UDP-glucose and UDP-xylose for hPOGLUT1 were determined by non-linear regression fitting of initial velocity vs donor substrate concentration.

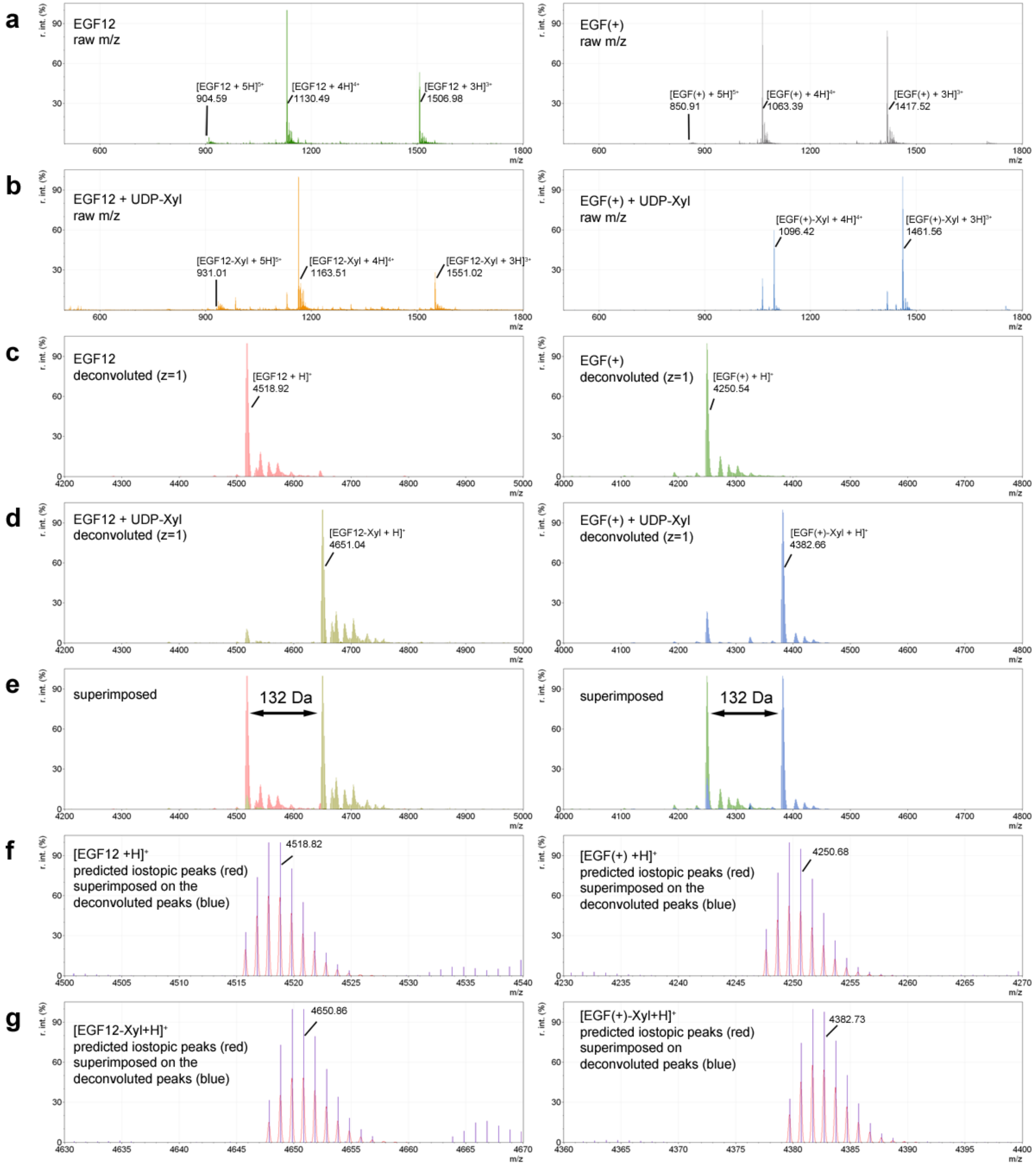

EGF12: C183H275N51O69S7
ave. MW: 4517.91 Da

EGF12-Xyl: C183H275N51O69S7 + C5H8O4
ave. MW: 4650.03 Da

GSDVNECVSN PCQNDATCLD QIGEFQCICM PGYEGVHCEV NT

EGF(+): C171H255N47O68S6
ave. MW: 4249.53 Da

EGF(+)-Xyl: C171H255N47O68S6 + C5H8O4
ave. MW 4381.65 Da

GSDIDECASN PCQNGGTCVN TVGSYTCLCP PGFTGPNCED DI

Supplementary Figure 10. ***in vitro* O-xylosylation of EGF(+) and hEGF12 analyzed by ESI-MS**

Left column, hEGF12; right column, EGF(+). The molecular formula, calculated average molecular weight and protein sequences of the EGF-like domains are shown at the bottom of each column. (**a**) Raw (undeconvoluted) m/z spectra of the bacterially produced EGF-like domains. (**b**) Raw m/z spectra of hPOGLUT1 and UDP-xylose treated EGF-like domains. (**c,d**) Deconvoluted (z = 1) spectra of (**a**) and (**b**), respectively. Most of the lower intensity peaks correspond to identifiable adducts or proteolytic degradation products. (**e**) Superimposition of (**c**) and (**d**). (**f,g**) The major peak in (**c**) and (**d**), respectively, showing the isotopic peaks (thin blue bars). The predicted isotopic peaks (red Gaussian peaks) based on the molecular formulae are superimposed for comparison. Panels (**c,d**), also shown in main text (**Figure 6b,c**), are included here for completeness.

Supplementary Reference

1. Li, Z. et al. Recognition of EGF-like domains by the Notch-modifying O-fucosyltransferase POFUT1. *Nat Chem Biol* (2017). DOI: 10.1038/nchembio.2381
