# Supplementary material for: Structural basis of Notch O-glucosylation and O–xylosylation by mammalian protein–O-glucosyltransferase 1 (POGLUT1)
Source: Nat Commun. 2017 Aug 4;8:185. doi: 10.1038/s41467-017-00255-7 (PMC5543122; doi:10.1038/s41467-017-00255-7)
Supplement: Supplementary file 2 — Supplementary Data 1 [file 41467_2017_255_MOESM2_ESM.pdf]

Supplementary Data 1

Structural basis of Notch O-glucosylation and O-xylosylation by mammalian protein-O-glucosyltransferase 1 (POGLUT1)

Zhijie Li[a,b], Michael Fischer[c], Malathy Satkunarajah[a,b], Dongxia Zhou[a,b], Stephen G. Withers[c], James M Rini[a,b,*]

Affiliations:

[a]Department of Biochemistry, University of Toronto, Toronto, Ontario, M5S 1A8, Canada.

[b]Department of Molecular Genetics, University of Toronto, Toronto, Ontario, M5S 1A8, Canada.

[c]Department of Chemistry, University of British Columbia, Vancouver, British Columbia, V6T 1Z1, Canada.

***To whom correspondence may be addressed, E-mail: james.rini@utoronto.ca**

This file contains statistics for the distribution of EGF-like domain types (hEGF, cEGF, lamEGF, intEGF) and O-glucosylation motifs in 339 animal species. For each species, the number of EGF-like domains of each type (purple bars) and the number of EGF-like domains that contain the O-glucosylation motif (green bars) are shown in the left panel. The percentages of the EGF-like domains containing the O-glucosylation motif in each type are shown in the right panel (the percentages for the intEGFs are not shown due to the small number of intEGFs in each species). The phyla are indicated in the round brackets. Species lacking a POGLUT1/Rumi homologue sequence are marked with "*No POGLUT1/Rumi" in the EGF counts panels.

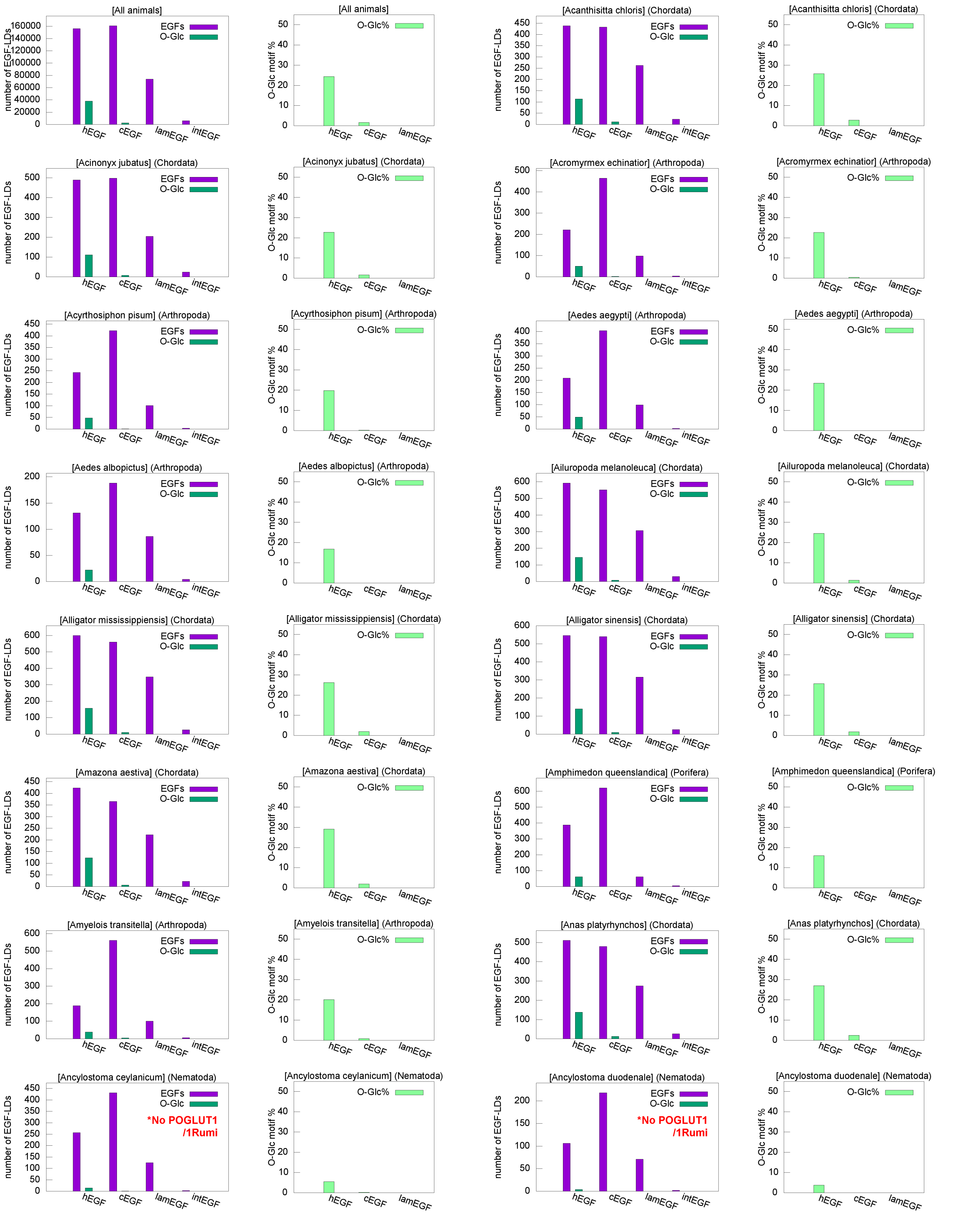
[All animals]
number of EGF-LDs
EGFs
O-Glc
hEGF
cEGF
lamEGF
intEGF
[All animals]
O-Glc motif %
O-Glc%
[Acanthisitta chloris] (Chordata)
[Acanthisitta chloris] (Chordata)
[Acinonyx jubatus] (Chordata)
[Acinonyx jubatus] (Chordata)
[Acromyrmex echinatior] (Arthropoda)
[Acromyrmex echinatior] (Arthropoda)
[Acyrthosiphon pisum] (Arthropoda)
[Acyrthosiphon pisum] (Arthropoda)
[Aedes aegypti] (Arthropoda)
[Aedes aegypti] (Arthropoda)
[Aedes albopictus] (Arthropoda)
[Aedes albopictus] (Arthropoda)
[Ailuropoda melanoleuca] (Chordata)
[Ailuropoda melanoleuca] (Chordata)
[Alligator mississippiensis] (Chordata)
[Alligator mississippiensis] (Chordata)
[Alligator sinensis] (Chordata)
[Alligator sinensis] (Chordata)
[Amazona aestiva] (Chordata)
[Amazona aestiva] (Chordata)
[Amphimedon queenslandica] (Porifera)
[Amphimedon queenslandica] (Porifera)
[Amyelois transitella] (Arthropoda)
[Amyelois transitella] (Arthropoda)
[Anas platyrhynchos] (Chordata)
[Anas platyrhynchos] (Chordata)
[Ancylostoma ceylanicum] (Nematoda)
*No POGLUT1
/1Rumi
[Ancylostoma ceylanicum] (Nematoda)
[Ancylostoma duodenale] (Nematoda)
*No POGLUT1
/1Rumi
[Ancylostoma duodenale] (Nematoda)

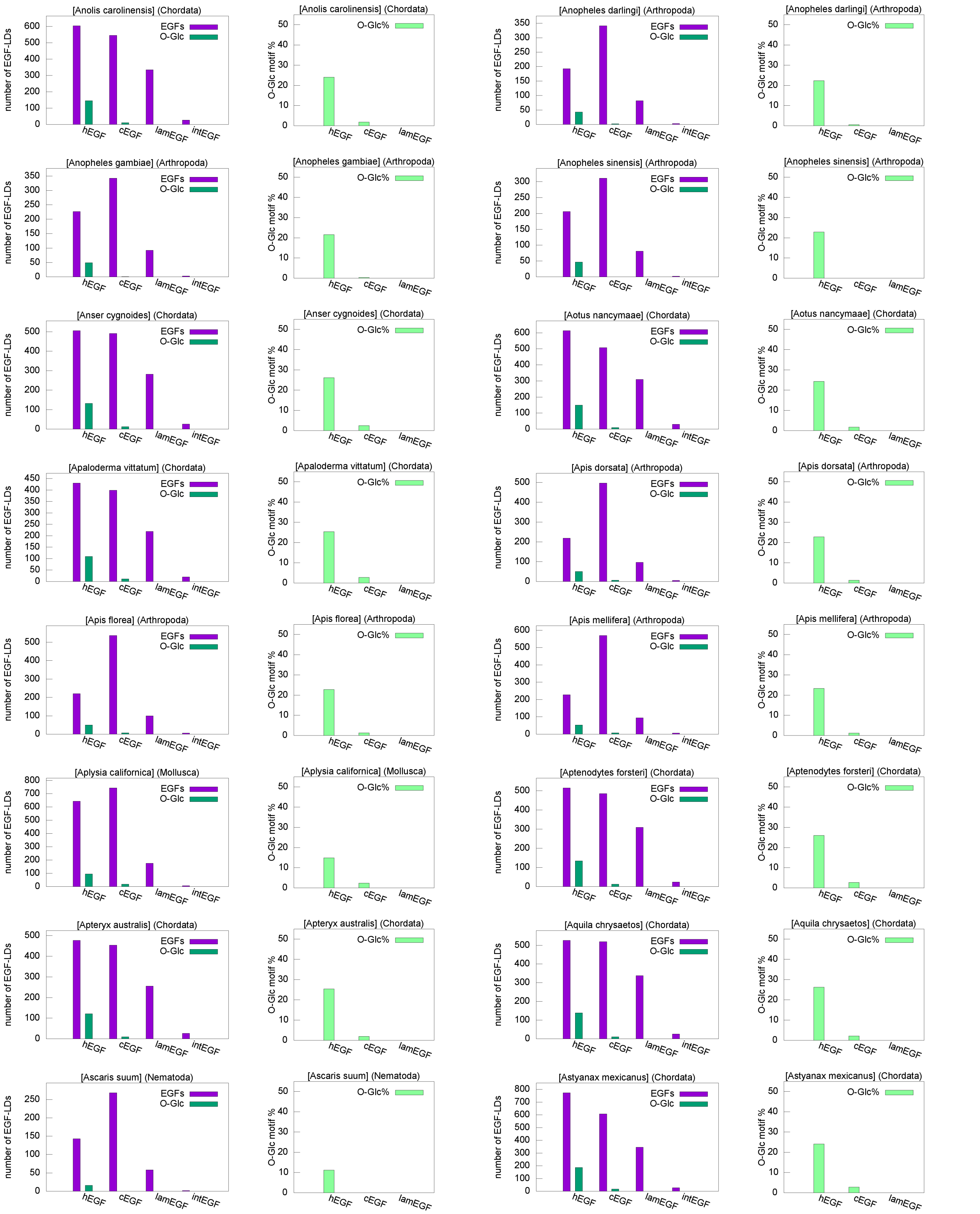
[Anolis carolinensis] (Chordata)
[Anolis carolinensis] (Chordata)
[Anopheles darlingi] (Arthropoda)
[Anopheles darlingi] (Arthropoda)
[Anopheles gambiae] (Arthropoda)
[Anopheles gambiae] (Arthropoda)
[Anopheles sinensis] (Arthropoda)
[Anopheles sinensis] (Arthropoda)
[Anser cygnoides] (Chordata)
[Anser cygnoides] (Chordata)
[Aotus nancymaae] (Chordata)
[Aotus nancymaae] (Chordata)
[Apaloderma vittatum] (Chordata)
[Apaloderma vittatum] (Chordata)
[Apis dorsata] (Arthropoda)
[Apis dorsata] (Arthropoda)
[Apis florea] (Arthropoda)
[Apis florea] (Arthropoda)
[Apis mellifera] (Arthropoda)
[Apis mellifera] (Arthropoda)
[Aplysia californica] (Mollusca)
[Aplysia californica] (Mollusca)
[Aptenodytes forsteri] (Chordata)
[Aptenodytes forsteri] (Chordata)
[Apteryx australis] (Chordata)
[Apteryx australis] (Chordata)
[Aquila chrysaetos] (Chordata)
[Aquila chrysaetos] (Chordata)
[Ascaris suum] (Nematoda)
[Ascaris suum] (Nematoda)
[Astyanax mexicanus] (Chordata)
[Astyanax mexicanus] (Chordata)
number of EGF-LDs
O-Glc motif %
EGFs
O-Glc
O-Glc%
hEGF
cEGF
lamEGF
intEGF

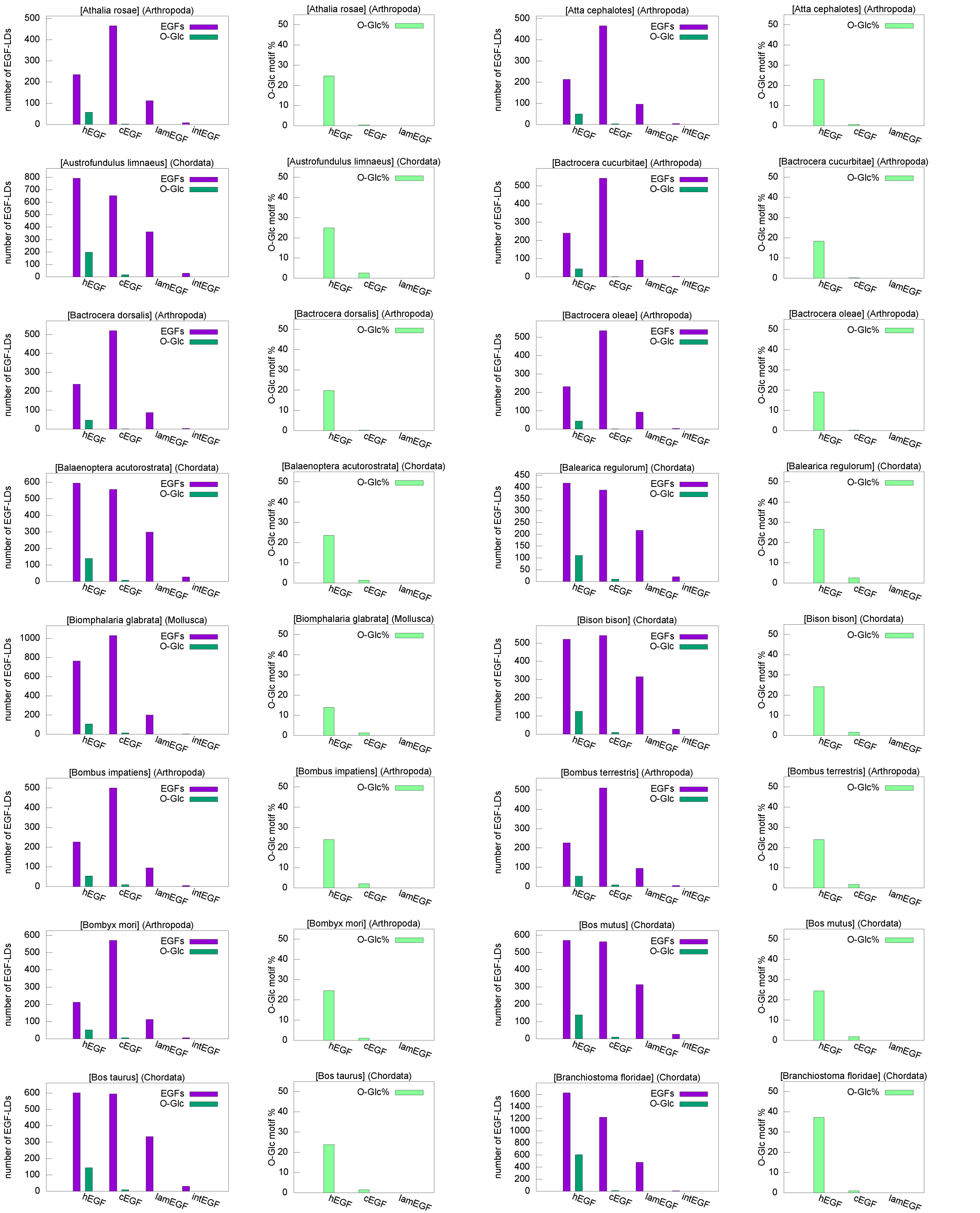
[Athalia rosae] (Arthropoda)
[Athalia rosae] (Arthropoda)
[Atta cephalotes] (Arthropoda)
[Atta cephalotes] (Arthropoda)
[Austrofundulus limnaeus] (Chordata)
[Austrofundulus limnaeus] (Chordata)
[Bactrocera cucurbitae] (Arthropoda)
[Bactrocera cucurbitae] (Arthropoda)
[Bactrocera dorsalis] (Arthropoda)
[Bactrocera dorsalis] (Arthropoda)
[Bactrocera oleae] (Arthropoda)
[Bactrocera oleae] (Arthropoda)
[Balaenoptera acutorostrata] (Chordata)
[Balaenoptera acutorostrata] (Chordata)
[Balearica regulorum] (Chordata)
[Balearica regulorum] (Chordata)
[Biomphalaria glabrata] (Mollusca)
[Biomphalaria glabrata] (Mollusca)
[Bison bison] (Chordata)
[Bison bison] (Chordata)
[Bombus impatiens] (Arthropoda)
[Bombus impatiens] (Arthropoda)
[Bombus terrestris] (Arthropoda)
[Bombus terrestris] (Arthropoda)
[Bombyx mori] (Arthropoda)
[Bombyx mori] (Arthropoda)
[Bos mutus] (Chordata)
[Bos mutus] (Chordata)
[Bos taurus] (Chordata)
[Bos taurus] (Chordata)
[Branchiostoma floridae] (Chordata)
[Branchiostoma floridae] (Chordata)
number of EGF-LDs
O-Glc motif %
EGFs
O-Glc
O-Glc%
hEGF
cEGF
lamEGF
intEGF

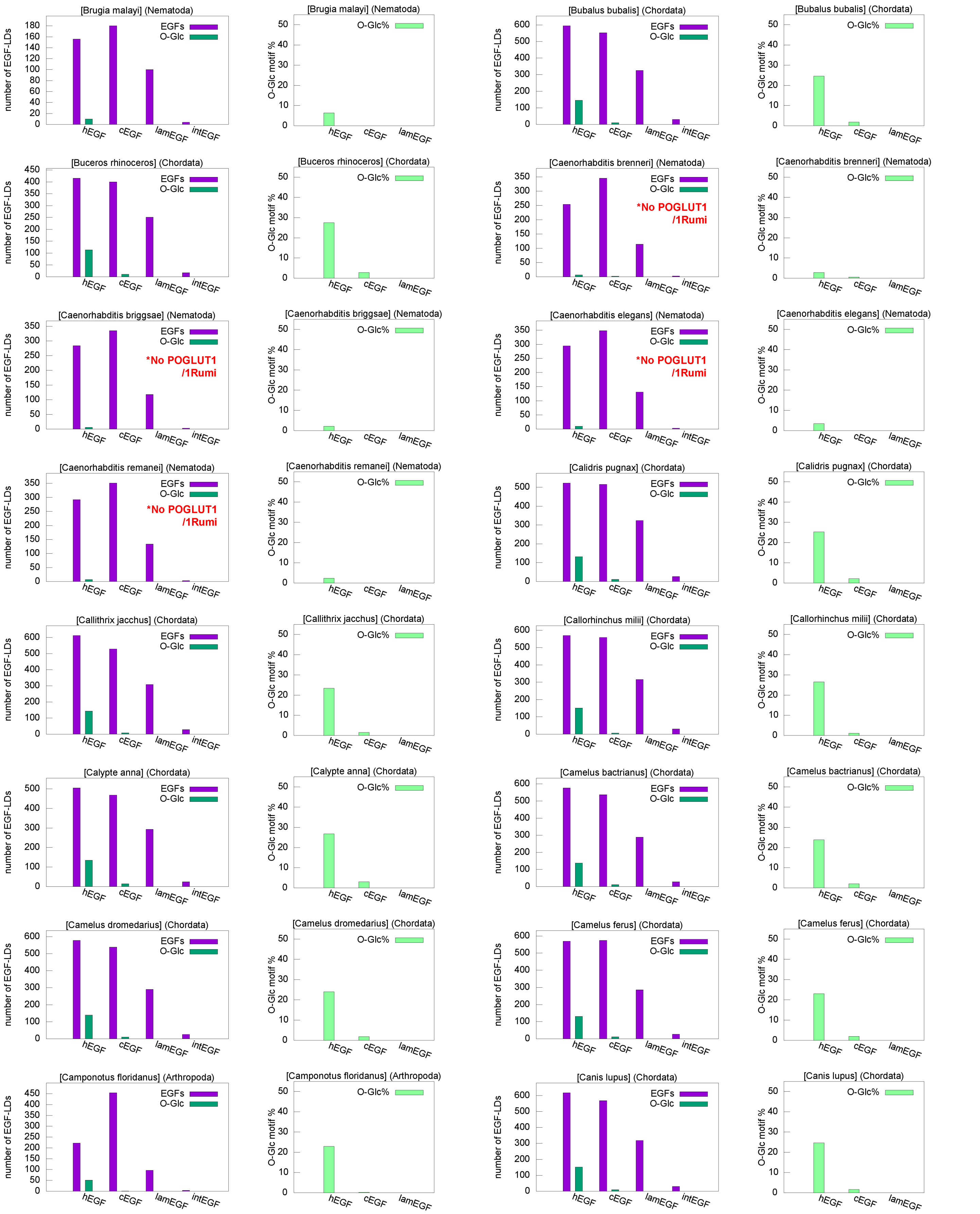

[Brugia malayi] (Nematoda)
[Bubalus bubalis] (Chordata)
[Buceros rhinoceros] (Chordata)
[Caenorhabditis brenneri] (Nematoda)
[Caenorhabditis briggsae] (Nematoda)
[Caenorhabditis elegans] (Nematoda)
[Caenorhabditis remanei] (Nematoda)
[Calidris pugnax] (Chordata)
[Callithrix jacchus] (Chordata)
[Callorhinchus milii] (Chordata)
[Calypte anna] (Chordata)
[Camelus bactrianus] (Chordata)
[Camelus dromedarius] (Chordata)
[Camelus ferus] (Chordata)
[Camponotus floridanus] (Arthropoda)
[Canis lupus] (Chordata)
number of EGF-LDs
O-Glc motif %
EGFs
O-Glc
O-Glc%
*No POGLUT1
/1Rumi
hEGF
cEGF
lamEGF
intEGF

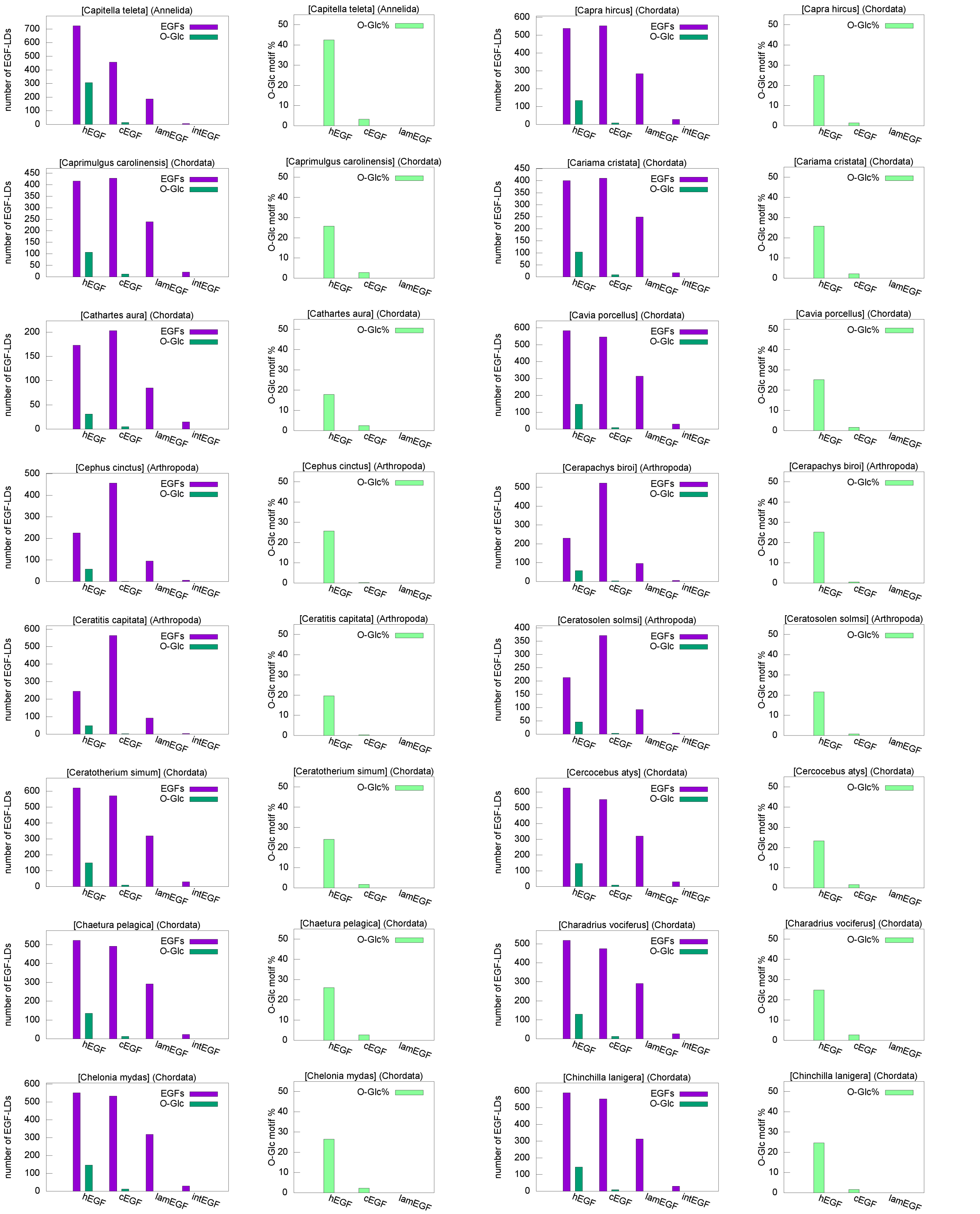
[Capitella teleta] (Annelida)
[Capitella teleta] (Annelida)
[Capra hircus] (Chordata)
[Capra hircus] (Chordata)
[Caprimulgus carolinensis] (Chordata)
[Caprimulgus carolinensis] (Chordata)
[Cariama cristata] (Chordata)
[Cariama cristata] (Chordata)
[Cathartes aura] (Chordata)
[Cathartes aura] (Chordata)
[Cavia porcellus] (Chordata)
[Cavia porcellus] (Chordata)
[Cephus cinctus] (Arthropoda)
[Cephus cinctus] (Arthropoda)
[Cerapachys biroi] (Arthropoda)
[Cerapachys biroi] (Arthropoda)
[Ceratitis capitata] (Arthropoda)
[Ceratitis capitata] (Arthropoda)
[Ceratosolen solmsi] (Arthropoda)
[Ceratosolen solmsi] (Arthropoda)
[Ceratotherium simum] (Chordata)
[Ceratotherium simum] (Chordata)
[Cercocebus atys] (Chordata)
[Cercocebus atys] (Chordata)
[Chaetura pelagica] (Chordata)
[Chaetura pelagica] (Chordata)
[Charadrius vociferus] (Chordata)
[Charadrius vociferus] (Chordata)
[Chelonia mydas] (Chordata)
[Chelonia mydas] (Chordata)
[Chinchilla lanigera] (Chordata)
[Chinchilla lanigera] (Chordata)
number of EGF-LDs
O-Glc motif %
EGFs
O-Glc
O-Glc%
hEGF
cEGF
lamEGF
intEGF

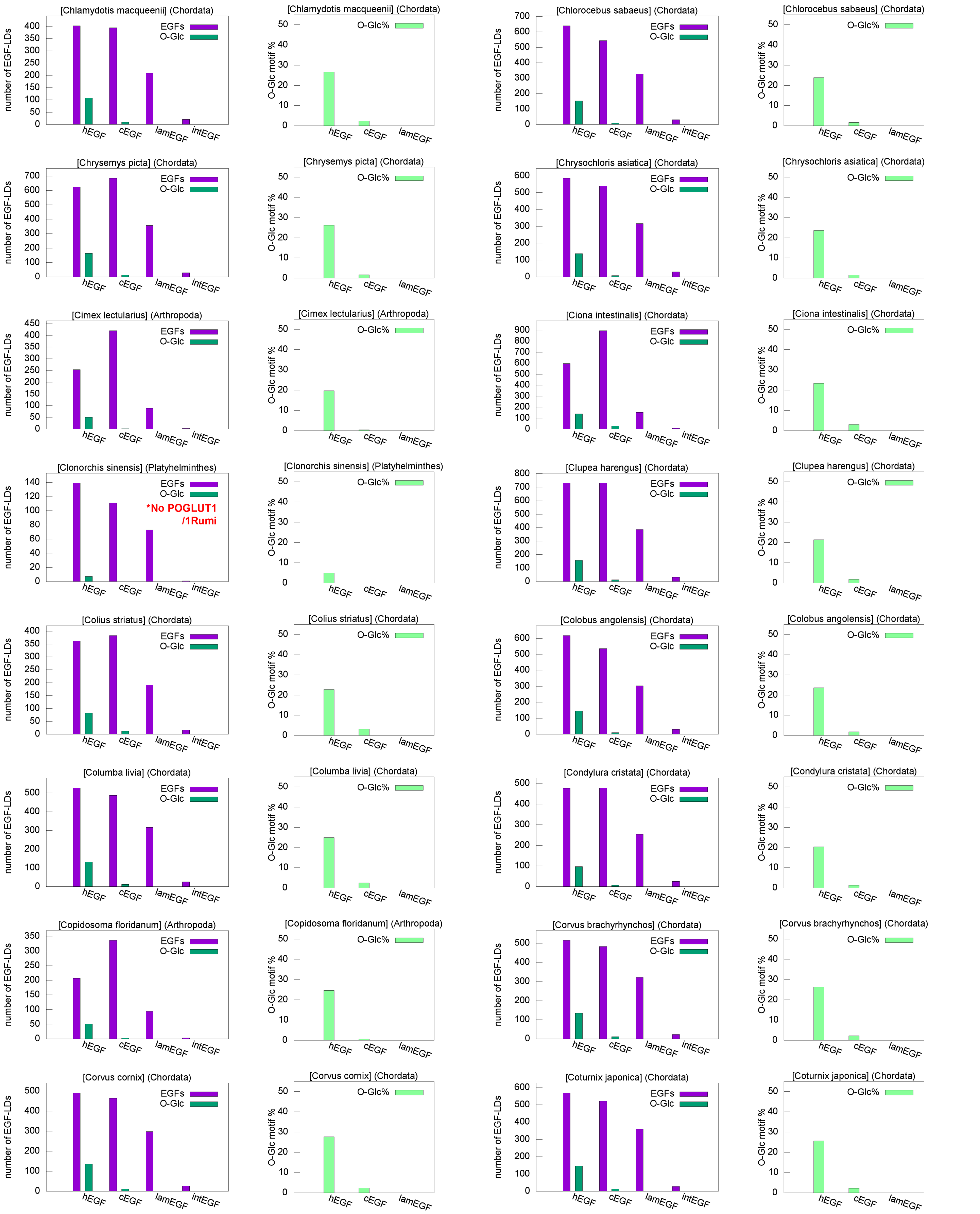
[Chlamydotis macqueenii] (Chordata)
[Chlamydotis macqueenii] (Chordata)
[Chlorocebus sabaeus] (Chordata)
[Chlorocebus sabaeus] (Chordata)
[Chrysemys picta] (Chordata)
[Chrysemys picta] (Chordata)
[Chrysochloris asiatica] (Chordata)
[Chrysochloris asiatica] (Chordata)
[Cimex lectularius] (Arthropoda)
[Cimex lectularius] (Arthropoda)
[Ciona intestinalis] (Chordata)
[Ciona intestinalis] (Chordata)
[Clonorchis sinensis] (Platyhelminthes)
*No POGLUT1
/1Rumi
[Clonorchis sinensis] (Platyhelminthes)
[Clupea harengus] (Chordata)
[Clupea harengus] (Chordata)
[Colius striatus] (Chordata)
[Colius striatus] (Chordata)
[Colobus angolensis] (Chordata)
[Colobus angolensis] (Chordata)
[Columba livia] (Chordata)
[Columba livia] (Chordata)
[Condylura cristata] (Chordata)
[Condylura cristata] (Chordata)
[Copidosoma floridanum] (Arthropoda)
[Copidosoma floridanum] (Arthropoda)
[Corvus brachyrhynchos] (Chordata)
[Corvus brachyrhynchos] (Chordata)
[Corvus cornix] (Chordata)
[Corvus cornix] (Chordata)
[Coturnix japonica] (Chordata)
[Coturnix japonica] (Chordata)
number of EGF-LDs
O-Glc motif %
EGFs
O-Glc
O-Glc%
hEGF
cEGF
lamEGF
intEGF

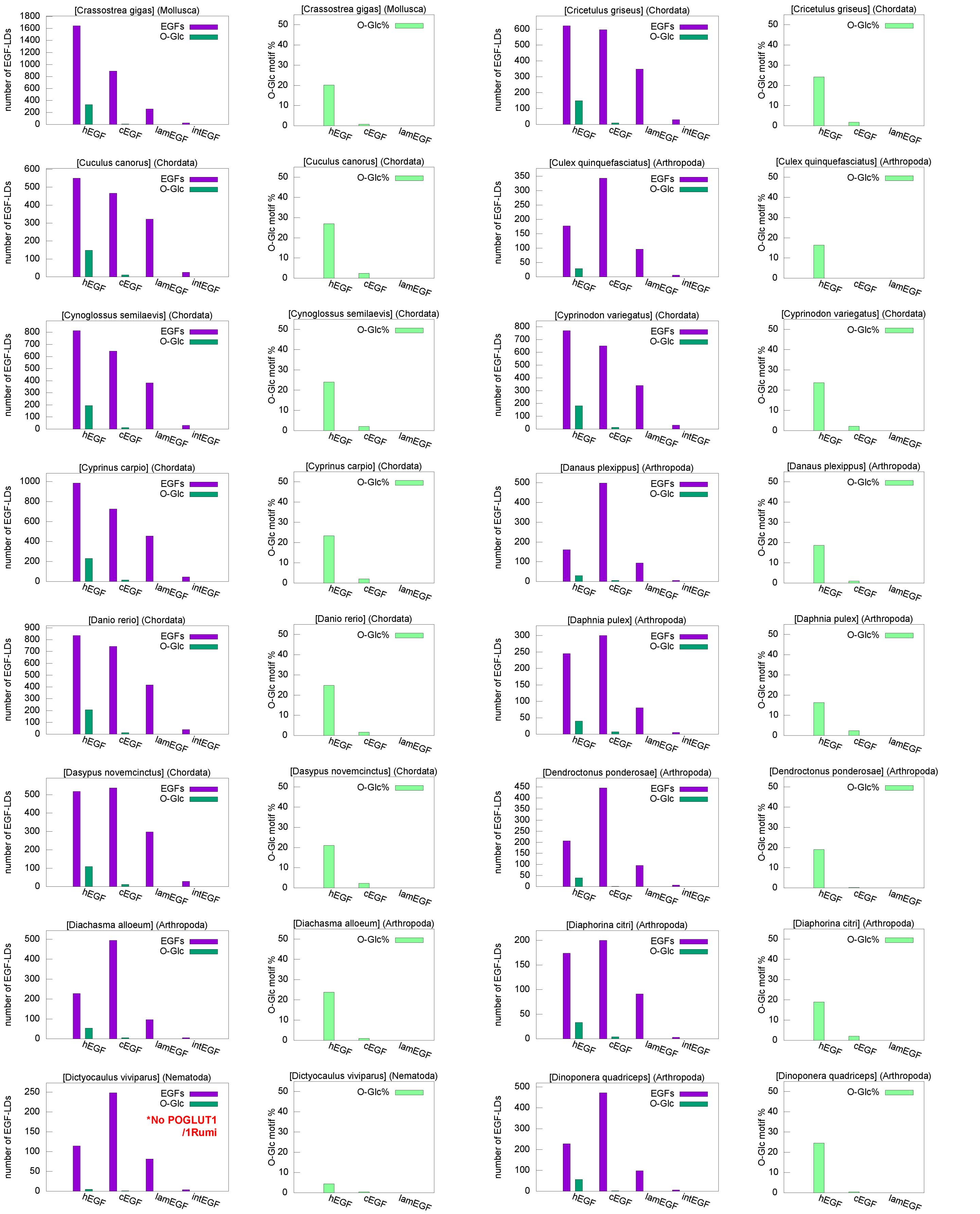
[Crassostrea gigas] (Mollusca)
[Cricetulus griseus] (Chordata)
[Cuculus canorus] (Chordata)
[Culex quinquefasciatus] (Arthropoda)
[Cynoglossus semilaevis] (Chordata)
[Cyprinodon variegatus] (Chordata)
[Cyprinus carpio] (Chordata)
[Danaus plexippus] (Arthropoda)
[Danio rerio] (Chordata)
[Daphnia pulex] (Arthropoda)
[Dasypus novemcinctus] (Chordata)
[Dendroctonus ponderosae] (Arthropoda)
[Diachasma alloeum] (Arthropoda)
[Diaphorina citri] (Arthropoda)
[Dictyocaulus viviparus] (Nematoda)
[Dinoponera quadriceps] (Arthropoda)
number of EGF-LDs
O-Glc motif %
EGFs
O-Glc
O-Glc%
hEGF
cEGF
lamEGF
intEGF
*No POGLUT1
/1Rumi

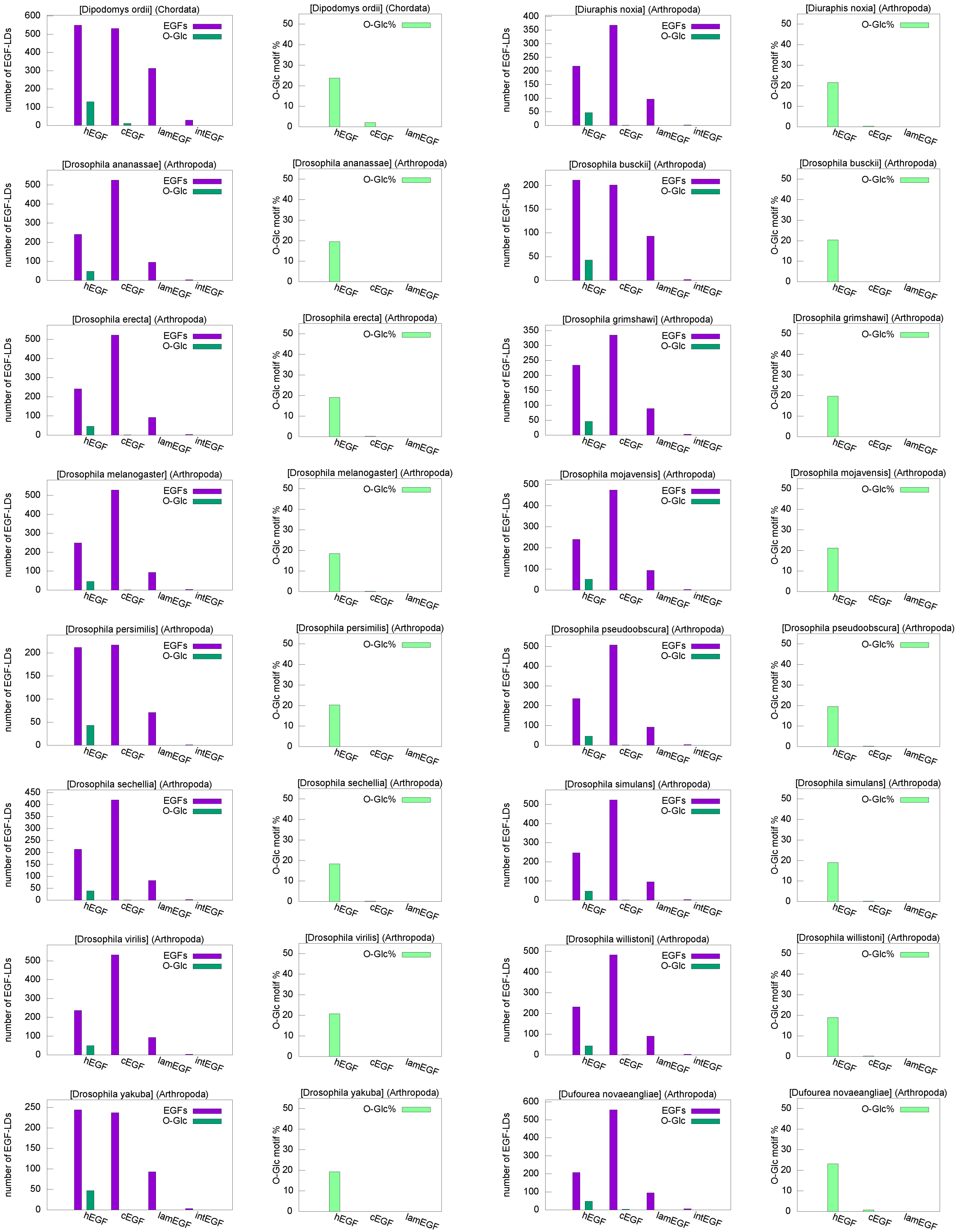
[Dipodomys ordii] (Chordata)
[Dipodomys ordii] (Chordata)
[Diuraphis noxia] (Arthropoda)
[Diuraphis noxia] (Arthropoda)
[Drosophila ananassae] (Arthropoda)
[Drosophila ananassae] (Arthropoda)
[Drosophila busckii] (Arthropoda)
[Drosophila busckii] (Arthropoda)
[Drosophila erecta] (Arthropoda)
[Drosophila erecta] (Arthropoda)
[Drosophila grimshawi] (Arthropoda)
[Drosophila grimshawi] (Arthropoda)
[Drosophila melanogaster] (Arthropoda)
[Drosophila melanogaster] (Arthropoda)
[Drosophila mojavensis] (Arthropoda)
[Drosophila mojavensis] (Arthropoda)
[Drosophila persimilis] (Arthropoda)
[Drosophila persimilis] (Arthropoda)
[Drosophila pseudoobscura] (Arthropoda)
[Drosophila pseudoobscura] (Arthropoda)
[Drosophila sechellia] (Arthropoda)
[Drosophila sechellia] (Arthropoda)
[Drosophila simulans] (Arthropoda)
[Drosophila simulans] (Arthropoda)
[Drosophila virilis] (Arthropoda)
[Drosophila virilis] (Arthropoda)
[Drosophila willistoni] (Arthropoda)
[Drosophila willistoni] (Arthropoda)
[Drosophila yakuba] (Arthropoda)
[Drosophila yakuba] (Arthropoda)
[Dufourea novaeangliae] (Arthropoda)
[Dufourea novaeangliae] (Arthropoda)
number of EGF-LDs
O-Glc motif %
EGFs
O-Glc
O-Glc%
hEGF
cEGF
lamEGF
intEGF

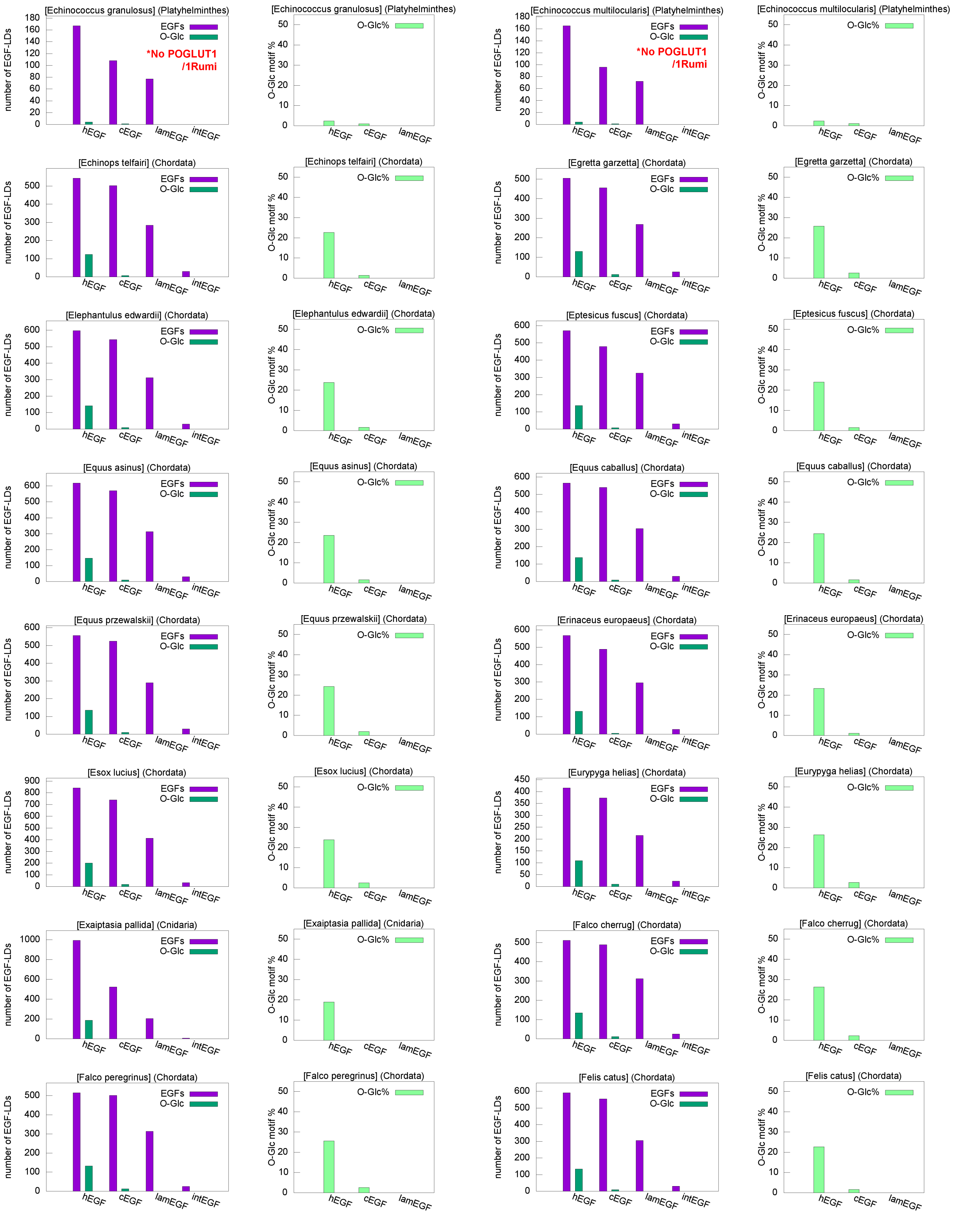
[Echinococcus granulosus] (Platyhelminthes)
EGFs
O-Glc
*No POGLUT1
/1Rumi
number of EGF-LDs
hEGF
cEGF
lamEGF
intEGF
O-Glc%
O-Glc motif %
[Echinococcus multilocularis] (Platyhelminthes)
[Echinops telfairi] (Chordata)
[Egretta garzetta] (Chordata)
[Elephantulus edwardii] (Chordata)
[Eptesicus fuscus] (Chordata)
[Equus asinus] (Chordata)
[Equus caballus] (Chordata)
[Equus przewalskii] (Chordata)
[Erinaceus europaeus] (Chordata)
[Esox lucius] (Chordata)
[Eurypyga helias] (Chordata)
[Exaiptasia pallida] (Cnidaria)
[Falco cherrug] (Chordata)
[Falco peregrinus] (Chordata)
[Felis catus] (Chordata)

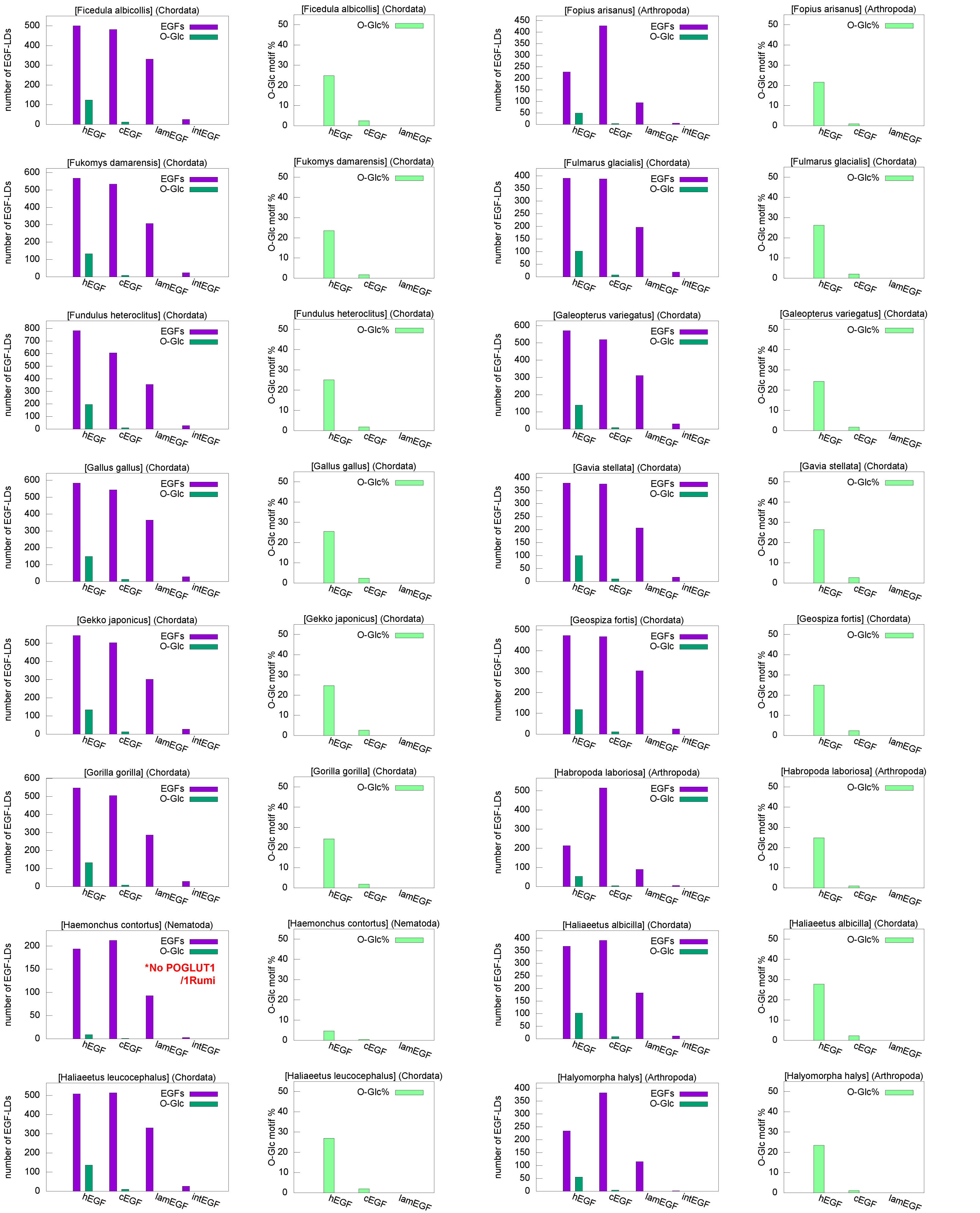
[Ficedula albicollis] (Chordata)
number of EGF-LDs
EGFs
O-Glc
hEGF cEGF lamEGF intEGF
[Ficedula albicollis] (Chordata)
O-Glc motif %
O-Glc%
hEGF cEGF lamEGF
[Fopius arisanus] (Arthropoda)
number of EGF-LDs
EGFs
O-Glc
hEGF cEGF lamEGF intEGF
[Fopius arisanus] (Arthropoda)
O-Glc motif %
O-Glc%
hEGF cEGF lamEGF
[Fukomys damarensis] (Chordata)
[Fukomys damarensis] (Chordata)
[Fulmarus glacialis] (Chordata)
[Fulmarus glacialis] (Chordata)
[Fundulus heteroclitus] (Chordata)
[Fundulus heteroclitus] (Chordata)
[Galeopterus variegatus] (Chordata)
[Galeopterus variegatus] (Chordata)
[Gallus gallus] (Chordata)
[Gallus gallus] (Chordata)
[Gavia stellata] (Chordata)
[Gavia stellata] (Chordata)
[Gekko japonicus] (Chordata)
[Gekko japonicus] (Chordata)
[Geospiza fortis] (Chordata)
[Geospiza fortis] (Chordata)
[Gorilla gorilla] (Chordata)
[Gorilla gorilla] (Chordata)
[Habropoda laboriosa] (Arthropoda)
[Habropoda laboriosa] (Arthropoda)
[Haemonchus contortus] (Nematoda)
*No POGLUT1
/1Rumi
[Haemonchus contortus] (Nematoda)
[Haliaeetus albicilla] (Chordata)
[Haliaeetus albicilla] (Chordata)
[Haliaeetus leucocephalus] (Chordata)
[Haliaeetus leucocephalus] (Chordata)
[Halyomorpha halys] (Arthropoda)
[Halyomorpha halys] (Arthropoda)

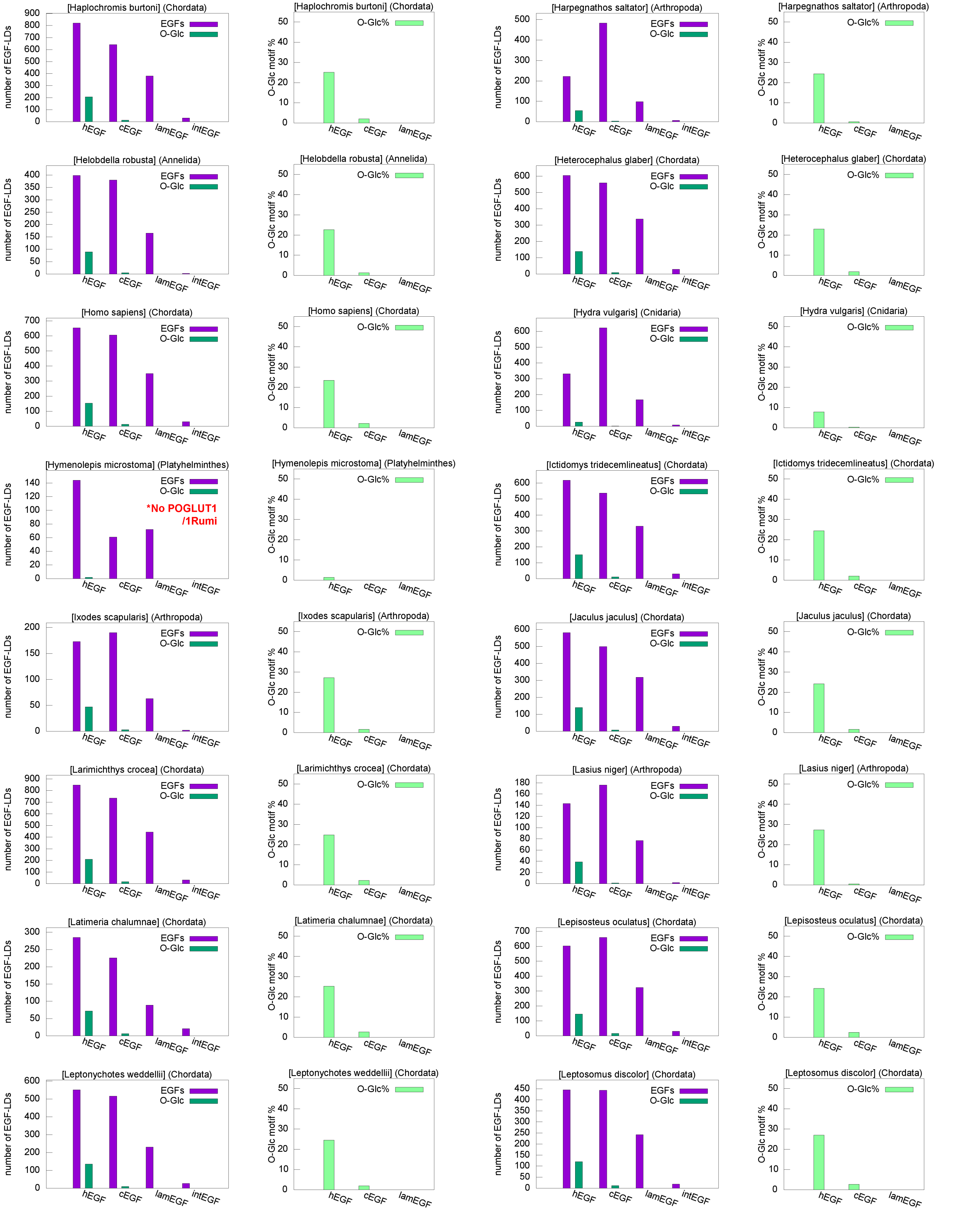
[Haplochromis burtoni] (Chordata)
number of EGF-LDs
EGFs
O-Glc
hEGF cEGF lamEGF intEGF
[Haplochromis burtoni] (Chordata)
O-Glc motif %
O-Glc%
hEGF cEGF lamEGF
[Harpegnathos saltator] (Arthropoda)
number of EGF-LDs
EGFs
O-Glc
hEGF cEGF lamEGF intEGF
[Harpegnathos saltator] (Arthropoda)
O-Glc motif %
O-Glc%
hEGF cEGF lamEGF
[Helobdella robusta] (Annelida)
number of EGF-LDs
EGFs
O-Glc
hEGF cEGF lamEGF intEGF
[Helobdella robusta] (Annelida)
O-Glc motif %
O-Glc%
hEGF cEGF lamEGF
[Heterocephalus glaber] (Chordata)
number of EGF-LDs
EGFs
O-Glc
hEGF cEGF lamEGF intEGF
[Heterocephalus glaber] (Chordata)
O-Glc motif %
O-Glc%
hEGF cEGF lamEGF
[Homo sapiens] (Chordata)
number of EGF-LDs
EGFs
O-Glc
hEGF cEGF lamEGF intEGF
[Homo sapiens] (Chordata)
O-Glc motif %
O-Glc%
hEGF cEGF lamEGF
[Hydra vulgaris] (Cnidaria)
number of EGF-LDs
EGFs
O-Glc
hEGF cEGF lamEGF intEGF
[Hydra vulgaris] (Cnidaria)
O-Glc motif %
O-Glc%
hEGF cEGF lamEGF
[Hymenolepis microstoma] (Platyhelminthes)
number of EGF-LDs
EGFs
O-Glc
*No POGLUT1
/1Rumi
hEGF cEGF lamEGF intEGF
[Hymenolepis microstoma] (Platyhelminthes)
O-Glc motif %
O-Glc%
hEGF cEGF lamEGF
[Ictidomys tridecemlineatus] (Chordata)
number of EGF-LDs
EGFs
O-Glc
hEGF cEGF lamEGF intEGF
[Ictidomys tridecemlineatus] (Chordata)
O-Glc motif %
O-Glc%
hEGF cEGF lamEGF
[Ixodes scapularis] (Arthropoda)
number of EGF-LDs
EGFs
O-Glc
hEGF cEGF lamEGF intEGF
[Ixodes scapularis] (Arthropoda)
O-Glc motif %
O-Glc%
hEGF cEGF lamEGF
[Jaculus jaculus] (Chordata)
number of EGF-LDs
EGFs
O-Glc
hEGF cEGF lamEGF intEGF
[Jaculus jaculus] (Chordata)
O-Glc motif %
O-Glc%
hEGF cEGF lamEGF
[Larimichthys crocea] (Chordata)
number of EGF-LDs
EGFs
O-Glc
hEGF cEGF lamEGF intEGF
[Larimichthys crocea] (Chordata)
O-Glc motif %
O-Glc%
hEGF cEGF lamEGF
[Lasius niger] (Arthropoda)
number of EGF-LDs
EGFs
O-Glc
hEGF cEGF lamEGF intEGF
[Lasius niger] (Arthropoda)
O-Glc motif %
O-Glc%
hEGF cEGF lamEGF
[Latimeria chalumnae] (Chordata)
number of EGF-LDs
EGFs
O-Glc
hEGF cEGF lamEGF intEGF
[Latimeria chalumnae] (Chordata)
O-Glc motif %
O-Glc%
hEGF cEGF lamEGF
[Lepisosteus oculatus] (Chordata)
number of EGF-LDs
EGFs
O-Glc
hEGF cEGF lamEGF intEGF
[Lepisosteus oculatus] (Chordata)
O-Glc motif %
O-Glc%
hEGF cEGF lamEGF
[Leptonychotes weddellii] (Chordata)
number of EGF-LDs
EGFs
O-Glc
hEGF cEGF lamEGF intEGF
[Leptonychotes weddellii] (Chordata)
O-Glc motif %
O-Glc%
hEGF cEGF lamEGF
[Leptosomus discolor] (Chordata)
number of EGF-LDs
EGFs
O-Glc
hEGF cEGF lamEGF intEGF
[Leptosomus discolor] (Chordata)
O-Glc motif %
O-Glc%
hEGF cEGF lamEGF

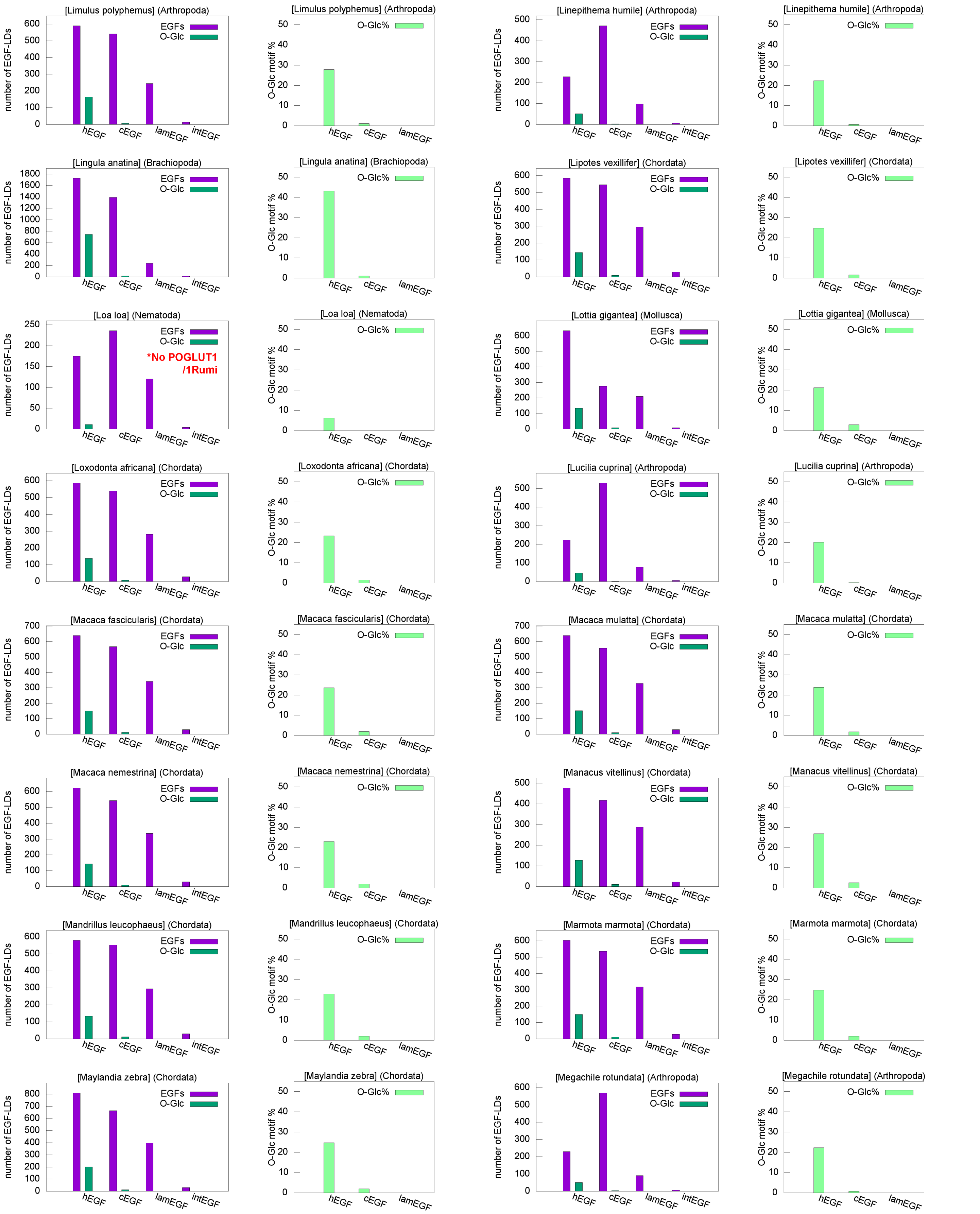

[Limulus polyphemus] (Arthropoda)
[Linepithema humile] (Arthropoda)
[Lingula anatina] (Brachiopoda)
[Lipotes vexillifer] (Chordata)
[Loa loa] (Nematoda)
*No POGLUT1
/1Rumi
[Lottia gigantea] (Mollusca)
[Loxodonta africana] (Chordata)
[Lucilia cuprina] (Arthropoda)
[Macaca fascicularis] (Chordata)
[Macaca mulatta] (Chordata)
[Macaca nemestrina] (Chordata)
[Manacus vitellinus] (Chordata)
[Mandrillus leucophaeus] (Chordata)
[Marmota marmota] (Chordata)
[Maylandia zebra] (Chordata)
[Megachile rotundata] (Arthropoda)
number of EGF-LDs
O-Glc motif %
EGFs
O-Glc
O-Glc%
hEGF
cEGF
lamEGF
intEGF

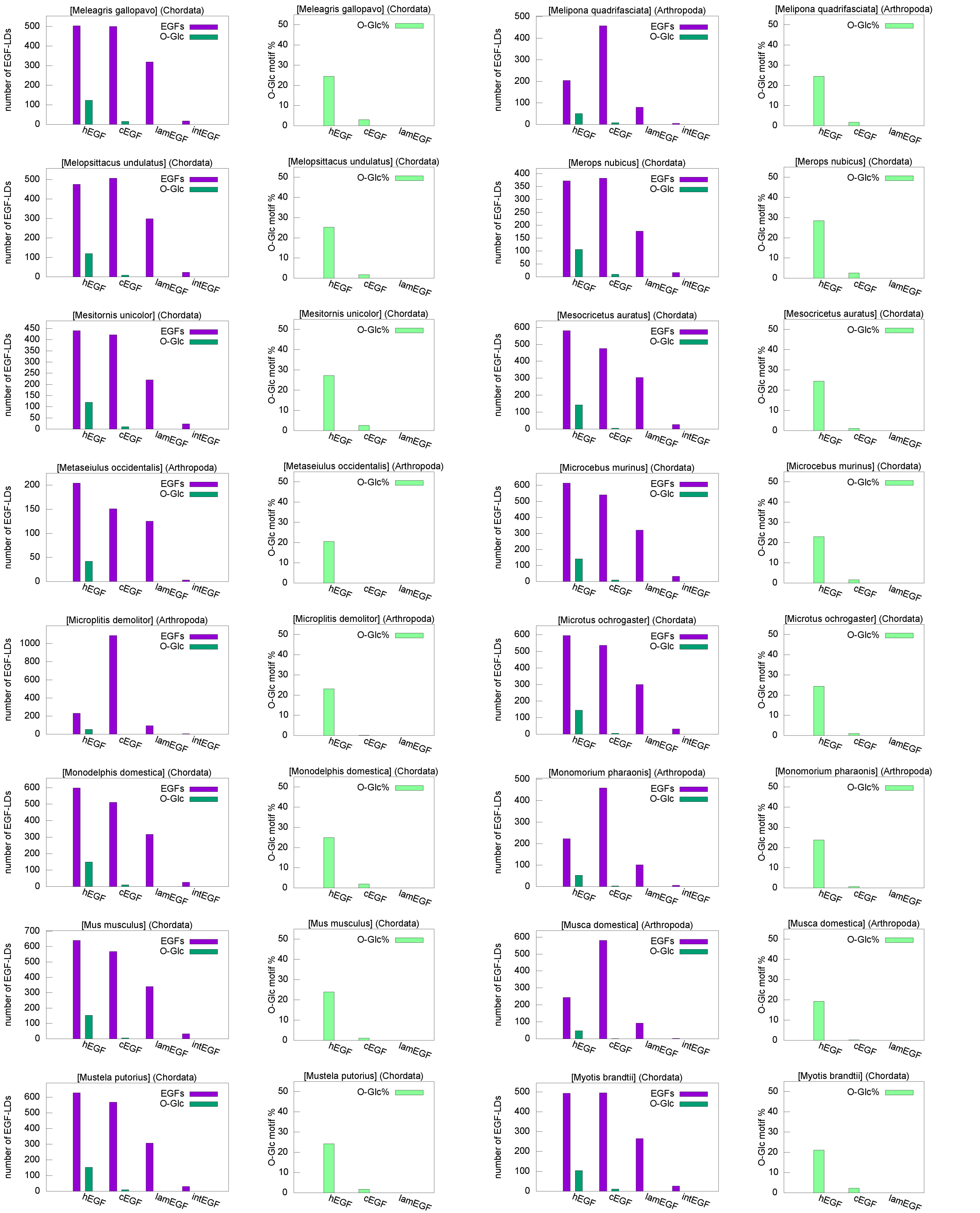
[Meleagris gallopavo] (Chordata)
[Meleagris gallopavo] (Chordata)
[Melipona quadrifasciata] (Arthropoda)
[Melipona quadrifasciata] (Arthropoda)
[Melopsittacus undulatus] (Chordata)
[Melopsittacus undulatus] (Chordata)
[Merops nubicus] (Chordata)
[Merops nubicus] (Chordata)
[Mesitornis unicolor] (Chordata)
[Mesitornis unicolor] (Chordata)
[Mesocricetus auratus] (Chordata)
[Mesocricetus auratus] (Chordata)
[Metaseiulus occidentalis] (Arthropoda)
[Metaseiulus occidentalis] (Arthropoda)
[Microcebus murinus] (Chordata)
[Microcebus murinus] (Chordata)
[Microplitis demolitor] (Arthropoda)
[Microplitis demolitor] (Arthropoda)
[Microtus ochrogaster] (Chordata)
[Microtus ochrogaster] (Chordata)
[Monodelphis domestica] (Chordata)
[Monodelphis domestica] (Chordata)
[Monomorium pharaonis] (Arthropoda)
[Monomorium pharaonis] (Arthropoda)
[Mus musculus] (Chordata)
[Mus musculus] (Chordata)
[Musca domestica] (Arthropoda)
[Musca domestica] (Arthropoda)
[Mustela putorius] (Chordata)
[Mustela putorius] (Chordata)
[Myotis brandtii] (Chordata)
[Myotis brandtii] (Chordata)
number of EGF-LDs
O-Glc motif %
EGFs
O-Glc
O-Glc%
hEGF
cEGF
lamEGF
intEGF

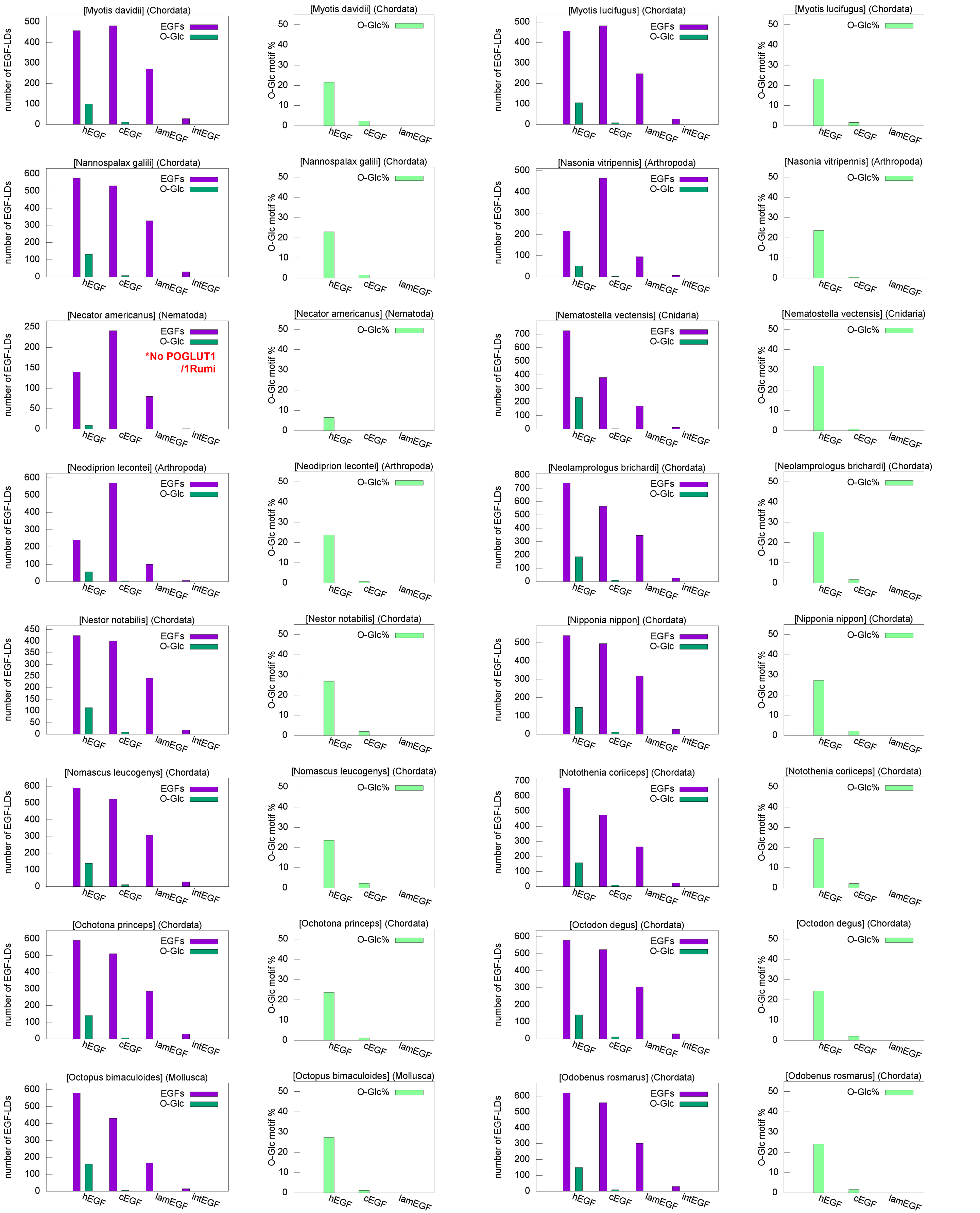
[Myotis davidii] (Chordata)
[Myotis lucifugus] (Chordata)
[Nannospalax galili] (Chordata)
[Nasonia vitripennis] (Arthropoda)
[Necator americanus] (Nematoda)
*No POGLUT1
/1Rumi
[Nematostella vectensis] (Cnidaria)
[Neodiprion lecontei] (Arthropoda)
[Neolamprologus brichardi] (Chordata)
[Nestor notabilis] (Chordata)
[Nipponia nippon] (Chordata)
[Nomascus leucogenys] (Chordata)
[Nototheniа coriiceps] (Chordata)
[Ochotona princeps] (Chordata)
[Octodon degus] (Chordata)
[Octopus bimaculoides] (Mollusca)
[Odobenus rosmarus] (Chordata)
EGFs
O-Glc
O-Glc%
number of EGF-LDs
O-Glc motif %
hEGF
cEGF
lamEGF
intEGF

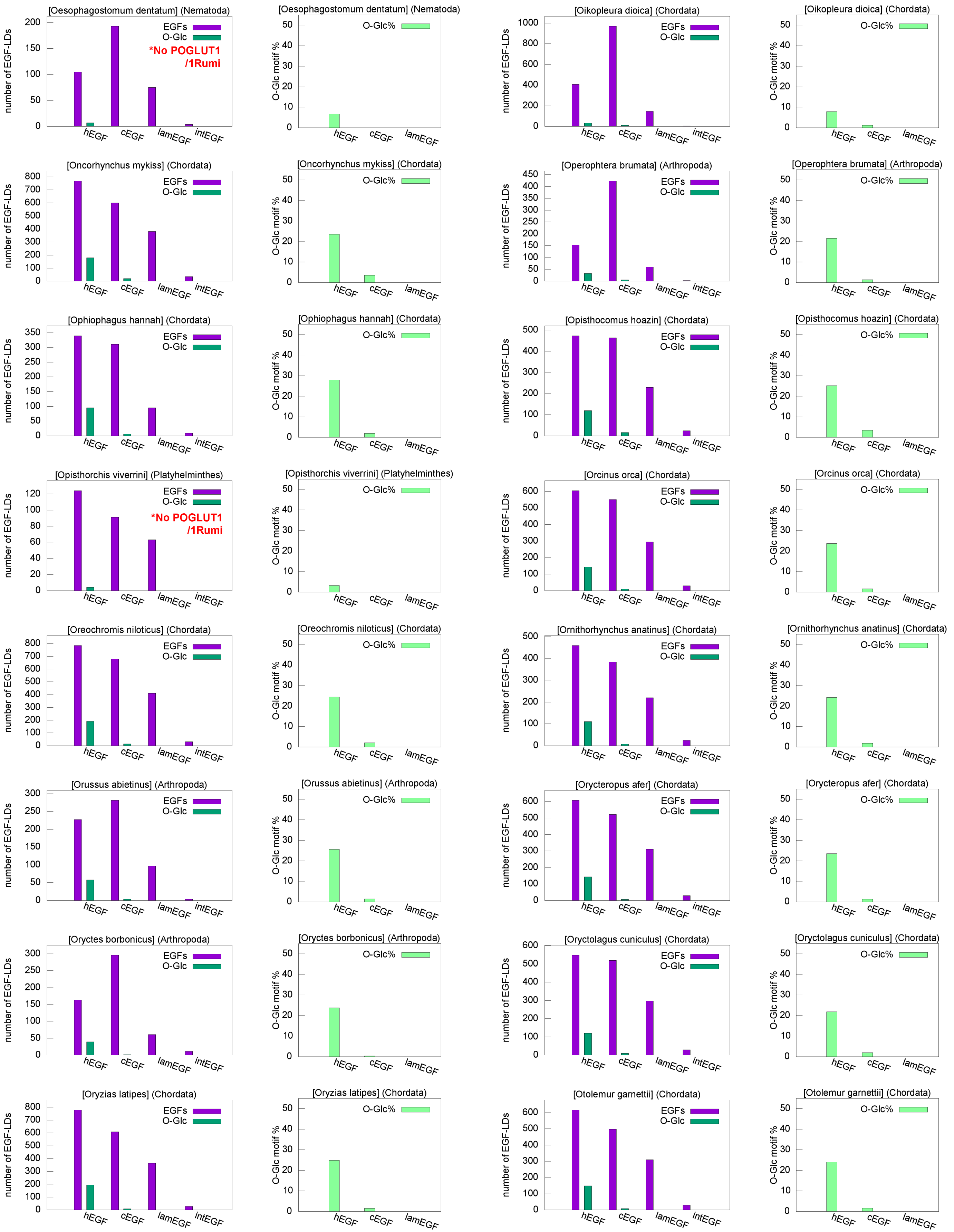
[Oesophagostomum dentatum] (Nematoda)
*No POGLUT1 /1Rumi
[Oikopleura dioica] (Chordata)
[Oncorhynchus mykiss] (Chordata)
[Operophtera brumata] (Arthropoda)
[Ophiophagus hannah] (Chordata)
[Opisthocomus hoazin] (Chordata)
[Opisthorchis viverrini] (Platyhelminthes)
*No POGLUT1 /1Rumi
[Orcinus orca] (Chordata)
[Oreochromis niloticus] (Chordata)
[Ornithorhynchus anatinus] (Chordata)
[Orussus abietinus] (Arthropoda)
[Orycteropus afer] (Chordata)
[Oryctes borbonicus] (Arthropoda)
[Oryctolagus cuniculus] (Chordata)
[Oryzias latipes] (Chordata)
[Otolemur garnettii] (Chordata)
number of EGF-LDs
O-Glc motif %
EGFs
O-Glc
O-Glc%
hEGF
cEGF
lamEGF
intEGF

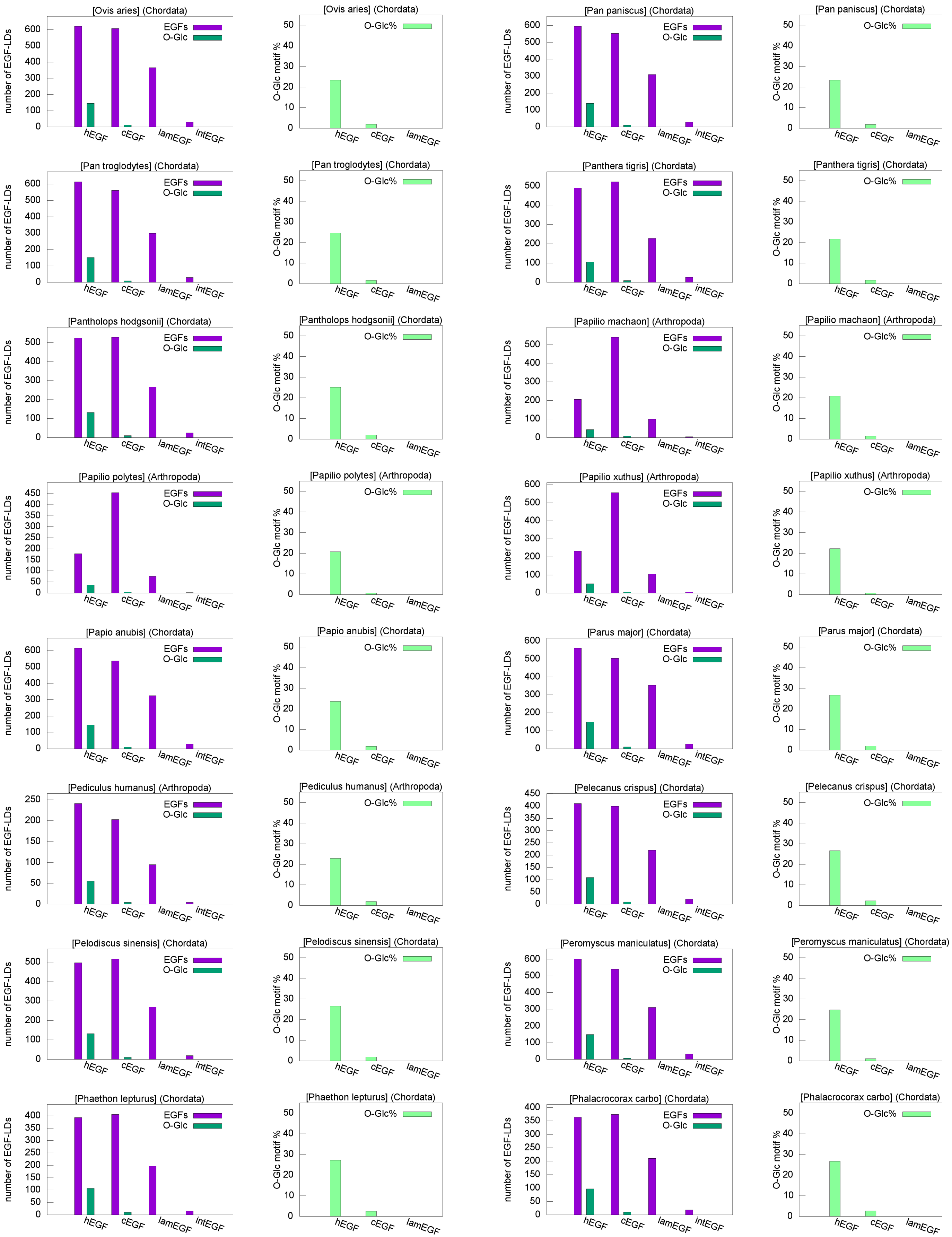
[Ovis aries] (Chordata)
[Pan paniscus] (Chordata)
[Pan troglodytes] (Chordata)
[Panthera tigris] (Chordata)
[Pantholops hodgsonii] (Chordata)
[Papilio machaon] (Arthropoda)
[Papilio polytes] (Arthropoda)
[Papilio xuthus] (Arthropoda)
[Papio anubis] (Chordata)
[Parus major] (Chordata)
[Pediculus humanus] (Arthropoda)
[Pelecanus crispus] (Chordata)
[Pelodiscus sinensis] (Chordata)
[Peromyscus maniculatus] (Chordata)
[Phaethon lepturus] (Chordata)
[Phalacrocorax carbo] (Chordata)
number of EGF-LDs
O-Glc motif %
EGFs
O-Glc
O-Glc%
hEGF
cEGF
lamEGF
intEGF

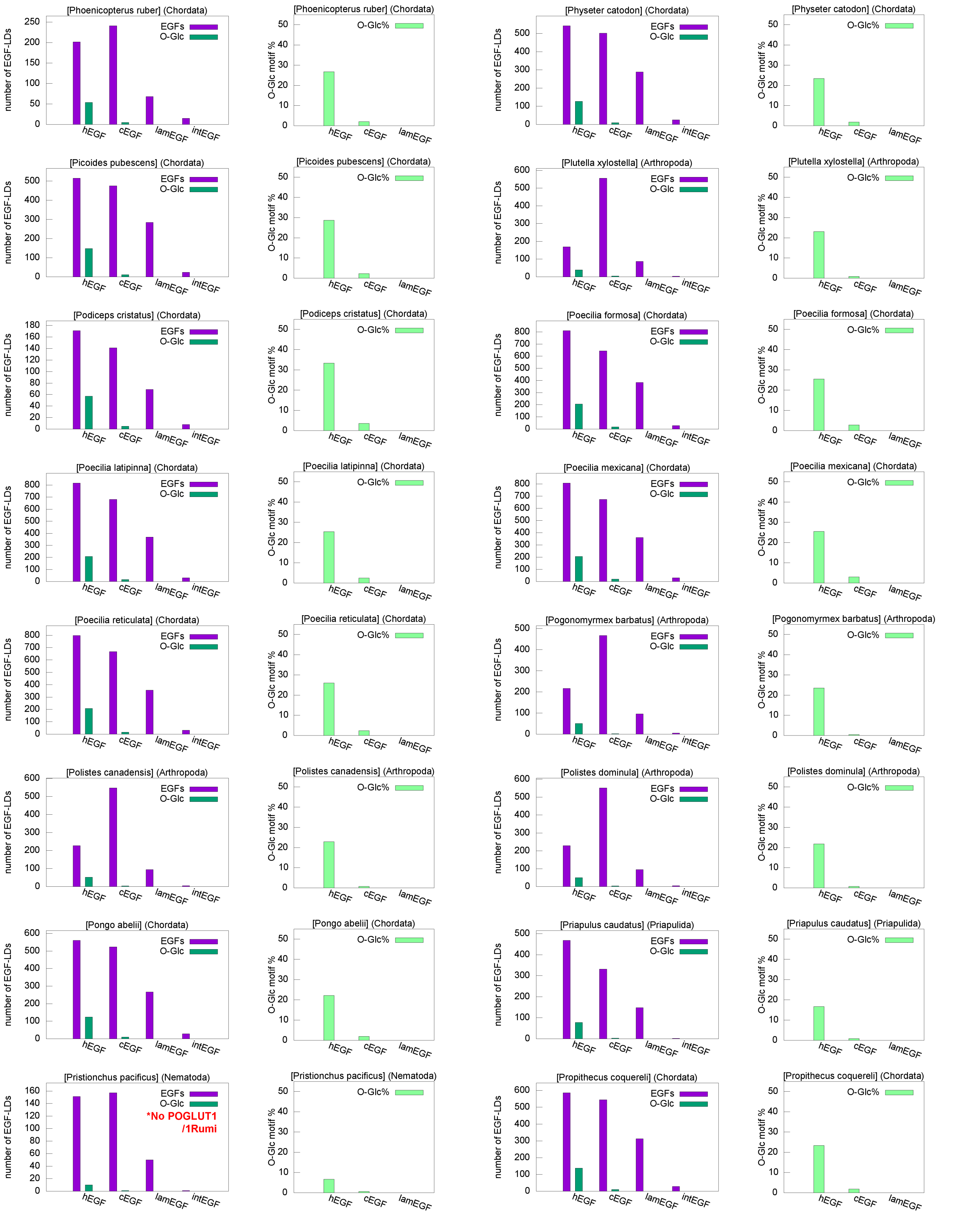
[Phoenicopterus ruber] (Chordata)
[Physeter catodon] (Chordata)
[Picoides pubescens] (Chordata)
[Plutella xylostella] (Arthropoda)
[Podiceps cristatus] (Chordata)
[Poecilia formosa] (Chordata)
[Poecilia latipinna] (Chordata)
[Poecilia mexicana] (Chordata)
[Poecilia reticulata] (Chordata)
[Pogonomyrmex barbatus] (Arthropoda)
[Polistes canadensis] (Arthropoda)
[Polistes dominula] (Arthropoda)
[Pongo abelii] (Chordata)
[Priapulus caudatus] (Priapulida)
[Pristionchus pacificus] (Nematoda)
[Propithecus coquereli] (Chordata)
number of EGF-LDs
O-Glc motif %
EGFs
O-Glc
O-Glc%
hEGF
cEGF
lamEGF
intEGF
*No POGLUT1
/1Rumi

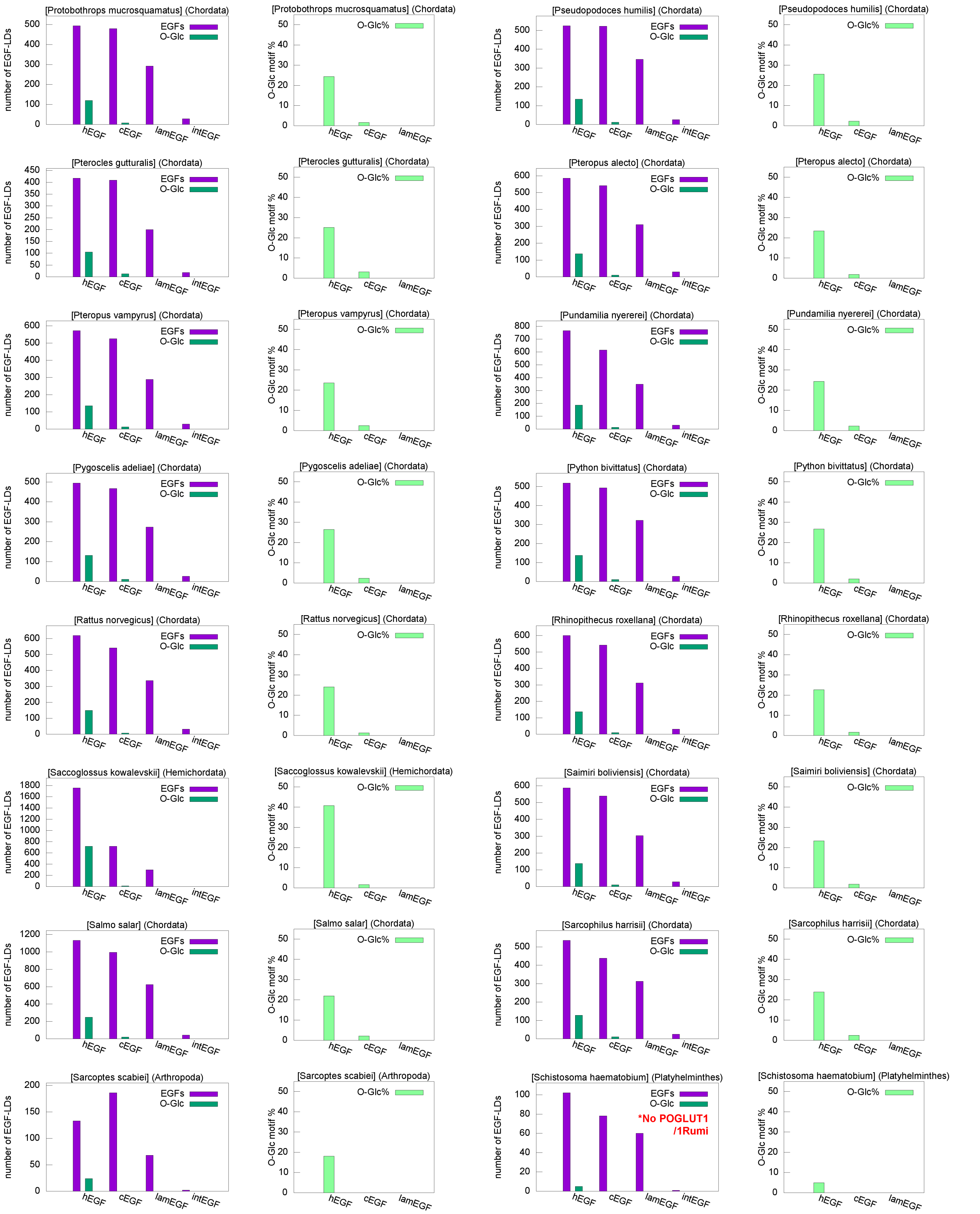
[Protobothrops mucrosquamatus] (Chordata)
[Protobothrops mucrosquamatus] (Chordata)
[Pseudopodoces humilis] (Chordata)
[Pseudopodoces humilis] (Chordata)
[Pterocles gutturalis] (Chordata)
[Pterocles gutturalis] (Chordata)
[Pteropus alecto] (Chordata)
[Pteropus alecto] (Chordata)
[Pteropus vampyrus] (Chordata)
[Pteropus vampyrus] (Chordata)
[Pundamilia nyererei] (Chordata)
[Pundamilia nyererei] (Chordata)
[Pygoscelis adeliae] (Chordata)
[Pygoscelis adeliae] (Chordata)
[Python bivittatus] (Chordata)
[Python bivittatus] (Chordata)
[Rattus norvegicus] (Chordata)
[Rattus norvegicus] (Chordata)
[Rhinopithecus roxellana] (Chordata)
[Rhinopithecus roxellana] (Chordata)
[Saccoglossus kowalevskii] (Hemichordata)
[Saccoglossus kowalevskii] (Hemichordata)
[Saimiri boliviensis] (Chordata)
[Saimiri boliviensis] (Chordata)
[Salmo salar] (Chordata)
[Salmo salar] (Chordata)
[Sarcophilus harrisii] (Chordata)
[Sarcophilus harrisii] (Chordata)
[Sarcoptes scabiei] (Arthropoda)
[Sarcoptes scabiei] (Arthropoda)
[Schistosoma haematobium] (Platyhelminthes)
[Schistosoma haematobium] (Platyhelminthes)
number of EGF-LDs
O-Glc motif %
EGFs
O-Glc
O-Glc%
hEGF
cEGF
lamEGF
intEGF
*No POGLUT1
/1Rumi

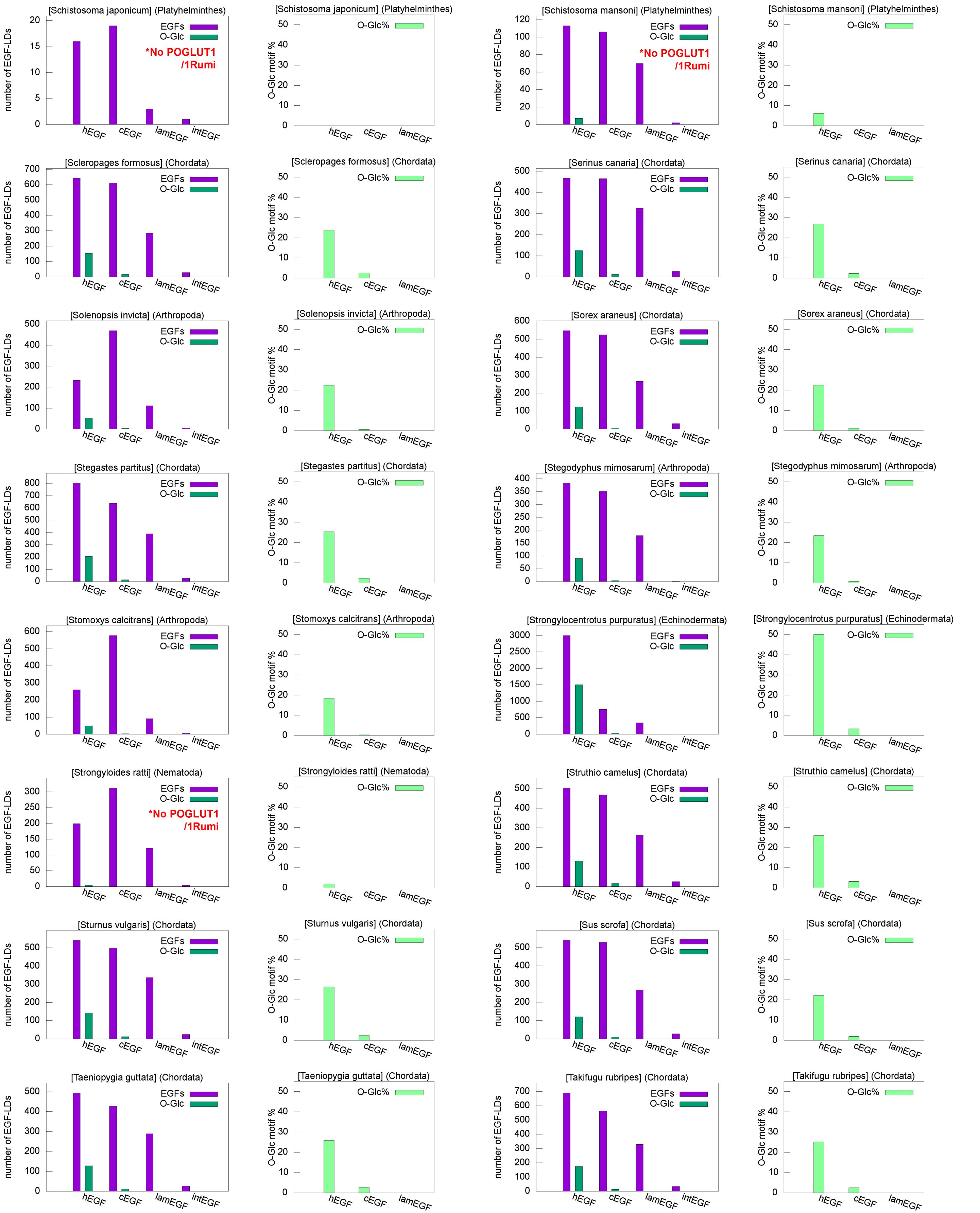
[Schistosoma japonicum] (Platyhelminthes)
*No POGLUT1 /1Rumi
[Schistosoma mansoni] (Platyhelminthes)
*No POGLUT1 /1Rumi
[Scleropages formosus] (Chordata)
[Serinus canaria] (Chordata)
[Solenopsis invicta] (Arthropoda)
[Sorex araneus] (Chordata)
[Stegastes partitus] (Chordata)
[Stegodyphus mimosarum] (Arthropoda)
[Stomoxys calcitrans] (Arthropoda)
[Strongylocentrotus purpuratus] (Echinodermata)
[Strongyloides ratti] (Nematoda)
*No POGLUT1 /1Rumi
[Struthio camelus] (Chordata)
[Sturnus vulgaris] (Chordata)
[Sus scrofa] (Chordata)
[Taeniopygia guttata] (Chordata)
[Takifugu rubripes] (Chordata)
number of EGF-LDs
O-Glc motif %
EGFs
O-Glc
O-Glc%
hEGF
cEGF
lamEGF
intEGF

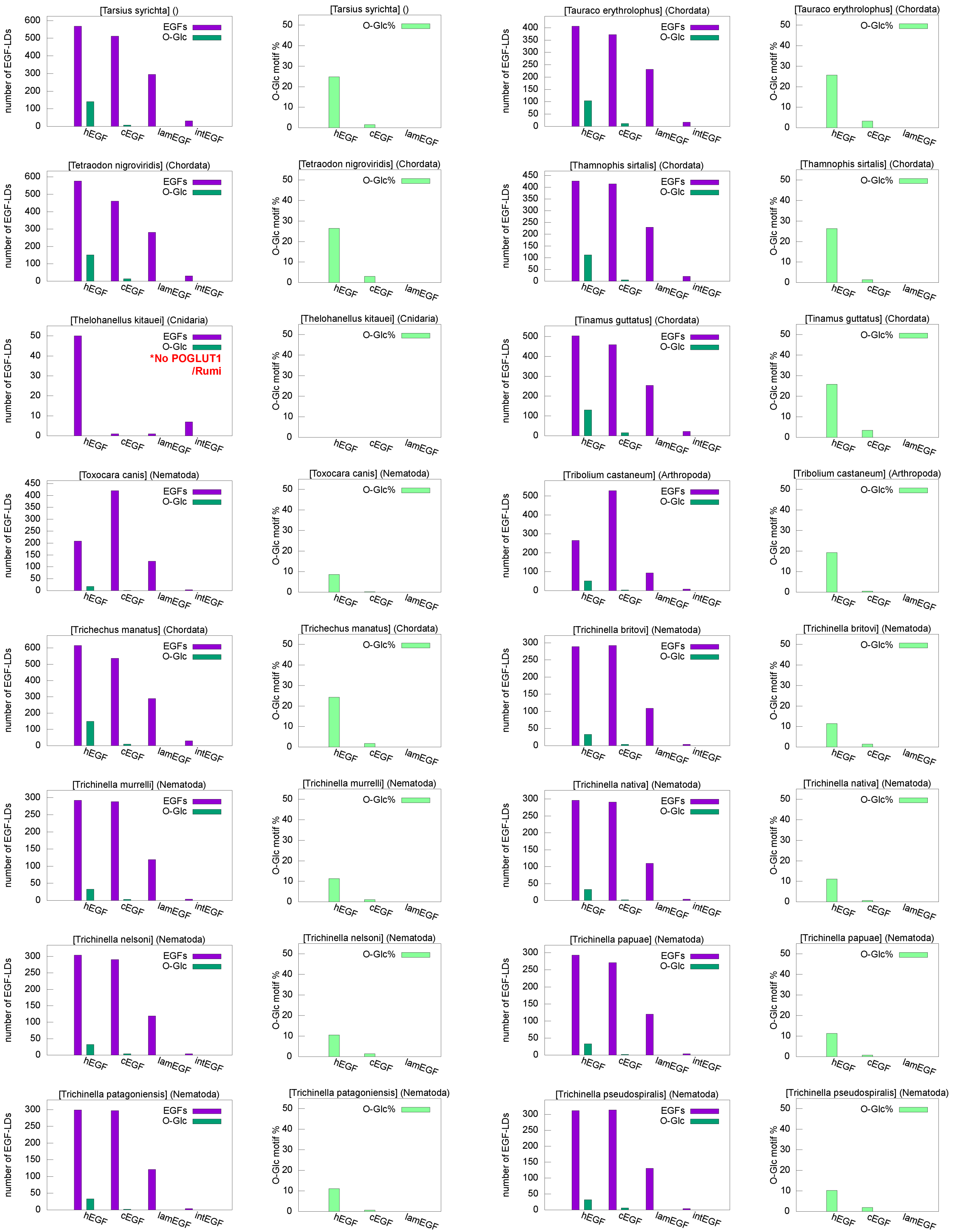

[Tarsius syrichta] ()
number of EGF-LDs
EGFs
O-Glc
hEGF cEGF lamEGF intEGF
[Tarsius syrichta] ()
O-Glc motif %
O-Glc%
hEGF cEGF lamEGF
[Tauraco erythrolophus] (Chordata)
number of EGF-LDs
EGFs
O-Glc
hEGF cEGF lamEGF intEGF
[Tauraco erythrolophus] (Chordata)
O-Glc motif %
O-Glc%
hEGF cEGF lamEGF
[Tetraodon nigroviridis] (Chordata)
number of EGF-LDs
EGFs
O-Glc
hEGF cEGF lamEGF intEGF
[Tetraodon nigroviridis] (Chordata)
O-Glc motif %
O-Glc%
hEGF cEGF lamEGF
[Thamnophis sirtalis] (Chordata)
number of EGF-LDs
EGFs
O-Glc
hEGF cEGF lamEGF intEGF
[Thamnophis sirtalis] (Chordata)
O-Glc motif %
O-Glc%
hEGF cEGF lamEGF
[Thelohanellus kitauei] (Cnidaria)
number of EGF-LDs
EGFs
O-Glc
*No POGLUT1
/Rumi
hEGF cEGF lamEGF intEGF
[Thelohanellus kitauei] (Cnidaria)
O-Glc motif %
O-Glc%
hEGF cEGF lamEGF
[Tinamus guttatus] (Chordata)
number of EGF-LDs
EGFs
O-Glc
hEGF cEGF lamEGF intEGF
[Tinamus guttatus] (Chordata)
O-Glc motif %
O-Glc%
hEGF cEGF lamEGF
[Toxocara canis] (Nematoda)
number of EGF-LDs
EGFs
O-Glc
hEGF cEGF lamEGF intEGF
[Toxocara canis] (Nematoda)
O-Glc motif %
O-Glc%
hEGF cEGF lamEGF
[Tribolium castaneum] (Arthropoda)
number of EGF-LDs
EGFs
O-Glc
hEGF cEGF lamEGF intEGF
[Tribolium castaneum] (Arthropoda)
O-Glc motif %
O-Glc%
hEGF cEGF lamEGF
[Trichechus manatus] (Chordata)
number of EGF-LDs
EGFs
O-Glc
hEGF cEGF lamEGF intEGF
[Trichechus manatus] (Chordata)
O-Glc motif %
O-Glc%
hEGF cEGF lamEGF
[Trichinella britovi] (Nematoda)
number of EGF-LDs
EGFs
O-Glc
hEGF cEGF lamEGF intEGF
[Trichinella britovi] (Nematoda)
O-Glc motif %
O-Glc%
hEGF cEGF lamEGF
[Trichinella murrelli] (Nematoda)
number of EGF-LDs
EGFs
O-Glc
hEGF cEGF lamEGF intEGF
[Trichinella murrelli] (Nematoda)
O-Glc motif %
O-Glc%
hEGF cEGF lamEGF
[Trichinella nativa] (Nematoda)
number of EGF-LDs
EGFs
O-Glc
hEGF cEGF lamEGF intEGF
[Trichinella nativa] (Nematoda)
O-Glc motif %
O-Glc%
hEGF cEGF lamEGF
[Trichinella nelsoni] (Nematoda)
number of EGF-LDs
EGFs
O-Glc
hEGF cEGF lamEGF intEGF
[Trichinella nelsoni] (Nematoda)
O-Glc motif %
O-Glc%
hEGF cEGF lamEGF
[Trichinella papuae] (Nematoda)
number of EGF-LDs
EGFs
O-Glc
hEGF cEGF lamEGF intEGF
[Trichinella papuae] (Nematoda)
O-Glc motif %
O-Glc%
hEGF cEGF lamEGF
[Trichinella patagoniensis] (Nematoda)
number of EGF-LDs
EGFs
O-Glc
hEGF cEGF lamEGF intEGF
[Trichinella patagoniensis] (Nematoda)
O-Glc motif %
O-Glc%
hEGF cEGF lamEGF
[Trichinella pseudospiralis] (Nematoda)
number of EGF-LDs
EGFs
O-Glc
hEGF cEGF lamEGF intEGF
[Trichinella pseudospiralis] (Nematoda)
O-Glc motif %
O-Glc%
hEGF cEGF lamEGF

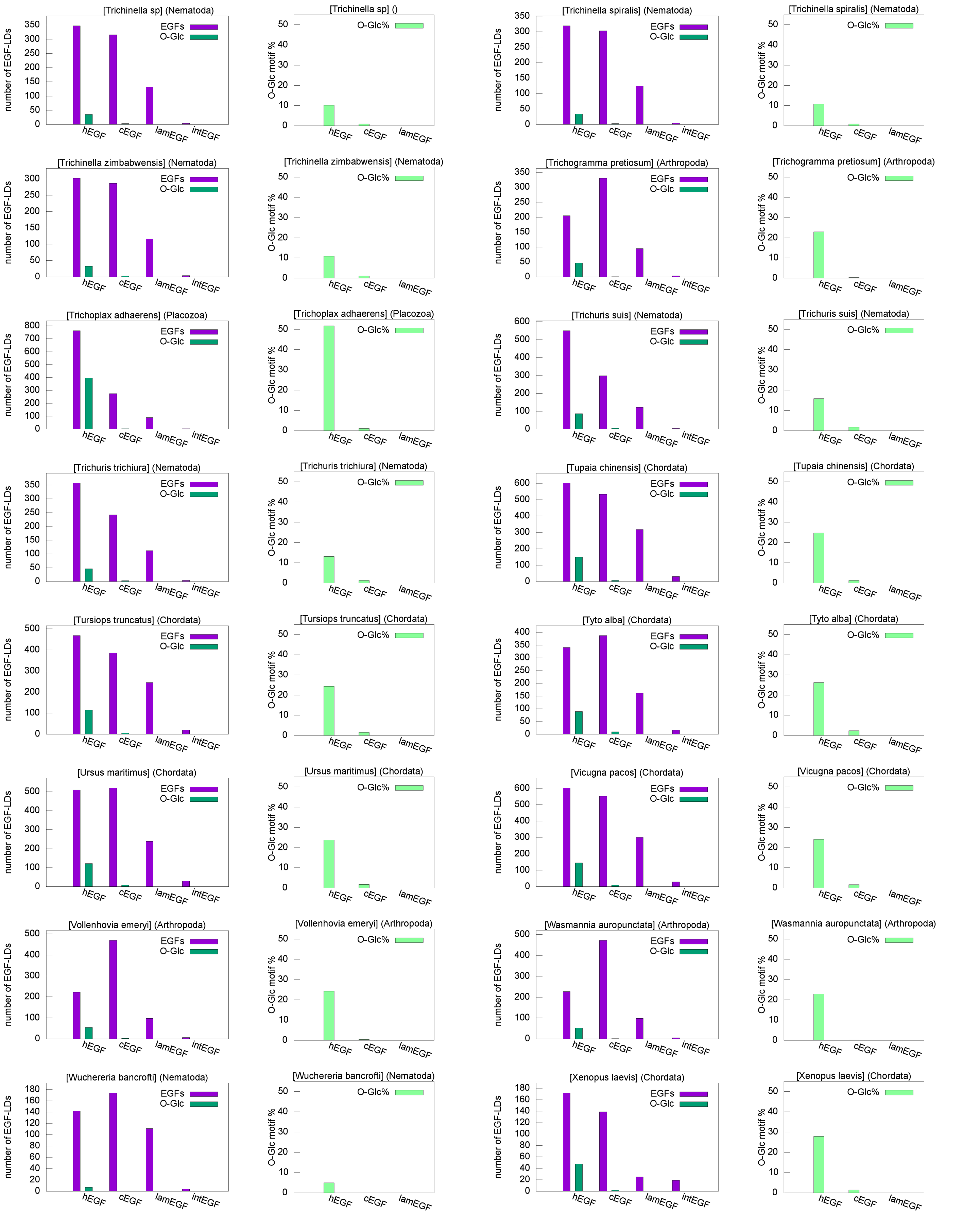
[Trichinella sp] (Nematoda)
[Trichinella sp] ()
[Trichinella spiralis] (Nematoda)
[Trichinella spiralis] (Nematoda)
[Trichinella zimbabwensis] (Nematoda)
[Trichinella zimbabwensis] (Nematoda)
[Trichogramma pretiosum] (Arthropoda)
[Trichogramma pretiosum] (Arthropoda)
[Trichoplax adhaerens] (Placozoa)
[Trichoplax adhaerens] (Placozoa)
[Trichuris suis] (Nematoda)
[Trichuris suis] (Nematoda)
[Trichuris trichiura] (Nematoda)
[Trichuris trichiura] (Nematoda)
[Tupaia chinensis] (Chordata)
[Tupaia chinensis] (Chordata)
[Tursiops truncatus] (Chordata)
[Tursiops truncatus] (Chordata)
[Tyto alba] (Chordata)
[Tyto alba] (Chordata)
[Ursus maritimus] (Chordata)
[Ursus maritimus] (Chordata)
[Vicugna pacos] (Chordata)
[Vicugna pacos] (Chordata)
[Vollenhovia emeryi] (Arthropoda)
[Vollenhovia emeryi] (Arthropoda)
[Wasmannia auropunctata] (Arthropoda)
[Wasmannia auropunctata] (Arthropoda)
[Wuchereria bancrofti] (Nematoda)
[Wuchereria bancrofti] (Nematoda)
[Xenopus laevis] (Chordata)
[Xenopus laevis] (Chordata)
number of EGF-LDs
O-Glc motif %
EGFs
O-Glc
O-Glc%
hEGF
cEGF
lamEGF
intEGF

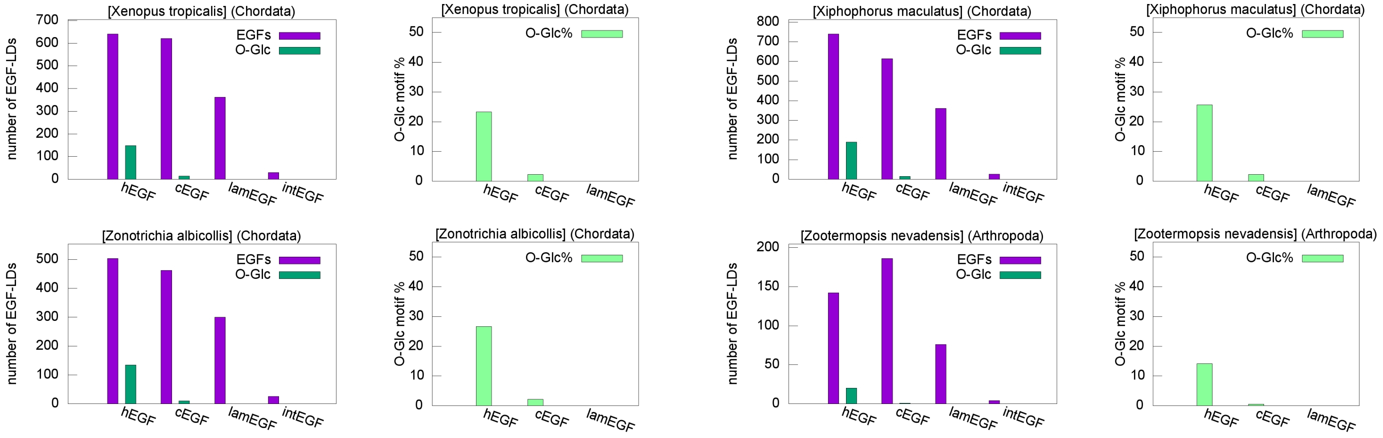
[Xenopus tropicalis] (Chordata)
number of EGF-LDs
EGFs
O-Glc
hEGF
cEGF
lamEGF
intEGF
O-Glc motif %
O-Glc%
[Xiphophorus maculatus] (Chordata)
[Zonotrichia albicollis] (Chordata)
[Zootermopsis nevadensis] (Arthropoda)
