# Supplementary material for: Structural basis of Notch O-glucosylation and O–xylosylation by mammalian protein–O-glucosyltransferase 1 (POGLUT1)
Source: Nat Commun. 2017 Aug 4;8:185. doi: 10.1038/s41467-017-00255-7 (PMC5543122; doi:10.1038/s41467-017-00255-7)
Supplement: Supplementary file 4 — Reviewer reports and authors' response from the peer review of this Article at Nature Communications [file 41467_2017_255_MOESM4_ESM.pdf]

PEER REVIEW FILE

<u>Reviewers' Comments</u>:

Reviewer #1 (Remarks to the Author):

We reviewed this manuscript and the letter discussing how this Li et al manuscript advances the field in light of the Yu et al paper published in Nature Chemical Biology. We also reviewed the publishsed Yu et al paper as asked by the editor.

Both manuscripts report the structure of the sugar transferase POGLUT1. The Nature Chemical Biology Yu et al from Drosophila and the current Li et al manuscript from Human. Both report complexes with an EGF domain. Both provide ternary complexes of UDP and the sugar modified EGF. The current Li et al manuscript has the 2F sugar observed on the EGF and the Yu et al paper have regular glucose on the EGF. The current manuscript also uses a substrate mimic to get a ternary substrate complex. This last point seems the main difference between these two works.

The protein in the ternary substrate complex has a mildly different conformation in the position of some secondary structure features. The changes appear modest but the Li et al structure is more accurate in presenting a ternary substrate complex. Because of these differences the authors gain some additional insight into why the enzyme may not be able to have a threonine residue on the EGF be glycosylated.

Li et al also discuss the dual specificity of POGLUT1 seen in enzyme assays. But is this relevant for the biology since it does not seem this enzyme transfers xylose within nature. The docking model of the UDP-xylose complex is based on the UDP POGLUT1 complex so it not a surprise that it looks different from the UDP-glucose conformation seen using the inactive substrate analogue. The model seems a bit biased.

The work is sound and well performed. The Yu et al paper in Nature Chemical Biology is an interesting but specialized advance. After review I believe the current manuscript makes some improvements in knowledge but it does not represent a major advance over the Yu et al paper. The advances are in this Li et al manuscript depend on the better ternary substrate complex and focus on the details of the enzyme reaction processes.

If the journal wants to consider a revised version the authors should expand the discussion section. They should discuss the meaning of the dual specificity. They should focus on the new

elements of this manuscript. They should improve the modeling work on the UDP-xylose. Probably comparing their work to the Yu et al paper would be useful and citing this work would be needed. The abstract would need to be focused on the new details.

Reviewer #2 (Remarks to the Author):

Li and co-workers report crystal structures of the human protein O-glucosyltransferase 1 (POGLUT1), an enzyme that transfers a glucose/xylose moiety from UDP-Glc/UDP-Xyl to a serine residue present in epidermal growth factor-like (EGF) repeats. Specifically, the authors solved five crystal structures of POGLUT1 in complex with (i) UDP/EGF12 at 1.5A, (ii) UDP/F7EGF1 at 1.45A, (iii) UDP/EGF(+) at 1.43A, (iv) UDP-CH2-Glc/EGF(+) at 1.8A and (v) UDP-2F-Glc/EGF(+) at 1.31A. Based on the structural data a molecular mechanism for substrate binding/specificity and catalysis is proposed.

The current studies on the human POGLUT1 could be a very good complement of the recent studies on the Drosophila homologue RUMI, “Structural analysis of Notch-regulating Rumi reveals basis for pathogenic mutations.” by Yu and co-workers published in Nat. Chem. Biol. - doi: 10.1038/nchembio.2135. Specifically, regarding (i) the mechanistic explanation for the dual donor substrate specificity and (ii) identification of a common determinant for the POGLUT1 and POFUT1 acceptor substrate recognition.

Some questions/suggestions to be addressed are listed below:

1. The manuscript should be vastly extended (main text limit - not including abstract, Methods, References and figure legends - is 5,000 words/up to 10 displayed figures/tables) including the current structural/biochemical knowledge on related glycosyltransferases (e.g. RUMI, POFUT1).

- A scheme of the reaction catalyzed by POGLUT1 should be included in the main text.

2. Overall structure of POGLUT1

- The complete amino acid sequence of the truncated POGLUT1 construct should be included as a Supplementary Figure.

- The electron density maps (2mFo-DFc) for all ligands observed in the five crystal structures as should be included as a Supplementary Figure.

- A DALI report showing similarity with related glycosyltransferases should be included as Supplemental Information.

- A structural alignment with related glycosyltransferases should be included as a Figure in the main text – highlighting key residues involved in substrate recognition and catalysis. Please also state pdb code/biological function/r.m.s.d. values.

3. POGLUT1-EGF-UDP complexes

- A structural superimposition of different EGF ligands observed in the three POGLUT1-EGF-UDP complexes should be performed and included as part of a main Figure (including r.m.s.d. values).

- A model for EGF recognition should be discuss in detail, considering all available biochemical and structural data.

- What is the conformation of the EGF ligands observed in the POGLUT1-EGF-UDP, when compared with the RUMI-EGF and RUMI-EGF-UDP complexes, and other related glycosyltransferases.

4. POGLUT1-UDP-CH2-GLC and POGLUT1-UDP-2F-GLC complexes

- A scheme of the catalytic mechanism proposed for POGLUT1 should be included in the main text.

- A POGLUT1-UDP-CH2-XYL complex should undoubtedly contribute to demonstrate the proposed model for dual donor substrate specificity mechanism suggested by the authors. MAJOR.

- Additional experimental data supporting this model, and that of the specificity for Ser in the EGF acceptor substrates, should be provided in the form of biochemical/biophysical data. MAJOR.

5. Methods

- Expression and Purification of POGLUT1 and the EGF-like domains. Both protocols should be vastly extended/carefully explained in detail (e.g. including growth medium/buffer compositions used in each step/EndoH deglycosylation conditions/storage conditions).

- “The structure of the POGLUT1-EGF complex was solved by the single wavelength anomalous

dispersion (SAD) technique using POGLUT1/EGF(+)/UDP crystals grown in the presence of 0.5 M potassium iodide. The diffraction data were collected at 1.77Å on beamline 08ID-1 at the Canadian Light Source." Please include data collection and experimental phasing data in Table S1.

Reviewer #3 (Remarks to the Author):

POGLUT1 is one of Notch-modifying enzymes that is critical for Notch signaling pathway. It has been shown that POGLUT1 display dual glycosyltransferase activity: O-glucosyltransferase and O-xylosyltransferase activity. In this study, Li et al. performed the structural analysis for human POGLUT1. Although similar structural analysis was recently reported by another group (Yu et al. Nat. Chem. Biol. 2016), this manuscript reported the new aspects of the structures of POGLUT1 in complex with EGF domain and donor analogues, which is different from those reported by the other group. The newly obtained structure led authors to propose the model how POGUT1 catalyzes the transfer of both glucose and xylose.

1. Although this manuscript focused on explaining the dual donor substrate specificity for POGLUT1, the biological significance of xylosyltransferase activity has not been provided. Lack of biological significance of Notch O-xylosylation makes the proposed model for UDP-xylose complex less significant. I understand that addressing the biological function of O-xylose is beyond the scope of this manuscript. However, authors should justify why providing a mechanistic explanation for dual donor substrate specificity would be important.
2. It has been reported that O-xylosylation by POGLUT1 requires a diserine motif, CXSSPC (Takeuchi H et al. PNAS 2011). Does the UDP-xylose ternary complex provide insights why the diserine motif is important for O-xylosylation?
3. Authors proposed POGLUT1 recognizes the structure in 5-resudue C2C3 loop. Although it is quit interesting, the supporting data are limited to the sequence analysis (SI Figure 1). It would be more convincing if authors could provide biochemical data by altering the number of residues in C2C3 loop of certain EGF domains such as EGF12.
4. In Figure 3G, it would be more comparable if authors show the UDP-2F-glucose Michaelis complex instead of the UDP-CH2-glucose complex because UDP-2F-glucose behaves as a natural POGLUT1 donor substrate.
5. What does EGF(+) stand for ? (eg. line 109)
6. I found that the supplemental document explaining the difference between this manuscript and the one by Yu et al. is quite important to understand the importance of this manuscript. These information should be provided in the discussion.

Response to Reviewers' Comments

Reviewer #1 (Remarks to the Author):

We reviewed this manuscript and the letter discussing how this Li et al manuscript advances the field in light of the Yu et al paper published in Nature Chemical Biology. We also reviewed the published Yu et al paper as asked by the editor.

Both manuscripts report the structure of the sugar transferase POGLUT1. The Nature Chemical Biology Yu et al from Drosophila and the current Li et al manuscript from Human. Both report complexes with an EGF domain. Both provide ternary complexes of UDP and the sugar modified EGF. The current Li et al manuscript has the 2F sugar observed on the EGF and the Yu et al paper have regular glucose on the EGF. The current manuscript also uses a substrate mimic to get a ternary substrate complex. This last point seems the main difference between these two works.

R1.1a) In addition to the UDP-CH_2-glucose complex, we would like to point out that our paper also included complexes with three different EGF-like domains, data that uniquely allowed us to establish the conserved determinants of recognition used by POGLUT1.

R1.1b) In our revised paper, we now report a new ternary complex that validates our conclusion that the serine specificity shown by POGLUT1 is a result of mainchain-sidechain constraints (contrary to that reported by Yu *et al*). In addition, we provide new kinetic and mutagenesis data, as well as large-scale protein sequence database analysis from all 339 genome-sequenced animals. Together these data have significantly advanced our understanding of each of the following: i) the substrate requirements for xylosylation, ii) the role played by the C^2C^3 loop (the site of fucosylation by POFUT1) for both POGLUT1 and dRUMI and iii) the co-evolution of POGUT1 and its EGF-like domain substrates in animals. Finally, we would like to point out that we now have a manuscript *in press* that describes the first structures of POFUT1 in complex with its EGF-like domain substrates (*Nature Chemical Biology*, online release May 22, 2017). Reference to this work has allowed us to discuss the shared basis for how these enzymes recognize their diverse, folded EGF-like domain substrates and to provide perspective on the role played by POGLUT1, POFUT1 and EGF-like domains of the h-type in animal evolution. Taken together, we believe that our revised POGLUT1 manuscript now represents a very significant advance vis-à-vis the Yu *et al*. paper.

R1.1c) In summary, each of the following represents new data that has been added to our revised manuscript:

i) The X-ray structure of a ternary complex of a threonine mutant of hEGF12 at the O-glucose site (S458T) validating our conclusion that mainchain-sidechain interactions are the basis for POGLUT1/dRUMI serine specificity.

ii) Mutagenesis and enzyme kinetic data on hEGF12 using both hPOGLUT1 and dRUMI establishing that the C^2C^3-loop of the EGF-like domain is a determinant of recognition by both enzymes.

iii) Mutagenesis and enzyme kinetic data on hEGF12 showing that POGLUT1 does not possess a requirement for a diserine-containing glycosylation motif as was previously reported.

iv) The addition of docking and energy minimization calculations that optimize and validate our UDP-xylose docking results.

v) Analysis of the NCBI BLAST nonredundant (nr) protein sequence database (covering all 339 genome-sequenced animals) showing the co-evolution of POGLUT1 and its EGF-like domain substrates. With reference to our work on POFUT1 (*in press*) the analysis establishes that the presence of POFUT1, POGLUT1 and the EGF-like domains of the h-type are closely linked to animal evolution.

R1.1d) The new data can be found in the following Tables and Figures:

i) Enzyme kinetic analysis of hPOGLUT1 and dRUMI with EGF-like domains (Table 1 and SI figure 9). These data pertain to the preference for a 5-residue C^2C^3 loop (by both POGLUT1 and dRUMI), the lack of a dependence on a diserine-containing glycosylation motif for xylosylation (K_M and V_{max}), and the serine-specificity shown by POGLUT1.

ii) Mass-spectrometry data confirming that both EGF(+) and EGF12 are xylosylated *in vitro* (Figure 6b,c and SI Figure 10).

iii) Database mining showing the correlation between the presence of POGLUT1, a high O-glucosylation motif frequency, and h-type EGF-like domains (SI Tables 5 and 6, SI Figure 2, SI Dataset 1).

iv) Optimization of the UDP-xylose docking model (SI Figure 8).

v) A new ternary complex showing the effect of a threonine residue at the site of O-glucosylation (Figure 5c, SI Table 1).

vi) The new information requested by the reviewers (eg. Dali search, PDB comparison, composite omit maps, etc.) has been incorporated at various locations in the paper.

R1.1e) For comparison, our paper includes data and discussion on the following topics that are not included in the Yu *et al* paper:

i) X-ray structures of ternary complexes containing three different EGF-like domains that together provide insight into the common determinants of EGF-like domain recognition. These domains include examples of EGF-like domains with and without diserine-containing glycosylation motifs.

ii) A ternary complex containing a substrate analogue (UDP-CH_2-glucose) providing a model of the POGLUT1 Michaelis complex for glucosylation.

iii) A demonstration that the serine specificity shown by POGLUT1/dRUMI is the result of sidechain-mainchain constraints (with supporting crystallographic confirmation using a threonine mutant).

iv) A demonstration that POGLUT1 can access two local conformational states as well as docking experiments suggesting that these states are individually responsible for the binding/reaction of UDP-glucose and UDP-xylose.

v) The role of the C^2C^3 loop (the site of POFUT1 O-fucosylation) as a determinant of POGUT1/dRUMI substrate recognition.

vi) A demonstration that a diserine-containing glycosylation motif (22% of all POGLUT1 substrates) is not required for O-xylosylation (enzyme kinetic and mutagenesis data) and that at least an extra 36% of POGLUT1 substrates (those with the C^1XSNPC^2 motif) have the potential to serve as O-xylosylation substrates.

vii) Database analysis providing insights into the co-evolution of POGLUT1 and its EGF-like domain substrates.

viii) Insights into catalytic mechanism: i) the role of loops in sealing the reaction centre and ii) the possible role played by intra-domain protein-carbohydrate interactions in promoting the release of the glycosylated EGF-like domain

The protein in the ternary substrate complex has a mildly different conformation in the position of some secondary structure features. The changes appear modest but the Li et al structure is more accurate in presenting a ternary substrate complex. Because of these differences the authors gain some additional insight into why the enzyme may not be able to have a threonine residue on the EGF be glycosylated.

R1.2) With regard to the serine specificity shown by POGLUT1/dRUMI (inability to use threonine) we argued that this arises from sidechain-mainchain constraints that do not allow threonine to access the rotamer required for activation (by the catalytic base) and in-line S_N2 attack on C1 of the donor substrate. Yu *et al* suggest that threonine cannot be used because the threonine methyl group would clash with the saccharide moiety of the donor substrate.

In support of our conclusion, we now provide energy calculations showing that serine, but not threonine, can access the sidechain rotamer required for catalysis. In addition, we report a ternary complex with a mutant form of human Notch EGF12 (S458T) which introduces a threonine residue at the site of glycosylation. The complex shows that the threonine sidechain populates a rotamer that does not position the hydroxyl group for activation or nucleophilic attack, strong support of our explanation. Contrary to that suggested by Yu *et al*, our structures also show that the threonine methyl group would not sterically clash with the donor substrate in any orientation.

R1.3) With regard to the complexes that we have reported, all of them are ternary complexes involving either UDP or UDP-CH_2-glucose (we also have a UDP complex where 2F-glucose was transferred to the EGF-like domain in the crystal). The two local POGLUT1 conformations that we observe (one for the

UDP-containing complexes and one for the UDP-CH_2-glucose-containing complex) are relevant to our discussion of the binding/reaction of UDP-glucose and UDP-xylose, as discussed below (R1.5).

Li et al also discuss the dual specificity of POGLUT1 seen in enzyme assays. But is this relevant for the biology since it does not seem this enzyme transfers xylose within nature. The docking model of the UDP-xylose complex is based on the UDP POGLUT1 complex so it not a surprise that it looks different from the UDP-glucose conformation seen using the inactive substrate analogue. The model seems a bit biased.

R1.4) Takeuchi *et al.* (2011 PNAS, 108, 16600–16605) have reported that the Notch2 protein is xylosylated in HEK293T cells and, moreover, that efficient xylosylation was dependent on a diserine-containing glycosylation motif. Although the biological relevance of O-xylosylation has not been established, we have now provided data showing that xylosylation does not require a diserine and that the EGF-like domains containing the C^1XSNPC^2 motif found in 36% of POGLUT1 substrates can be xylosylated (the diserine motif containing ones account for another 22%). Results of this nature will hopefully stimulate new research aimed at determining the extent to which proteins like Notch are xylosylated *in vivo* and, in turn, the role that xylosylation might play as outlined in the revised discussion. That a diserine-containing glycosylation motif is not required for xylosylation is consistent with the fact that the "second" serine does not interact with the donor substrate.

R1.5) All of our complexes are of high resolution (1.3 - 2.1 Å resolution) and the difference in conformation observed is well defined. Moreover, the difference is clearly the result of the glucose moiety in the UDP-CH_2-glucose complex which leads to an increase in the volume of the glucose/xylose subsite relative to that seen in the UDP complexes. Indeed, our models are "biased" toward the starting structure because POGLUT1 is treated as a rigid body in the docking procedure. Only the torsion angles of the UDP-xylose molecule were allowed to vary in the work presented in our initial submission.

The impetus for this docking work is the fact that the hydroxymethyl group of glucose is found to be buried in the UDP-CH_2-glucose complex where it makes key interactions with POGLUT1. The loss of these interactions and the cavity that would be created with xylose would be destabilizing absent compensatory conformational changes in POGLUT1. Our docking experiments show that POGLUT1 in the UDP-bound conformation provides a conformation that can bind UDP-xylose. The strength of our model for UDP-xylose binding is that it is based on one of the two local conformations that we observe in our high resolution x-ray structures.

To address the concerns expressed, we have performed two new types of calculation to further validate the suggestion that POGLUT1 binds UDP-xylose in its UDP-bound conformation. Firstly, we have repeated the UDP-xylose docking experiments using the Autodock and Autodock Vina packages, which allowed protein sidechain flexibility. Secondly, we've now used the Rosetta suite to refine the docking solutions. The Rosetta suite allows all-atom structural optimization. We find that when UDP-xylose is docked in the UDP-bound conformation of POGLUT1, only small changes in the structure are observed after Rosetta optimization. In contrast, Rosetta optimization of the complex obtained with UDP-xylose docked to POGLUT1 in the UDP-CH_2-glucose-bound conformation led to significant movement that leads to a reduction in the volume of the subsite to maximize the interaction with the xylose moiety. These

results strongly support the suggestion that UDP-xylose would not form a stable complex when bound to POGLUT1 in its UDP-CH_2-glucose conformation and that UDP-xylose likely interacts with POGLUT1 in its UDP-bound conformation.

The work is sound and well performed. The Yu et al paper in Nature Chemical Biology is an interesting but specialized advance. After review I believe the current manuscript makes some improvements in knowledge but it does not represent a major advance over the Yu et al paper. The advances are in this Li et al manuscript depend on the better ternary substrate complex and focus on the details of the enzyme reaction processes.

See R1.1) above

If the journal wants to consider a revised version the authors should expand the discussion section. They should discuss the meaning of the dual specificity. They should focus on the new elements of this manuscript. They should improve the modeling work on the UDP-xylose. Probably comparing their work to the Yu et al paper would be useful and citing this work would be needed. The abstract would need to be focused on the new details.

R1.6) As suggested, we have greatly expanded the results and discussion sections with reference to all of these points as discussed above. Moreover, reference to our own POFUT1 work (*in press*) and new sequence database mining pertaining to POGLUT1 and its substrates has also added much scope to our revised manuscript (see R1.1).

Reviewer #2 (Remarks to the Author):

Li and co-workers report crystal structures of the human protein O-glucosyltransferase 1 (POGLUT1), an enzyme that transfers a glucose/xylose moiety from UDP-Glc/UDP-Xyl to a serine residue present in epidermal growth factor-like (EGF) repeats. Specifically, the authors solved five crystal structures of POGLUT1 in complex with (i) UDP/EGF12 at 1.5A, (ii) UDP/F7EGF1 at 1.45A, (iii) UDP/EGF(+) at 1.43A, (iv) UDP-CH2-Glc/EGF(+) at 1.8A and (v) UDP-2F-Glc/EGF(+) at 1.31A. Based on the structural data a molecular mechanism for substrate binding/specificity and catalysis is proposed.

The current studies on the human POGLUT1 could be a very good complement of the recent studies on the Drosophila homologue RUMI, “Structural analysis of Notch-regulating Rumi reveals basis for pathogenic mutations.” by Yu and co-workers published in Nat. Chem. Biol. - doi: 10.1038/nchembio.2135. Specifically, regarding (i) the mechanistic explanation for the dual donor substrate specificity and (ii) identification of a common determinant for the POGLUT1 and POFUT1 acceptor substrate recognition.

Some questions/suggestions to be addressed are listed below:

1. The manuscript should be vastly extended (main text limit - not including abstract, Methods,

References and figure legends - is 5,000 words/up to 10 displayed figures/tables) including the current structural/biochemical knowledge on related glycosyltransferases (e.g. RUMI, POFUT1).

R2.1) Both the results and discussion sections have been expanded significantly as outlined in R.1.1 above.

- A scheme of the reaction catalyzed by POGLUT1 should be included in the main text.
R2.2) Done, see Figure 1a.

2. Overall structure of POGLUT1

- The complete amino acid sequence of the truncated POGLUT1 construct should be included as a Supplementary Figure.
R2.3) Done, see SI Figure 1.

- The electron density maps (2mFo-DFc) for all ligands observed in the five crystal structures as should be included as a Supplementary Figure.
R2.4) Done, see SI Figure 4

- A DALI report showing similarity with related glycosyltransferases should be included as Supplemental Information.
R2.5) Done, see SI Table 4

- A structural alignment with related glycosyltransferases should be included as a Figure in the main text
R2.6) Done, see SI Figure 7 (Dali hits)

– highlighting key residues involved in substrate recognition and catalysis. Please also state pdb code/biological function/r.m.s.d. values.

R2.7) With the exception of dRUMI, the structures identified in the Dali search all use different donor and acceptor substrates and employ different mechanisms (they include both inverting and retaining GTs). As such, a common set of key residues shared by these enzymes cannot be identified. We have listed their donor and acceptor substrates and their catalytic properties in SI Table 4.

3. POGLUT1-EGF-UDP complexes

- A structural superimposition of different EGF ligands observed in the three POGLUT1-EGF-UDP complexes should be performed and included as part of a main Figure (including r.m.s.d. values).

R2.8) The superimpositions are now shown in Figure 1c and SI Figure 5. The RMSD values are indicated in the legend to SI Figure 5. Comparison with all O-glucosylation motif-containing EGF-like domains found in the PDB (14 unique domains, 82 observations) is also provided in SI figure 5.

- A model for EGF recognition should be discuss in detail, considering all available biochemical and structural data.

R2.9) We have significantly expanded the section on EGF-like domain recognition in our results section. Reference to dRUMI (Yu *et al*) and the parallels with POFUT1 (our own work *in press*) are also now included.

- What is the conformation of the EGF ligands observed in the POGLUT1-EGF-UDP, when compared with the RUMI-EGF and RUMI-EGF-UDP complexes, and other related glycosyltransferases.

R2.10) The conformation of the EGF ligands are very similar to those observed in the dRUMI structure. Comparisons with the dRUMI structure is shown in SI Figure 6 and is discussed in the main text. As mentioned above, the parallels with POFUT1 are also now included.

4. POGLUT1-UDP-CH2-GLC and POGLUT1-UDP-2F-GLC complexes

- A scheme of the catalytic mechanism proposed for POGLUT1 should be included in the main text.
R2.11) Done, see Figure 3g.

- A POGLUT1-UDP-CH2-XYL complex should undoubtedly contribute to demonstrate the proposed model for dual donor substrate specificity mechanism suggested by the authors. MAJOR.
R2.12) The synthesis of UDP-CH_2-xylose would involve months and there is no guarantee that the compound can be successfully synthesized. As such, we do not see this as a feasible undertaking.

- Additional experimental data supporting this model, and that of the specificity for Ser in the EGF acceptor substrates, should be provided in the form of biochemical/biophysical data. MAJOR.
R2.13) Although not directly validating our UDP-xylose docking model, we have provided new kinetic data showing that POGLUT1 can xylosylate hF7EGF1, hEGF12 and a mutant of hEGF12 (N459S, introducing a diserine motif). The data show that POGLUT1 possesses similar K_M and V_{max} values for these acceptors for both the glucosylation and xylosylation reactions. We have suggested that UDP-xylose bound to POGLUT1 in the UDP-CH_2-conformation would be destabilized by the loss of interactions involving the hydroxymethyl group that is present in glucose; our Rosetta minimizations support this suggestion. It follows that a second conformation capable of using UDP-xylose with similar catalytic efficiency likely exists.

As discussed above (R1.1d, R1.2) we have now determined the structure of a new ternary complex of POGLUT1 with a mutant EGF-like domain (hEGF12:S458T, Figure 5c) that introduces a threonine at the site of glucosylation. The structure shows that the threonine hydroxyl group populates a rotamer that is

not activated by the POGLUT1 catalytic base and that is not positioned for in-line nucleophilic attack on C1 of the donor substrate. This result strongly supports our conclusion that the serine specificity shown by POGLUT1 arises from sidechain-mainchain constraints and, as such, is a direct outcome of POGLUT1's dependence on folded EGF-like domain substrates.

5. Methods

- Expression and Purification of POGLUT1 and the EGF-like domains. Both protocols should be vastly extended/carefully explained in detail (e.g. including growth medium/buffer compositions used in each step/EndoH deglycosylation conditions/storage conditions).

R2.14) We have expanded the methods section to include these details.

- "The structure of the POGLUT1-EGF complex was solved by the single wavelength anomalous dispersion (SAD) technique using POGLUT1/EGF(+)/UDP crystals grown in the presence of 0.5 M potassium iodide. The diffraction data were collected at 1.77Å on beamline 08ID-1 at the Canadian Light Source." Please include data collection and experimental phasing data in Table S1.

R2.15) We have added the data collection statistics for the SAD dataset (see SI table 1). The experimental phasing metrics are now provided in the methods section.

Reviewer #3 (Remarks to the Author):

POGLUT1 is one of Notch-modifying enzymes that is critical for Notch signaling pathway. It has been shown that POGLUT1 display dual glycosyltransferase activity: O-glucosyltransferase and O-xylosyltransferase activity. In this study, Li et al. performed the structural analysis for human POGLUT1. Although similar structural analysis was recently reported by another group (Yu et al. Nat. Chem. Biol. 2016), this manuscript reported the new aspects of the structures of POGLUT1 in complex with EGF domain and donor analogues, which is different from those reported by the other group. The newly obtained structure led authors to propose the model how POGUT1 catalyzes the transfer of both glucose and xylose.

1. Although this manuscript focused on explaining the dual donor substrate specificity for POGLUT1, the biological significance of xylosyltransferase activity has not been provided. Lack of biological significance of Notch O-xylosylation makes the proposed model for UDP-xylose complex less significant. I understand that addressing the biological function of O-xylose is beyond the scope of this manuscript. However, authors should justify why providing a mechanistic explanation for dual donor substrate specificity would be important.

R3.1) Clear biological roles for POGLUT1-mediated xylosylation have not been elucidated and to that extent, we agree that our mechanistic analysis lacks the significance that it might otherwise have. However, in our revised manuscript we now provide data that shows that a diserine-containing motif is not required for O-xylosylation (contrary to that previously published), and that at least an additional 36%

of POGLUT1 substrates (ie. those containing the C^1XSNPC^2 motif) can be xylosylated. Moreover, the vast majority of glycosyltransferases can only transfer a single donor substrate and our structural and modelling work suggests that POGLUT1 has evolved the ability to access two different local conformations so that it can transfer two different substrates, an intriguing observation in its own right. Given that the hydroxymethyl group of glucose is buried and makes key interactions with POGLUT1, it is certainly possible that two conformational states are required to ensure that both UDP-glucose and UDP-xylose are transferred with similar catalytic efficiencies, as we discuss. Taken together, our results will hopefully stimulate research aimed at determining the extent to which proteins are O-xylosylated by POGLUT1 and, in turn, the role that this modification might play *in vivo*.

2. It has been reported that O-xylosylation by POGLUT1 requires a diserine motif, CXSSPC (Takeuchi H et al. PNAS 2011). Does the UDP-xylose ternary complex provide insights why the diserine motif is important for O-xylosylation?

R3.2) As discussed above (R1.1d, R2.13 and R3.1), we have now shown that POLGUT1 does not require a diserine-containing motif for xylosylation. This is consistent with the observation that the side chain of the residue at the Xb position (the site of the second serine) is hydrogen bonded to POGLUT1 in our structures and does not make contact with the donor substrate.

3. Authors proposed POGLUT1 recognizes the structure in 5-resudue C2C3 loop. Although it is quit interesting, the supporting data are limited to the sequence analysis (SI Figure 1). It would be more convincing if authors could provide biochemical data by altering the number of residues in C2C3 loop of certain EGF domains such as EGF12.

R3.3) To address this comment, we mutated EGF12 to generate C^2C^3 loop variants containing 4, 6, or 7 residues. We assayed these mutants and showed that both POGLUT1 and dRUMI show a very strong preference for a 5-residue C^2C^3 loop. Using database mining we now show that the vast majority of POGLUT1 substrates belong to the hEGF type, and that a 5-residue C^2C^3 loop is characteristic of hEGFs. As we have shown (*in press*), POFUT1 shows a strict dependence on a 5-residue C^2C^3 loop (the site of POFUT1 fucosylation) and, moreover, it appears to modify only hEGFs. In our revised POGLUT1 manuscript, we discuss the potential significance of a shared and conserved determinant of recognition in evolutionary terms.

4. In Figure 3G, it would be more comparable if authors show the UDP-2F-glucose Michaelis complex instead of the UDP-CH2-glucose complex because UDP-2F-glucose behaves as a natural POGLUT1 donor substrate.

R3.4) The original Figure 3G showed the superimposition of two crystal structures, that of the UDP-CH_2-glucose complex and that of the transferred 2F-glucose. We didn't obtain a UDP-2F-glucose structure as the 2F-glucose had all been transferred in the crystal. The UDP-CH_2-glucose compound differs from UDP-glucose only by the replacement of the glycosidic oxygen by the CH_2 group and, as such, it provides the best representation of the Michaelis complex that we have. The contents of Figure 3 are now distributed over Figures 3 and 4 in the revised manuscript. We have also simplified the original Figure 3g.

5. What does EGF(+) stand for ? (eg. line 109)

R3.5) EGF(+) is a synthetic EGF-like domain derived from the consensus sequence obtained by an analysis of all human POFUT1 substrates. Likely a consequence of the significant overlap of POFUT1 and POGLUT1/dRUMI substrates (over 50% of POGLUT1/dRUMI substrates are POFUT1 substrates), EGF(+) is also a good POGLUT1 substrate.

The generation of the EGF(+) consensus sequence is described in our POFUT1 paper (*in press*) and we have added a description of it in the text and online methods in the revised POGLUT1 paper.

6. I found that the supplemental document explaining the difference between this manuscript and the one by Yu et al. is quite important to understand the importance of this manuscript. These information should be provided in the discussion.

R3.6) We have now significantly expanded the results and discussion sections to place this work and that of the Yu *et al* dRUMI paper, as well as our POFUT1 paper (*in press*), in the proper context. As a reminder to the reviewers, our POGLUT1 paper was submitted the same day that the Yu *et al* paper was published and we did not have any reference to it in our original submission.

<u>Reviewers' Comments:</u>

Reviewer #1 (Remarks to the Author):

This manuscript by Li and co-workers is a revised version of an earlier submission. This new version of the manuscript is significantly improved by extending the discussion and including new data. The work builds on an is contemporary to the dRumi paper published last year by Lu and coworkers. The authors nicely define the new points raised in this manuscript and focus on the various EGF complexes and how these help define the substrate specificity of POGLUT1 and its preference for serine. The ability to obtain several complexes are a strength of the manuscript. And the kinetics show that a di-serine motif is not required for xylosylation of EGF repeats. While the functional role of this xylosylation is not known the work here helps lay a foundation to understand the specificity of this interesting enzyme. I support publication of this revised manuscript. The work is an advance that adds to and builds on the work of Lu and coworkers.

My only point of concern remains with the interpretation for the proposed basis of POGLUT1 being able to use UDP-xylose. The modeling is clearly speculative in the absence of structures. The docking methods used clearly bias the outcomes. Docking onto the UDP-POGLUT1 complexes and then minimizing the structure should to lead to changes. Where as the other model is much more likely to lead to changes in the model. I do not find that the modeling is conclusive and the authors should recognize the limitations. I would suggest that the authors clearly note that further work will be needed to help clarify this aspect of the manuscript.

Reviewer #2 (Remarks to the Author):

The authors have been addressed my comments. I support the publication of the manuscript in Nature Communications.

Reviewer #3 (Remarks to the Author):

Overall, the revised manuscripts properly addressed the previous questions asked by the reviewer. The authors provided new data refining the previous finding of O-xylose modification of Notch EGF domains by Poglut1. Interestingly, the diserine motif defined by the previous report was dispensable for O-xylosylation by Poglut1, suggesting expanding roles of O-xylose including in Notch-ligand interaction. Considering these new findings, I suggest authors to change the title to “Structural basis of Notch O-glucosylation and O-xylosylation by mammalian protein-O-glucosyltransferase 1 (POGLUT1).

Response to Reviewers' Comments:

Reviewer #1 (Remarks to the Author):

This manuscript by Li and co-workers is a revised version of an earlier submission. This new version of the manuscript is significantly improved by extending the discussion and including new data. The work builds on an is contemporary to the dRumi paper published last year by Lu and coworkers. The authors nicely define the new points raised in this manuscript and focus on the various EGF complexes and how these help define the substrate specificity of POGLUT1 and its preference for serine. The ability to obtain several complexes are a strength of the manuscript. And the kinetics show that a di-serine motif is not required for xylosylation of EGF repeats. While the functional role of this xylosylation is not known the work here helps lay a foundation to understand the specificity of this interesting enzyme. I support publication of this revised manuscript. The work is an advance that adds to and builds on the work of Lu and coworkers.

My only point of concern remains with the interpretation for the proposed basis of POGLUT1 being able to use UDP-xylose. The modeling is clearly speculative in the absence of structures. The docking methods used clearly bias the outcomes. Docking onto the UDP-POGLUT1 complexes and then minimizing the structure should to lead to changes. Where as the other model is much more likely to lead to changes in the model. I do not find that the modeling is conclusive and the authors should recognize the limitations. I would suggest that the authors clearly note that further work will be needed to help clarify this aspect of the manuscript.

We agree that the modelling that we've done does not prove that UDP-xylose can be bound in the POGLUT1 conformation that we observe in the UDP-containing complexes and that further validation is required.

In addition to the statement:

Taken together, docking and energy minimization supports the suggestion that the POGLUT1 conformation observed in the UDP-containing complexes is responsible for UDP-xylose binding/catalysis.

We have now added:

However, further structural analysis will be required to validate that this is the case.

Reviewer #2 (Remarks to the Author):

The authors have been addressed my comments. I support the publication of the manuscript in Nature

Communications.

Reviewer #3 (Remarks to the Author):

Overall, the revised manuscripts properly addressed the previous questions asked by the reviewer. The authors provided new data refining the previous finding of O-xylose modification of Notch EGF domains by Poglut1. Interestingly, the diserine motif defined by the previous report was dispensable for O-xylosylation by Poglut1, suggesting expanding roles of O-xylose including in Notch-ligand interaction. Considering these new findings, I suggest authors to change the title to "Structural basis of Notch O-glucosylation and O-xylosylation by mammalian protein-O-glucosyltransferase 1 (POGLUT1).

We agree that adding "O-xylosylation" to the title more accurately reflects the scope of our paper, and we have done so in the final revision.
